# Progesterone modulates the *DSCAM-AS1/miR-130a/ESR1* axis to suppress cell invasion and migration in breast cancer

**DOI:** 10.1186/s13058-022-01597-x

**Published:** 2022-12-28

**Authors:** Neelima Yadav, Roma Sunder, Sanket Desai, Bhasker Dharavath, Pratik Chandrani, Mukul Godbole, Amit Dutt

**Affiliations:** 1 Integrated Cancer Genomics Laboratory, Advanced Centre for Treatment, Research, and Education in Cancer (ACTREC), Tata Memorial Centre, Kharghar, Navi Mumbai, Maharashtra 410210 India; 2 Homi Bhabha National Institute, Training School Complex, Anushakti Nagar, Mumbai, Maharashtra 400094 India; 3 Medical Oncology Molecular Lab & Centre for Computational Biology, Bioinformatics and Crosstalk Lab, Tata Memorial Centre, Mumbai, Maharashtra 410210 India; 4 School of Biosciences and Technology, Faculty of Sciences and Health Sciences, MIT World Peace University, Pune, Maharashtra 411038 India

**Keywords:** Breast cancer, *DSCAM-AS1*, Estrogen receptor, *miR-130a*, Progesterone, Progesterone receptor

## Abstract

**Background:**

A preoperative-progesterone intervention increases disease-free survival in patients with breast cancer, with an unknown underlying mechanism. We elucidated the role of non-coding RNAs in response to progesterone in human breast cancer.

**Methods:**

Whole transcriptome sequencing dataset of 30 breast primary tumors (10 tumors exposed to hydroxyprogesterone and 20 tumors as control) were re-analyzed to identify differentially expressed non-coding RNAs followed by real-time PCR analyses to validate the expression of candidates. Functional analyses were performed by genetic knockdown, biochemical, and cell-based assays.

**Results:**

We identified a significant downregulation in the expression of a long non-coding RNA, *Down syndrome cell adhesion molecule antisense DSCAM-AS1*, in response to progesterone treatment in breast cancer. The progesterone-induced expression of *DSCAM-AS1* could be effectively blocked by the knockdown of progesterone receptor (PR) or treatment of cells with mifepristone (PR-antagonist). We further show that knockdown of *DSCAM-AS1* mimics the effect of progesterone in impeding cell migration and invasion in PR-positive breast cancer cells, while its overexpression shows an opposite effect. Additionally, *DSCAM-AS1* sponges the activity of *miR-130a* that regulates the expression of *ESR1* by binding to its 3’-UTR to mediate the effect of progesterone in breast cancer cells. Consistent with our findings, TCGA analysis suggests that high levels of *miR-130a* correlate with a tendency toward better overall survival in patients with breast cancer.

**Conclusion:**

This study presents a mechanism involving the *DSCAM-AS1/miR-130a/ESR1* genomic axis through which progesterone impedes breast cancer cell invasion and migration. The findings highlight the utility of progesterone treatment in impeding metastasis and improving survival outcomes in patients with breast cancer.

**Supplementary Information:**

The online version contains supplementary material available at 10.1186/s13058-022-01597-x.

## Introduction

Progesterone and estrogen, naturally occurring hormones, are known to modulate the progression and disease outcome of breast cancer [1–3]. Approximately 70% of breast cancer patients—positive for estrogen receptor (ER) and progesterone receptor (PR)—receive hormone therapy, such as blocking ER to inhibit estrogen signaling, as the first-line treatment for patients with luminal breast cancer [4, 5]. Previous studies have highlighted the beneficial effects of the progesterone-high luteal phase on surgical outcomes in patients with breast cancer [6–8]. However, how progesterone modulates the downstream signaling remains sparsely understood.

The role of ER has been extensively studied in breast cancer due to its prognostic significance [9, 10], along with its role in increasing the invasion and migration of breast cancer cells [11]. The PR, on the other hand, is a known ER target. The presence of PR is described as an indication of ER activity [12]. In vitro studies suggest that progesterone inhibits the invasion and migration of breast cancer cells [13, 14]. Progesterone also induces cell cycle arrest and mild apoptosis in the cells mediated by PR that can function as a transcription factor to induce gene expression [15–17]. Additionally, PR alters ER binding sites in the genome in response to progesterone, and thus, could modify the expression pattern of ER-responsive genes in breast cancer cells [18].

Long non-coding RNAs (lncRNAs) and microRNAs (miRNAs), non-coding RNAs (ncRNAs), perform diverse regulation of cellular functions by regulating gene expression at transcriptional and post-transcriptional levels [19–25]. For instance, ER regulates the expression of numerous lncRNAs that control cell invasion, migration, proliferation, and apoptosis in response to estrogen [26–28]. Similarly, progesterone regulates the expression of microRNAs in breast cancer cells [29]. The lncRNAs function as competitive endogenous RNAs (ceRNA) or miRNA sponges to regulate miRNA functions in cancer cells [30, 31]. Several studies have identified ceRNA activity of lncRNAs, such as *HULC* [32], *HOTAIR* [33, 34], *TRPM2-AS* [35], and *SNHG7* [36]. However, whether progesterone modulates the expression of lncRNAs in breast cancer cells remains unknown.

Here, we identify *DSCAM-AS1* as a progesterone-responsive lncRNAs in breast cancer using an integrated functional genomics approach. *DSCAM-AS1* acts as a sponge for *miR-130a* to regulate the expression of *ESR1* in hormonal receptor-positive breast cancer cells. The study also suggests that targeting these ncRNAs may help improve survival outcomes in patients with breast cancer.

## Materials and methods

### Transcriptome analysis of breast cancer patient samples

Whole transcriptome sequencing data from 30 breast tumors samples were re-analyzed. Ten tumors were derived from patients who were administered a single dose of 500 mg of hydroxyprogesterone within 15 days prior to surgery, with varying duration for individual patients, while 20 tumors were obtained from patients who were not exposed to hydroxyprogesterone [37]. Gene expression was quantified using Salmon [38]. Genes with expression > 5 reads in at least 20% of the cancer samples were retained. Design matrices were created based on progesterone treatment, and differential gene expression analyses were performed with progesterone-treated (*n* = 10) and control (*n* = 20) tumor samples, using DESeq2 [39]. Data were assessed in the R environment. ENSEMBL IDs were converted using bioconductor packages (org.Hs.eg.db), and gene names not matching the ENSEMBL IDs were obtained from LNCipedia.

### Tissue culture and cancer cell line maintenance

T47-D, BT-474, MCF7 and MDA-MB-231 breast cancer cells were procured, confirmed, cultured, and maintained as explained previously [13, 40]. The human embryonic kidney 293FT cell line was purchased from Invitrogen (Cat No. R70007), cultured in DMEM with 10% FBS, and maintained at 37℃ with 5% CO_2_.

### Progesterone and mifepristone treatment, RNA isolation, cDNA synthesis, and qPCR

Cells were serum-starved and treated with 10 nM progesterone (6 h), 100 nM mifepristone (2 h), or an equal volume of ethanol (vehicle control) as explained previously [13]. RNA isolation, DNaseI treatment, and cDNA synthesis for genes/lncRNAs and microRNAs were performed as explained previously [29, 40]. Further, the cDNAs were used to study gene/miRNA expression patterns by quantitative real-time PCR (qPCR) method using the KAPA SYBR real-time PCR master mix (Sigma, Cat No. KK4601) and QuantStudio 5 real-time PCR system (Applied Biosystems, Cat no. A34322). *GAPDH* or *ACTB* and *U6* were used as internal controls to normalize the expression of genes and miRNAs, respectively. Differential gene expression changes were calculated as fold change values using the 2^−∆∆CT^ method. The sequences for qPCR primers were manually designed using SnapGene sequence viewer. Designed primers were tested and optimized using OligoCalc (Sigma), UCSC In Silico PCR, and NCBI blast. The primer sequences for the genes and miRNAs are listed in Additional file 2: Table S1.

### RNA-sequencing of progesterone-treated breast cancer cells

Total RNA was isolated from progesterone treated and untreated T47-D and MDA-MB-231 cells. Good quality RNA samples (RNA integration number > 9) were used to prepare the sequencing library using TruSeq library prep kit v2 (Illumina) with ribosomal RNA depletion. Libraries were sequenced on HiSeq4000 with 100 bp pair-end chemistry. A minimum of 60 million paired-end reads were obtained for each RNA sample with good Phred scores (score > 30). Differential gene expression analysis was performed using the salmon-DeSeq2 pipeline. Briefly, all the raw reads were corrected using the trimmomatic version V0.32 [41], followed by alignment to the human reference pseudo-genome (GRCh38) using Salmon (version: 0.8.2) [38] and differential expression analysis using DeSeq2 [39]. Genes/lncRNAs with fold change > 2 and < 0.5 with *p*-value < 0.05 were considered to be significantly deregulated in response to progesterone.

### ChIP-sequencing data analysis

ChIP-sequencing data with PR, ER, and p300 pulldown for progesterone-treated T47-D cell line were downloaded from the SRA database [18]. The raw data for these experiments were analyzed as described earlier [40]. Briefly, reads were aligned to the gencode (v30) human reference genome (GRCh38) using a BWA aligner (version 0.7.17). Peak calling was performed using the MACS tool (version 2.0) [42]. Aligned reads were used for differential protein binding in the genome using DiffBind (version 3.0) [43]. The 5 kb upstream and downstream regions for annotated genes/lncRNAs were analyzed for PR, ER, and p300 binding, and annotation of the peaks was performed using Uropa [44].

### Bioinformatics analysis for miRNA binding prediction

DIANA-LncBase v2 [45] database was used to predict the binding of miRNAs to *DSCAM-AS1*. Predicted miRNAs binding to *DSCAM-AS1* were further determined using the "microRNA–ncRNA targets” module of MirWalk v2.0 [46], which includes prediction algorithms of miRanda [47], RNAHybrid [48], and Targetscan [49]. Further, miRTarBase [50] and MirWalk v2.0 were mined to extract miRNAs targeting 3’-UTR of *ESR1*.

### siRNA-mediated knockdown

Sense and antisense DNA oligonucleotides with T7 RNA promoter sequences were designed and synthesized by Sigma-Aldrich to prepare siRNAs targeting *DSCAM-AS1*, *PGR*, and *ESR1*. The complete method for synthesis of small RNA transcripts using T7 RNA polymerase has been described previously [51]. Briefly, sense and antisense strands of DNA oligonucleotides with T7 RNA promoter complementary sequences were annealed in a Thermocycler for synthesizing dsDNA. The dsRNAs were subjected to in vitro transcription reaction (37 ℃ for 2 h) using T7 RNA polymerase (Promega, Cat no. P2075) in 1 × T7 Transcription Buffer (Promega, Cat no. P118B). The single-stranded sense and antisense siRNAs were further annealed to prepare double-stranded siRNAs. The complete list of DNA oligonucleotides for siRNA synthesis is provided in Additional file 2: Table S1. Synthesized siRNAs were transfected in breast cancer cells using Lipofectamine 3000 kit (Invitrogen, Cat No. L3000015) in serum-free media. Progesterone treatment was given to transfected cells 48 h post-transfection and collected for downstream analysis.

### Overexpression of genomic elements

For transient overexpression of *miR-130a* in breast cancer cells, the precursor miRNA sequence with 200 bp flanking gene sequence was amplified from T47-D genomic DNA and cloned in pJET1.2/blunt vector (Thermo Scientific, Cat no. K1232) followed by sub-cloning in pcDNA3.1(-) mammalian expression vector under CMV promoter. *Xba*I (NEB, Cat no. R0145) and *Hind*III (NEB, Cat no. R0104) recognition sequences in multiple cloning sites of pcDNA3.1(-) were used for cloning. For transient overexpression of *DSCAM-AS1*, the complete cDNA sequence was cloned in the pcDNA3.1( +) expression vector using *Xba*I and *Bam*HI (NEB, Cat no. R0136). Cloned constructs were confirmed by restriction digestion and Sanger sequencing. Further, the overexpression plasmids were transfected into breast cancer cells using Lipofectamine 3000. Empty pcDNA3.1(-) vector was transfected as vector control. Cells were collected 48 h post-transfection and RNA was extracted. Overexpression was confirmed by qPCR analysis.

For stable overexpression of *DSCAM-AS1* in breast cancer cells, a complete cDNA sequence was cloned in the pBABE-puro expression vector using *Bam*HI and *Sal*I (NEB, Cat no. R0138). Cloning was confirmed using restriction digestion and Sanger sequencing. The primer sequences used for cloning and Sanger sequencing are provided in Additional file 2: Table S1. The 293FT cells were used for transfection and retrovirus production. Transductions were performed for 16 h in T47-D cells, followed by a selection of positive clones using 1 µg/mL puromycin (HiMedia, Cat no.TC198-10MG). Puromycin-resistant clones were further confirmed for *DSCAM-AS1* overexpression by qPCR analysis.

### Transwell cell invasion and migration assay

Transwell cell migration and invasion assays were performed as described previously [13]. Briefly, cell invasion assay was performed with Matrigel loaded onto the inserts in Boyden chambers; while, cell migration assay was performed without Matrigel. The number of cells that migrated or invaded through the membrane was counted and the total fraction of cells was plotted as percent cell migration or invasion, respectively.

### Luciferase reporter assay

Full-length *DSCAM-AS1* cDNA sequence was amplified from T47-D cells and cloned in pJET1.2/blunt vector, followed by sub-cloning in pGL3-promoter vector (Promega, Luciferase expressing vector) downstream to firefly *luciferase* using *Xba*I. *DSCAM-AS1* mutant construct was generated with mutated overlapping primers by site-directed mutagenesis using *Dpn*I (NEB, Cat no. R0176) and PrimeSTAR GXL DNA polymerase (TaKaRa, Cat no. R050B). *DSCAM-AS1* mutant construct contained mutations in *miR-130a* MRE in *DSCAM-AS1* cDNA to prevent *miR-130a* binding. Wild-type and mutated *DSCAM-AS1* constructs were confirmed by Sanger sequencing. A 620 bp fragment of 3’-UTR of *ESR1* with *miR-130a* binding site was amplified from T47-D cDNA and cloned in pJET1.2/blunt vector. The cDNA was sub-cloned in the pGL3-promoter vector using *Xba*I. The primer sequences for cloning and generating mutant construct are provided in Additional file 2: Table S1.

For luciferase assay, 293FT cells (50,000 cells/well) were co-transfected with pGL3-*DSCAM-AS1* (wild-type/mutant) along with pcDNA3.1(-)-*miR-130a* or pcDNA3.1 empty vector using Lipofectamine 3000 kit. Additionally, co-transfections were performed with pGL3-3’-UTR-*ESR1*, pcDNA3.1(-)-*miR-130a*, or pcDNA3.1 empty vector. pEGFP-N2 was transfected to measure transfection efficiency in all wells. Cells were lysed 48 h post-transfection, and luciferase activity was measured using a luminometer (Berthold Luminometer, Germany). Luminescence and fluorescence units were measured from each transfected well. The luciferase activity was calculated by normalization of luminescence units with fluorescence units from the same well and plotted as luciferase activity. Each experiment was performed in triplicates.

### Gene–miRNA correlation analysis

The total RNA and miRNA sequencing data for patients with breast cancer were downloaded from The Cancer Genome Atlas (TCGA). Data from 751 breast cancer samples sequenced for total RNA and miRNAs were considered for further analysis. The samples with normally distributed *DSCAM-AS1* or *ESR1* expression values were segregated into quartiles. The upper and lower quartile samples were compared. The miRNA levels were compared between patients with *ESR1*-high and -low expression (the upper and lower quartiles, respectively). A similar analysis was performed for miRNAs in patients with *DSCAM-AS1*-high and -low expression. The significance of differences between both the groups was calculated using the Wilcoxon–Mann–Whitney test.

### Survival analysis

The TCGA breast cancer samples with high and low miRNA expression were compared for survival outcomes. The KM plotter [52] and GEPIA [53] were used for Kaplan–Meier survival analysis within specified breast cancer groups. Overall and relapse-free survival of patients was calculated based on the levels of lncRNAs and miRNAs in the samples.

### Statistical analysis

GraphPad Prism version 8 (GraphPad Software, La Jolla, CA) was used to calculate statistical significance between different experimental groups in qPCR, cell-based assays, and luciferase reporter assays. The student's unpaired *t*-test was used to investigate statistical significance. A *p*-value < 0.05 was considered to be statistically significant.

## Results

We previously reported that progesterone inhibits breast cancer invasion and migration via the deactivation of several kinases [13, 29, 40]. Here, we describe the regulatory role of non-coding RNAs in response to progesterone to mediate the cellular changes.

### Identifying significantly deregulated lncRNAs in response to progesterone in breast cancer

**Fig. 1:**
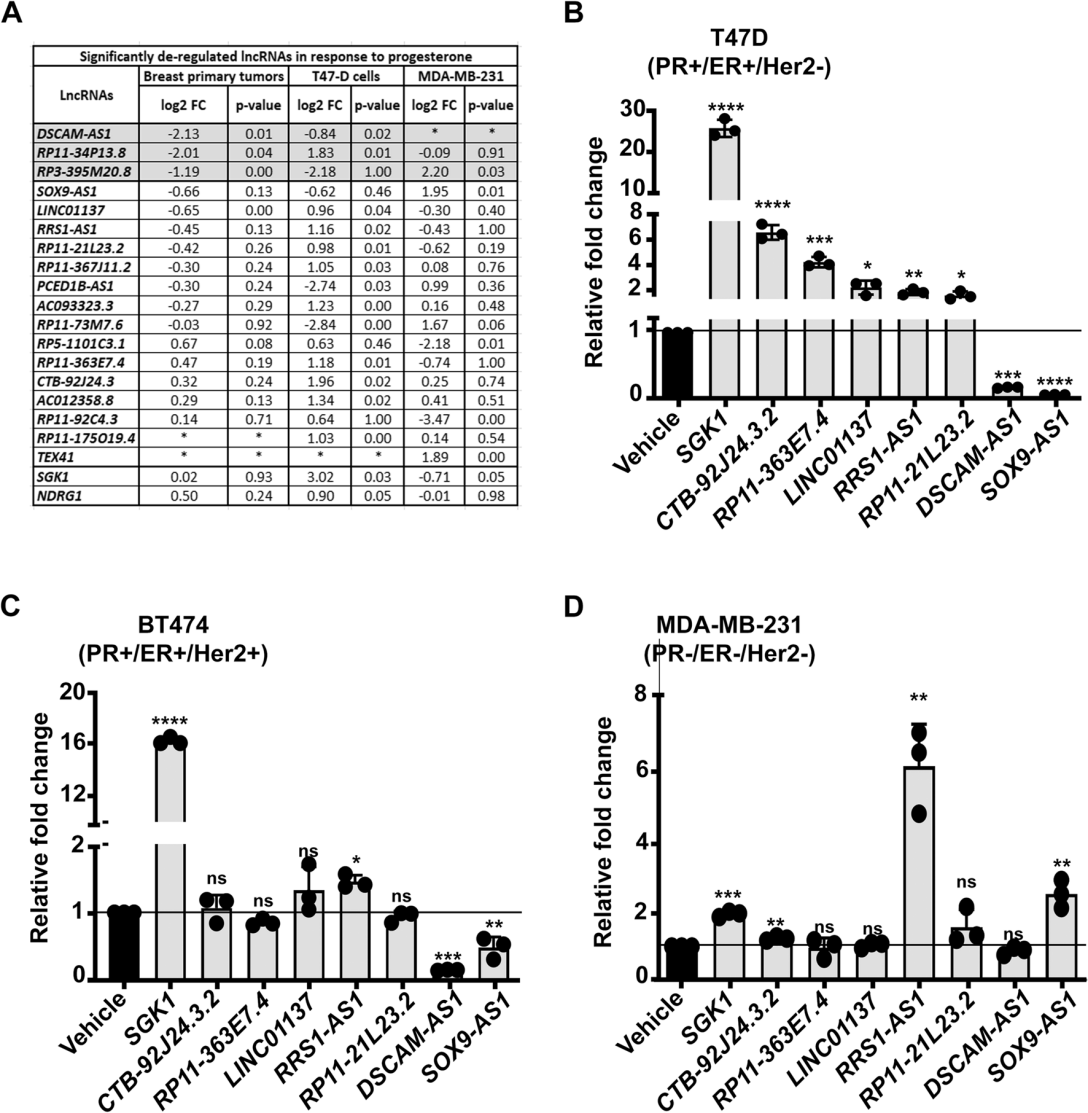
Progesterone deregulates long non-coding RNAs in breast cancer cells. **A** List of significantly deregulated lncRNAs in the transcriptome sequencing data of breast cancer cell lines and patient samples treated with progesterone. Expression fold change upon progesterone treatment is indicated against each lncRNA. lncRNAs downregulated in primary tumors upon progesterone treatment are highlighted in gray shade. *Represents no expression of the gene in breast primary tumor samples. **B**–**D** Real-time PCR analysis of differentially expressed lncRNAs in **B** T47-D, **C** BT-474, and **D** MDA-MB-231 breast cancer cells treated with progesterone. The expression of lncRNAs is normalized with that of *GAPDH* in the same sample. Changes in the normalized expression of lncRNAs upon treatment are plotted as relative fold change (2^−ΔΔCT^) with respect to expression in vehicle control for the same cell line. This consists of data from three biological replicates. The horizontal black line represents a normalized expression of lncRNAs in vehicle-treated cells. *SGK1*, a progesterone-responsive gene, is used as a positive control. *p*-value calculated using Student’s *t*-test. *p < 0.05; **p < 0.01; ***p < 0.001; ****p < 0.0001; *ns* non-significant

First, we analyzed 30 whole transcriptome datasets to identify differentially expressed lncRNAs upon progesterone treatment. Of the 30 tumor samples, 10 had received a single 500 mg dose of hydroxyprogesterone and 20 were controls [37, 54]. Sequencing of these samples generated 17.2–60.7 million reads per sample (median, 37.4 million), wherein > 94–96% reads aligned to the human genome. Differential gene expression analysis between the control and progesterone-treated patients aided in identifying 2,222 differentially expressed genes (FDR < 0.1; 764 up- and 1,458 down-regulated), containing 537 lncRNAs (287 up- and 250 down-regulated), while a majority of the deregulated genes were of protein-coding category (Fig. 1A, Additional file 2: Table S2).

Further, to better understand the underlying mechanisms of action of progesterone, we performed whole transcriptome sequencing of T47-D (PR + /ER + /Her2-) and MDA-MB-231 (PR-/ER-/Her2-) breast cancer cells in response to progesterone treatment. A minimum of 60 million pair-end reads were obtained for each sample with > 90% of reads with a Phred score > 30, suggesting good quality of the data. Of 382 and 206 differentially expressed genes in T47-D and MDA-MB-231 cells, respectively, 18 lncRNAs were significantly deregulated in response to progesterone (-1 < log2FC > 1; *p*-value < 0.05) (Fig. 1A; Additional file 1: Figure S1; Additional file 2: Tables S3, S4). MDA-MB-231, a PR-negative cell line, also showed active transcriptional response to progesterone treatment, likely due to the PR-independent mode of action of progesterone mediated by glucocorticoid receptor (GR) [40]. Interestingly, expression of a few lncRNAs was consistently deregulated in the progesterone-treated breast tumor and cell line transcriptome data, viz.*., DSCAM-AS1, PCED1B-AS1, RP11-21L23.2, RP11-363E7.4*, and *AC012358.8* (Fig. 1A). Of these, expression of *DSCAM-AS1* was considerably downregulated in progesterone-treated breast cancer patients transcriptome data. Moreover, the normalized *DSCAM-AS1* expression in progesterone untreated samples range from 3–860, compared to 3–450 in progesterone treated samples (Additional file 1: Figure S2). This suggests the variable *DSCAM-AS1* expression across progesterone treated and untreated primary breast tumor samples. Further, consistent with our previous study, *SGK1* was found to be significantly upregulated [13, 29, 40], in addition to deregulated expression of some lncRNAs, in progesterone-treated breast cancer samples (Fig. 1A). Taken together, the transcriptome analyses of tumor and cell lines identified novel progesterone-responsive lncRNAs in breast cancer.

### Progesterone downregulates the expression of *DSCAM-AS1* to suppress migration and invasion of PR-positive breast cancer cells

**Fig. 2:**
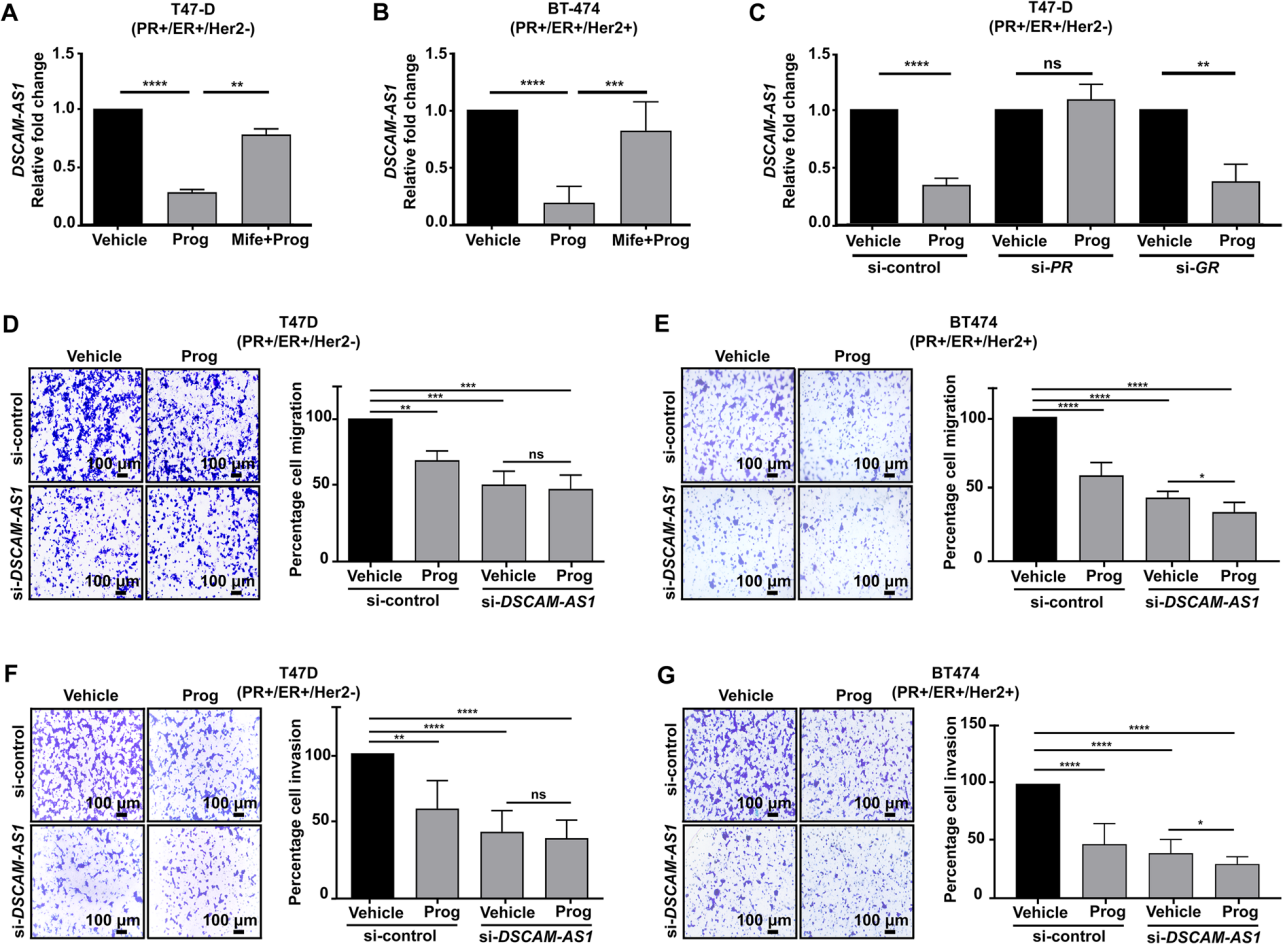
Progesterone downregulates *DSCAM-AS1* via progesterone receptor to suppress invasion and migration of PR-positive breast cancer cells. Real-time PCR analysis of *DSCAM-AS1* in response to progesterone and mifepristone + progesterone in **A** T47-D and **B** BT-474. *DSCAM-AS1* expression is normalized with that of *GAPDH*, and relative fold change values are plotted in comparison to vehicle control. **C** Real-time PCR analysis indicating *DSCAM-AS1* expression upon *PR* and *GR* knockdown followed by progesterone treatment in T47-D cells. Relative fold change is calculated by 2^−ΔΔCT^ and plotted on Y-axis. *p*-value calculated using Student’s *t*-test. Transwell **D**–**E** cell migration and **F**–**G** invasion assay upon siRNA-mediated silencing of *DSCAM-AS1* in T47-D and BT-474 cells. Representative images of crystal violet-stained migrated or invaded cells (10 ×) from each condition are shown. Each bar plot indicates percent cell migration or invasion in each condition with respect to vehicle-treated si-control cells. *p*-value calculated using Student’s *t*-test. *p < 0.05; **p < 0.01; ***p < 0.001; ****p < 0.0001; *ns* non-significant

An orthologous validation of the differentially expressed lncRNAs by real-time PCR identified a long non-coding RNA *Down syndrome cell adhesion molecule antisense*, *DSCAM-AS1*, as downregulated in ER/PR-positive T47-D and BT-474 cells upon progesterone treatment compared to ER/PR-negative MDA-MB-231 cells (Fig. 1B–D). In contrast to T47D and BT474 cells, we detected no significant change in the expression of *DSCAM-AS1* in MCF7 cells (Additional file 1: Figure S3)*,* consistent with its distinct transcriptional landscape, as described earlier, in response to progesterone treatment [18, 55]. The downregulation of *DSCAM-AS1* could be effectively blocked by mifepristone, an antagonist of progesterone receptor (PR) and glucocorticoid receptor (GR) (Fig. 2A, B). However, the siRNA-mediated knockdown of *PR*, but not *GR*, rescued the down-regulation of *DSCAM-AS1* in response to progesterone treatment, suggesting that PR mediates the downregulation of *DSCAM-AS1* in response to progesterone in PR-positive cells (Fig. 2C). Next, we tested whether *DSCAM-AS1* affects the inhibition of migration and invasion ability of breast cancer cells in response to progesterone [13]. Interestingly*, DSCAM-AS1* knockdown could mimic the effect of progesterone by inhibiting breast cancer cell migration and invasion comparable to the extent obtained following treatment of the PR-positive T47-D and BT474 cells with progesterone (Fig. 2D–G).

### *DSCAM-AS1* downregulates the expression of *ESR1* in response to progesterone in PR-positive breast cancer cells

**Fig. 3:**
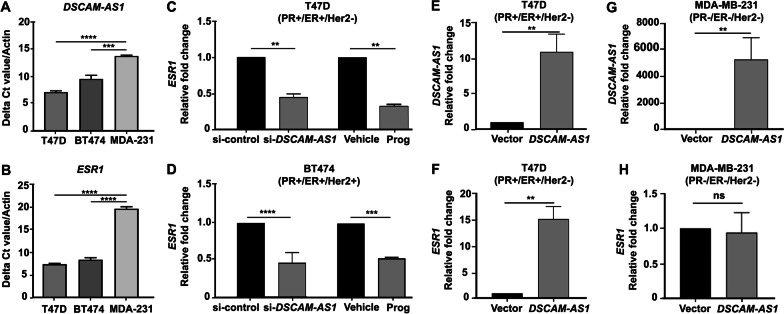
*DSCAM-AS1* regulates *ESR1* levels similar to progesterone treatment in PR-positive breast cancer cells. Real-time PCR analysis indicating normalized expression levels of **A** *DSCAM-AS1* and **B** *ESR1* in breast cancer cell lines with different receptor statuses. Delta Ct value (expression of *DSCAM-AS1* or *ESR1* normalized to that of *GAPDH*) is plotted on Y-axis. The *p*-value is calculated using the student's *t*-test. **C**, **D** Real-time PCR analysis indicating expression of *ESR1* in **C** T47-D and **D** BT-474 cells upon siRNA-mediated silencing of *DSCAM-AS1* and progesterone treatment. Relative fold change with respect to si-control and vehicle treatment is plotted. The expression of *ESR1* is normalized to that of *ACTB*. *p*-value calculated using Student’s *t*-test. **E**, **F** Real-time PCR analyses indicate expression of **E** *DSCAM-AS1* and **F** *ESR1* in T47-D cells upon stable overexpression of *DSCAM-AS1*. Relative fold change in expression of *DSCAM-AS1* and *ESR1* with respect to that of *ACTB* is plotted. *p*-value calculated using Student’s *t*-test. **G**, **H** Real-time PCR analyses indicate expression of **G** *DSCAM-AS1* and **H** *ESR1* in MDA-MB-231 cells upon transient overexpression of *DSCAM-AS1*. Relative fold change in expression of gene with respect to that of *ACTB* is plotted. *p*-value calculated using Student’s *t*-test. *p < 0.05; **p < 0.01; ***p < 0.001; ****p < 0.0001; *ns* non-significant

Estrogen receptor (ER) has previously been shown to regulate *DSCAM-AS1* expression via binding near the promoter region [27]. Consistent with the literature, we observed a significantly higher expression of *DSCAM-AS1* transcript in TCGA breast cancer patient samples and ER/PR-positive T47-D and BT-474 cells than in MDA-MB-231 cells (Fig. 3A, B, Additional file 1: Figure S4). We hypothesized that ER/PR could modulate the *DSCAM-AS1* expression in response to progesterone by binding to its upstream regulatory or distal regions. We analyzed chromatin immunoprecipitation ChIP-sequencing data following progesterone treatment in PR-positive T47-D cells, as described earlier [18, 40]. We identified enrichment of PR, ER, and p300 binding peak upon progesterone treatment at the “region 3” regulatory sequence of *DSCAM-AS1* (Additional file 1: Figure S5). This suggests that progesterone alters the binding occupancy of PR and ER near *DSCAM-AS1*. Surprisingly, siRNA-mediated knockdown of *DSCAM-AS1* in turn led to a significant decrease in the expression of *ESR1* transcript, comparable to progesterone treatment, suggesting a possible feedback mechanism by which *DSCAM-AS1* regulates the expression of *ESR1* in T47-D and BT-474 cells (Fig. 3C, D). In contrast, overexpression of *DSCAM-AS1* in T47-D cells, but not MDA-MB-231 cells, led to overexpression of *ESR1* (Fig. 3E–H), suggesting that progesterone reduces expression of *DSCAM-AS1* that further suppresses expression of *ESR1* to inhibit cell migration and invasion in PR-positive breast cancer cells.

### *DSCAM-AS1* sponges *miR-130a* targeting 3’-UTR of *ESR1* to suppress migration and invasion of PR-positive breast cancer cells

**Fig. 4:**
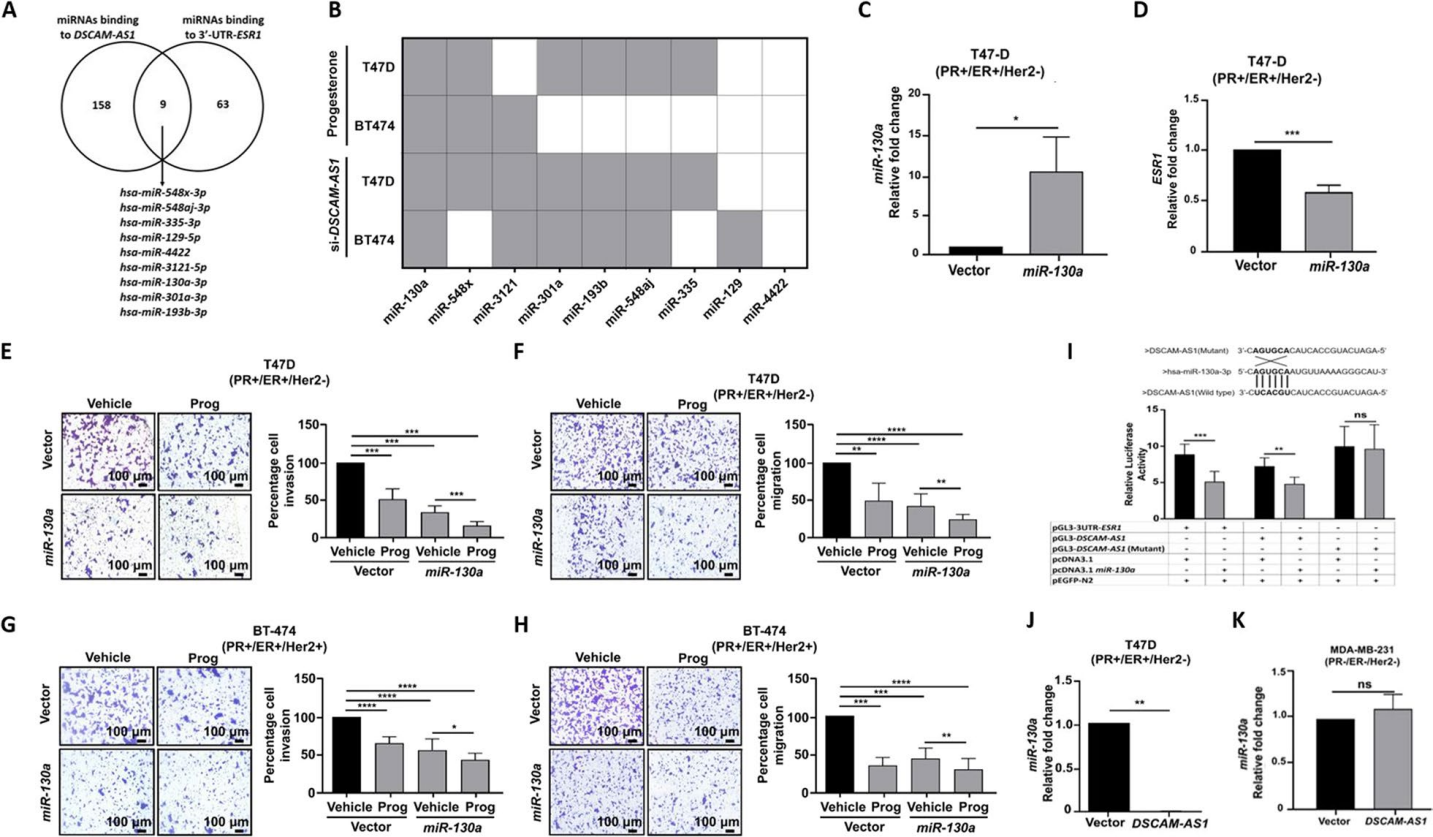
*DSCAM-AS1* sponge *miR-130a* targeting 3’-UTR of *ESR1* to suppress migration and invasion of PR-positive breast cancer cells. **A** Venn diagram depicting miRNAs predicted to target *DSCAM-AS1* and 3’-UTR of *ESR1*. **B** Heatmap representation of real-time PCR analyses indicating significance (*p*-values) of fold change in the 9 miRNAs in response to progesterone and upon silencing *DSCAM-AS1* in T47-D and BT-474 cells. Gray boxes indicate significant up-regulation, while, white boxes indicate no significant change in expression of the miRNAs. The relative expression fold change for miRNAs in response to progesterone is determined with respect to expression in vehicle control or siRNA-control sample. *p*-value calculated using Student’s *t*-test. **C**, **D** Real-time PCR analyses indicating expression of *miR-130a* in **C** T47-D and **D** MDA-MB-231 cells upon overexpression of *DSCAM-AS1*. Expression of *miR-130a* is normalized with respect to that of *U6*. *p*-value calculated using Student’s *t*-test. **E**–**H** Transwell cell invasion and migration assay with ectopic overexpression of *miR-130a* in **E**, **F** T47-D and **G**, **H** BT-474 cells along with progesterone treatment. Cells transfected with an empty vector are used as a control for comparison. Ethanol vehicle treatment is included to compare with progesterone treatment conditions. Percent cell invasion and migration are plotted. *p*-value calculated using Student’s *t*-test. **I** *miR-130a* binding region sequence in *DSCAM-AS1-*wild type and *DSCAM-AS1*-mutant*.* Luciferase reporter activity quantification by co-transfecting plasmids indicated by “ + ”. Relative luciferase activity is calculated by normalizing individual luciferase activity with the GFP signal emitted from the same well. p-GL3-*DSCAM-AS1*-Mutant has the *miR-130a* binding site mutated. The assay is performed in three biological replicates. *p*-value calculated using Student’s *t*-test. **J**, **K** Real-time PCR analysis indicating expression of *miR-130a* upon *DSCAM-AS1* transient overexpression in **J** T47D and **K** MDA-MB-231 cells. Expression of *miR-130a* is normalized with respect to that of *U6*. *p*-value calculated using Student’s *t*-test. *p < 0.05; **p < 0.01; ***p < 0.001; ****p < 0.0001; *ns* non-significant

LncRNAs are known to sponge miRNAs, and thus, reduce the availability of miRNAs for target gene suppression [56, 57]. We thus tested whether *DSCAM-AS1* could sponge miRNAs targeting the 3’-UTR of *ESR1*. Using the DIANA-LncBase v2 database prediction module, we identified 167 miRNAs that could bind *DSCAM-AS1* with miTG-score > 0.7 (Additional file 2: Table S5). Concomitantly, we identified 72 miRNAs predicted to target the 3’-UTR of *ESR1* from the miRTarBase database (Additional file 2: Table S6), with 9 overlapping miRNAs, viz*. miR-548x*, *miR-548aj*, *miR-335*, *miR-129*, *miR-4422*, *miR-3121*, *miR-193b*, *miR-130a*, and *miR-301a* (Fig. 4A). A real-time PCR-based validation of these 9 miRNAs in response to progesterone or genetic knockdown of *DSCAM-AS1* identified *miR-130a* as significantly upregulated in T47-D and BT-474 cells (Fig. 4B, Additional file 1: Figure S6). Interestingly, in BT474 cells, a greater number of miRNAs were downregulated in response to silencing *DSCAM-AS1* than in response to progesterone treatment. This may be related to the de-repression of miRNAs upon silencing *DSCAM-AS1*, a miRNA sponge, as well as the activation and inhibition of various pathways in response to progesterone, such as the up-regulation of *miR-129–2*, which in turn regulates the expression of PR, as demonstrated before [29].

Next, to investigate the function, we ectopically expressed *miR-130a* in T47-D and BT-474 cells. The ectopic expression of *miR-130a* led a significant decrease in *ESR1* transcript than in vector control (Fig. 4C, D), with a concomitant decrease in invasion and migration of T47-D and BT-474 cells. *miR-130a* overexpression could mimic progesterone treatment or *DSCAM-AS1* knockdown in PR-positive T47-D and BT-474 cells (Fig. 4E–H). Furthermore, to test a direct interaction between *miR-130a* and *DSCAM-AS1*, *DSCAM-AS1* cDNA was cloned downstream to the *luciferase* reporter gene. The findings revealed a decrease in normalized luciferase activity upon overexpression of wild-type *miR-130a*, but not with *miR-130a* construct with a mutated binding site. As a positive control, the 3’-UTR of *ESR1* cloned downstream to the *luciferase* gene showed similar inhibition of luciferase activity (Fig. 4I). In contrast, overexpression of *DSCAM-AS1* in T47-D, but not MDA-MB-231, cells led to a significant reduction in *miR-130a* levels than that in vector control (Fig. 4J, K, Additional file 1: Figure S7 A-C). Interestingly, *miR-130a* also showed a significant inverse correlation to *DSCAM-AS1* and *ESR1* expression in 752 TCGA breast cancer samples (Additional file 1: Figure S8 A-B). Taken together, these results validate the association between *DSCAM-AS1* and *miR-130a* to maintain *ESR1* levels in PR-positive breast cancer cells with a consistent inverse correlation of *miR-130a* with the expression of *DSCAM-AS1* and *ESR1* in the TCGA patient samples.

### Upregulation of *miR-130a* correlates with better survival outcome in breast cancer patients

**Fig. 5:**
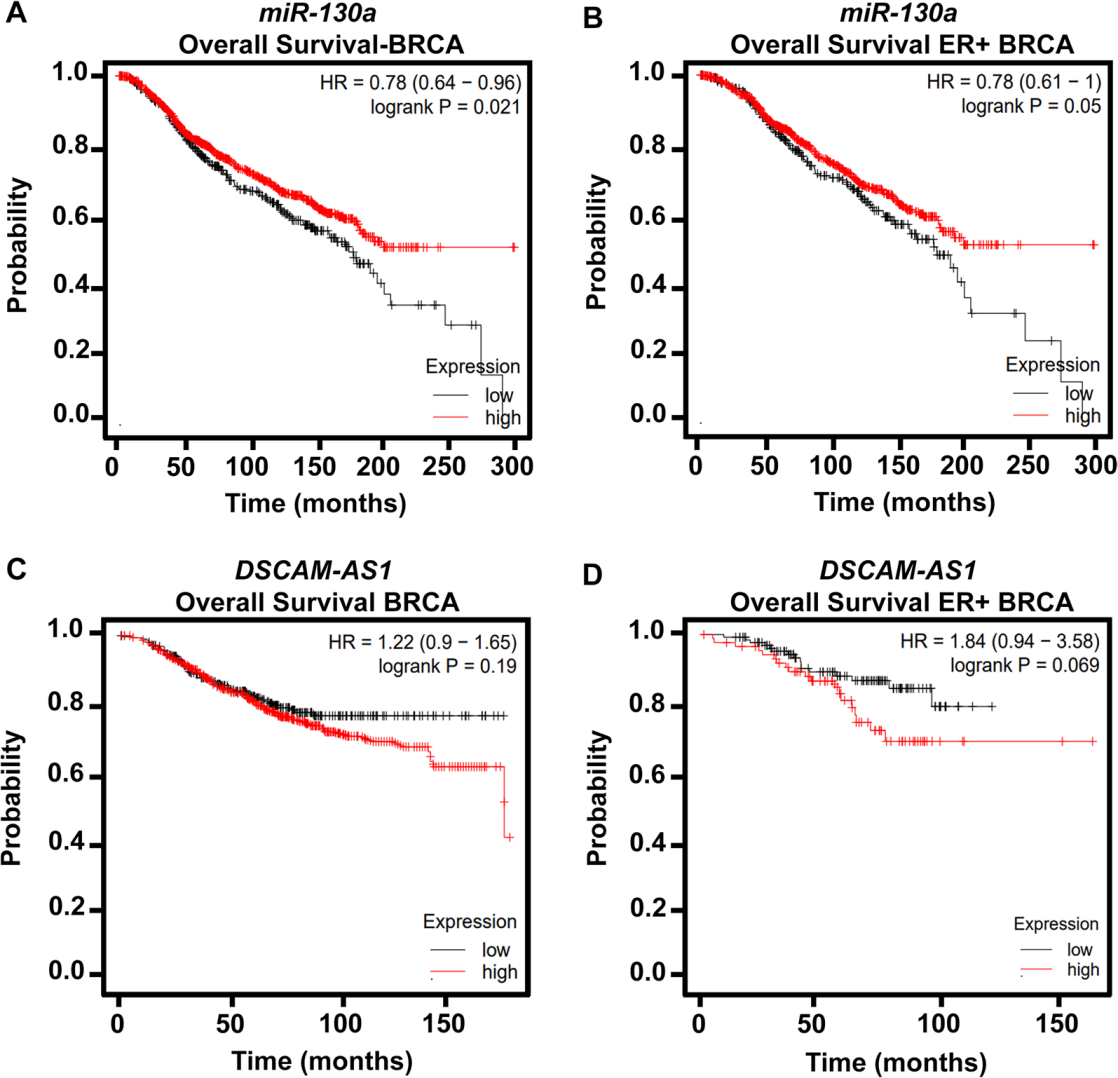
Upregulation of *miR-130a* correlates with a tendency toward better survival outcome in breast cancer patients. **A**, **B** Kaplan–Meier (KM) survival curves indicate differences in overall survival based on *miR-130a* high and low levels in patients with breast carcinoma (BRCA) of **A** all subtypes and **B** estrogen receptor (ER)-positive subtype. **C**, **D** KM survival plots indicate differences in overall survival based on *DSCAM-AS1* high and low levels in patients with **C** all BRCA subtypes and **D** ER-positive subtypes. The probability of survival is plotted on Y-axis and survival time (in months) is represented on X-axis. The red curve represents survival probability in patients with high expression; whereas, the black curve represents that in patients with low expression. Log-rank *p* < 0.05 is considered the cutoff for calculating the significance value. The hazard ratio (HR) for each KM plot is also denoted

The prognostic value of *DSCAM-AS1* and *miR-130a* expression in survival prediction was further tested in TCGA breast cancer datasets (n = 1062) generated by whole transcriptome sequencing to perform the Kaplan–Meier (KM) survival analysis. Patients in the datasets were divided into high- and low-expression classes by the median expression value of *DSCAM-AS1* and *miR-130a*, and a log-rank test was performed for stratifying patients with different prognoses. The analysis showed a significantly better overall survival in patients with breast carcinoma with high *miR-130a* expression than those with low *miR-130a* expression (log-rank *p* = 0.02). Patients with high expression of *miR-130a* survived better (87 months) than those with low expression of *miR-130a* (69 months). Overall, we observed a survival benefit of 18 months in the *miR-130a* high expression cohort. Similar results were observed in patients with ER-positive subtype cancer (log-rank *p* = 0.05) (Fig. 5A, B). In contrast, KM analysis of patients with breast cancer did not show statistically significant change in overall survival in patients who exhibit high and low levels of *DSCAM-AS1* (Fig. 5C, D). These findings imply that a high expression of *miR-130a* influence survival of patients with breast cancer.

## Discussion

Progesterone confers better survival outcomes in patients with breast cancer, especially in those with lymph node involvement [58]. These early clinical observations have increased interest in researchers globally to investigate the mechanisms by which progesterone affects breast cancer pathophysiology. We have previously shown that progesterone reduces breast cancer cell invasion and migration [13] by regulating a tight network of protein-coding genes that reduce the activity of kinases that are known to induce cellular stress [40]. The present study highlights the multiplicity of genomic mediators, especially ncRNAs, recruited by progesterone and PR in breast cancer to abrogate cell invasion and migration.

To begin with, this is the first study to describe progesterone-responsive lncRNAs in breast tumor samples and cell lines. Interestingly, the analyses identified *DSCAM-AS1* as a novel target of progesterone in breast cancer. Progesterone downregulates the expression of *DSCAM-AS1* specifically in PR-positive breast cancer cells, wherein PR modulates the genomic binding pattern of ER, the classical activator of *DSCAM-AS1* [27], in response to progesterone. This also highlights the importance of PR in clinical outcome of breast cancer prognosis and confirms the previous findings that PR modulates ER binding in breast cancer cells treated with progesterone [18, 59]. However, recent report suggests that progesterone treatment may have varied response on tumor growth in patient derived xenograft mouse models [60]. Consistent with this, we also observed variability in *DSCAM-AS1* expression in response to progesterone.

Second, the findings suggest that *DSCAM-AS1* functions as a miRNA sponge to help maintain the high expression of ER in breast cancer cells. *DSCAM-AS1* has previously been shown to function as a miRNA sponge for *miR-101* [61] and *miR-186* [62] in osteosarcoma, and *miR-136* in endometrial cancer [63]. Interestingly, we show that progesterone opposes the *DSCAM-AS-1*–*ESR1* feedback loop, and thus essentially the ER signaling pathway, by employing two synergistic mechanisms—it decreases the expression of *DSCAM-AS1* and increases the expression of *miR-130a* that binds to both *DSCAM-AS1* and 3’UTR of *ESR1* in breast cancer cells. This strengthens the role of progesterone in regulating the expression of non-coding genomic elements in breast cancer [29, 64], in addition to regulating the expression of protein-coding elements. The results of the present study also emphasize the necessity of PR expression in breast cancer cells for progesterone to alter the expression of *DSCAM-AS1* and *miR-130a*, as these effects were not observed in PR-negative MDA-MB-231. Additionally, the expression pattern of *miR-130a* was found to be inversely correlated with that of *ESR1* and *DSCAM-AS1* in cell lines and patients with breast cancer.

Third, the cellular experiments indicated that silencing of *DSCAM-AS1* or overexpression of *miR-130a* led to a significant reduction in breast cancer cell migration and invasion than that in vehicle control cells, comparable to the effect induced by progesterone-alone. Furthermore, progesterone treatment of cells with high *miR-130a* levels led to a greater reduction in cell invasion and migration than progesterone treatment of vehicle-treated control cells; this result demonstrates that variation in expression of these ncRNAs modifies other genomic components that augment the effects of progesterone on breast cancer cells, as described previously [13, 29, 40]. Further, *miR-130a* has been reported to be involved in mitigating progression in breast cancer stem cells [65], and its expression has been reported to be downregulated in breast cancer [66, 67]. Finally, using the TCGA datasets, we show that patients with breast cancer with high *miR-130a* levels correlate with a tendency toward better overall survival (that could not attain statistical significance). Therefore, the findings may help clinicians to better categorize patients with luminal A/B subtype based on the expression of *DSCAM-AS1* or *miR-130a* to receive appropriate care and aid in prolonging their survival outcomes.

In conclusion, this study elucidates an underlying mechanism for a clinical consequence in response to progesterone treatment among patients with breast cancer. Progesterone downregulates the expression of *DSCAM-AS1*, a known ncRNA member of the ER signaling pathway, and increases the expression of *miR-130a* that inhibits *ESR1*, to suppress breast cancer cell invasion and migration. Additionally, high *miR-130a* levels are associated with improved overall survival outcomes in patients with breast cancer, similar to that observed in the randomized controlled trial with preoperative progesterone. Thus, progesterone treatment under hormonal therapy in the adjuvant and neoadjuvant settings may help in impeding cell migration and invasion of breast cancer cells, and in improving the overall and relapse-free survival outcomes in patients with breast cancer.

## Supplementary Information

**Additional file 1**.**Fig. S1**. Differentially expressed genes upon progesterone treatment in breast cancer cell lines **(A, B)** Volcano plot depicting differentially expressed genes upon progesterone treatment in **(A)** T47-D and **(B)** MDA-MB-231 breast cancer cell lines, identified in RNA-sequencing data. X- and Y-axes represent log2(fold change) and -log10(p-value), respectively. Each dot represents expression fold change for an individual gene. All genes above the horizontal red line and outside central blue quadrant are significantly deregulated upon progesterone treatment. The total number of significantly up-regulated and down-regulated genes are represented on the top right and top left of the plot respectively. **Fig. S2.** *DSCAM-AS1* expression in progesterone-treated and -untreated primary breast tumor samples. Gene expression normalization was performed using median of ratios method (DESeq2). The normalized values are plot on Y-axis. X-axis indicates breast cancer patient samples (samples #1 to #30). Median *DSCAM-AS1 *expression in each group is indicated. **Fig. S3.** Real time PCR analysis of differentially expressed lncRNAs in MCF7 cells treated with progesterone. Data are normalized with expression of *GAPDH *and relative fold changes with respect to vehicle control are plotted on Y-axis. Changes in the normalized expression of lncRNAs upon treatment are plotted as relative fold change (2-ΔΔCT) with respect to expression in vehicle control for the same cell line. This consist of data from three biological replicates. The horizontal black line represents normalized expression of lncRNAs in vehicle-treated cells. *SGK1*, a progesterone-responsive gene, is used as a positive control. p-value calculated using Student’s t-test. *, p<0.05; **,p<0.01; ***,p<0.001; ****,p<0.0001; ns, non-significant. **Fig. S4.** *DSCAM-AS1* is up-regulated in breast cancer patient samples **(A)** Box-plot indicating *DSCAM-AS1* expression in breast cancer patients and normal tissue sample data obtained from the TCGA. Red boxes denote the expression of *DSCAM-AS1* in cancer samples, whereas black boxes represent expression in normal tissue samples. **Fig. S5**. Differential binding of PR, ER, and p300 near *DSCAM-AS1* genomic region upon progesterone treatment **(A)** Differential binding of ER, PR, and p300 (histone acetyltransferase) near the *DSCAM-AS1* regulatory regions (within 5 kb upstream and downstream) in T47-D cells upon progesterone treatment. Differential peak calling at each binding location upon progesterone treatment is calculated in three biological replicates. FDR<0.05 is considered the significance value for each peak. **Fig. S6.** Expression of miRNAs in breast cancer cells upon progesterone treatment or *DSCAM-AS1* knockdown. Real time PCR analyses of nine miRNAs upon **(A, B)** progesterone treatment and **(C, D)** *DSCAM-AS1* knockdown in T47D and BT474 cells. Relative fold change of each miRNA with respect to *U6* is plotted on Y-axis. Data are representative of three biological replicates. p is calculated using student’s t-test. p<0.05 is considered to be statistically significant. ns, p>0.05. **Fig. S7.** Transient overexpression of *DSCAM-AS1* reduces *miR-130a* and increases *ESR1 *levels in PR-positive breast cancer cells **(A-C)** Real-time PCR analysis indicating expression of **(A)** *DSCAM-AS1,* **(B)** *ESR1,* and **(C)** *miR-130a* in T47-D cells upon transient overexpression of *DSCAM-AS1*. Relative fold change of expression of gene/lncRNA and *miR130a* with respect to that of *ACTB* and *U6,* respectively, is plotted. p-value calculated using Student’s t-test. *, p<0.05; **,p<0.01; ***,p<0.001; ****,p<0.0001; ns, non-significant. **Fig. S8.** Expression of *miR-130a* is inversely correlated with *DSCAM-AS1 *and *ESR1* expression in the TCGA breast cancer RNA-seq dataset **(A, B)** Expression plot for *miR-130a* in the TCGA breast cancer samples expressing high and low levels of **(A) ***DSCAM-AS1 *and **(B)** *ESR1 *high and low TCGA breast cancer samples. Upper and lower quartile patient groups in terms of *DSCAM-AS1* or *ESR1 *expression are included in the analysis. Normalized expression of *miR-130a *is plotted on Y-axis.

**Additional file 2**.**Table S1.** List of primer sequences. **Table S2.** Differentially expressed genes upon progesterone treatment to breast primary tumors. **Table S3.** Differentially expressed genes upon progesterone treatment to T47D (PR+/ER+/Her2-) cell line. **Table S4.** Differentially expressed genes upon progesterone treatment to MDA-MB-231 (PR-/ER-/Her2-) cell line. **Table S5.** List of miRNAs binding to *DSCAM-AS1. ***Table S6.** List of miRNAs targeting 3'-UTR-*ESR1.*

## Data Availability

The datasets generated and/or analyzed during the current study are available in the ArrayExpress repository under the accession number: E-MTAB-11412.

## Abbreviations

*DSCAM-AS1*: Down Syndrome Cell Adhesion Molecule-Antisense 1
ER: Estrogen receptor
lncRNA: Long non-coding RNA
miR: Micro-RNA
3’-UTR: 3’-Untranslated region
PR: Progesterone receptor
