# Supplementary material for: Progesterone modulates the DSCAM-AS1/miR-130a/ESR1 axis to suppress cell invasion and migration in breast cancer
Source: Breast Cancer Res. 2022 Dec 28;24:97. doi: 10.1186/s13058-022-01597-x (PMC9798554; doi:10.1186/s13058-022-01597-x)
Supplement: Supplementary file 1 — Additional file 1.Fig. S1. Differentially expressed genes upon progesterone treatment in breast cancer cell lines (A, B) Volcano plot depicting differentially expressed genes upon progesterone treatment in (A) T47-D and (B) MDA-MB-231 breast cancer cell lines, identified in RNA-sequencing data. X- and Y-axes represent log2(fold change) and -log10(p-value), respectively. Each dot represents expression fold change for an individual gene. All genes above the horizontal red line and outside central blue quadrant are significantly deregulated upon progesterone treatment. The total number of significantly up-regulated and down-regulated genes are represented on the top right and top left of the plot respectively. Fig. S2. DSCAM-AS1 expression in progesterone-treated and -untreated primary breast tumor samples. Gene expression normalization was performed using median of ratios method (DESeq2). The normalized values are plot on Y-axis. X-axis indicates breast cancer patient samples (samples #1 to #30). Median DSCAM-AS1 expression in each group is indicated. Fig. S3. Real time PCR analysis of differentially expressed lncRNAs in MCF7 cells treated with progesterone. Data are normalized with expression of GAPDH and relative fold changes with respect to vehicle control are plotted on Y-axis. Changes in the normalized expression of lncRNAs upon treatment are plotted as relative fold change (2-ΔΔCT) with respect to expression in vehicle control for the same cell line. This consist of data from three biological replicates. The horizontal black line represents normalized expression of lncRNAs in vehicle-treated cells. SGK1, a progesterone-responsive gene, is used as a positive control. p-value calculated using Student’s t-test. *, p<0.05; **,p<0.01; ***,p<0.001; ****,p<0.0001; ns, non-significant. Fig. S4. DSCAM-AS1 is up-regulated in breast cancer patient samples (A) Box-plot indicating DSCAM-AS1 expression in breast cancer patients and normal tissue sample data obtained from the [file 13058_2022_1597_MOESM1_ESM.pdf]

SUPPLEMENTARY FIGURES

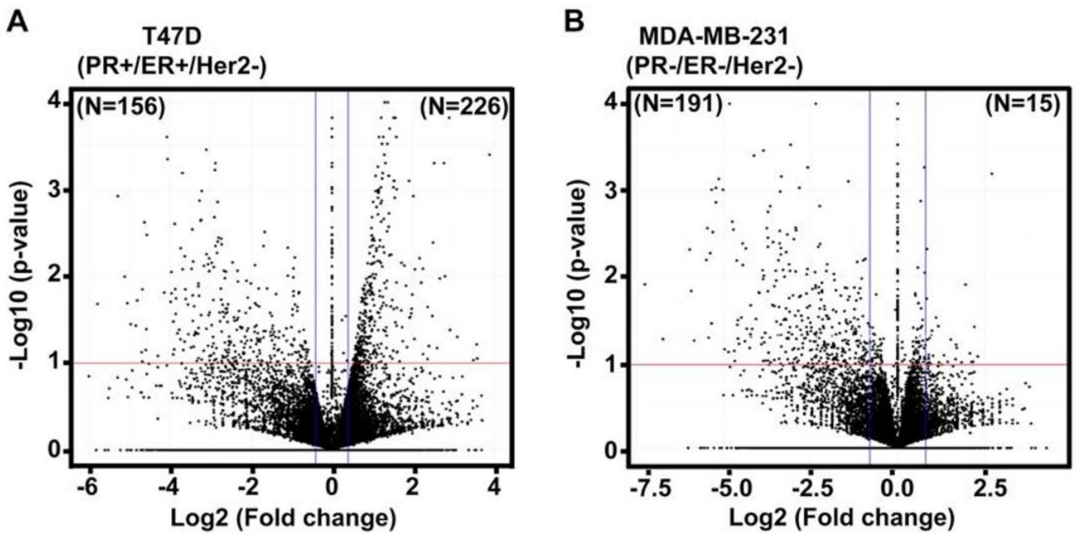

Figure S1: Differentially expressed genes upon progesterone treatment in breast cancer cell lines: (A-B) Volcano plot depicting differentially expressed genes upon progesterone treatment in (A) T47-D and (B) MDA-MB-231 breast cancer cell lines, identified in RNA-sequencing data. X- and Y-axes represent log2(fold change) and -log10(p-value), respectively. Each dot represents expression fold change for an individual gene. All genes above the horizontal red line and outside central blue quadrant are significantly deregulated upon progesterone treatment. The total number of significantly up-regulated and down-regulated genes are represented on the top right and top left of the plot respectively.

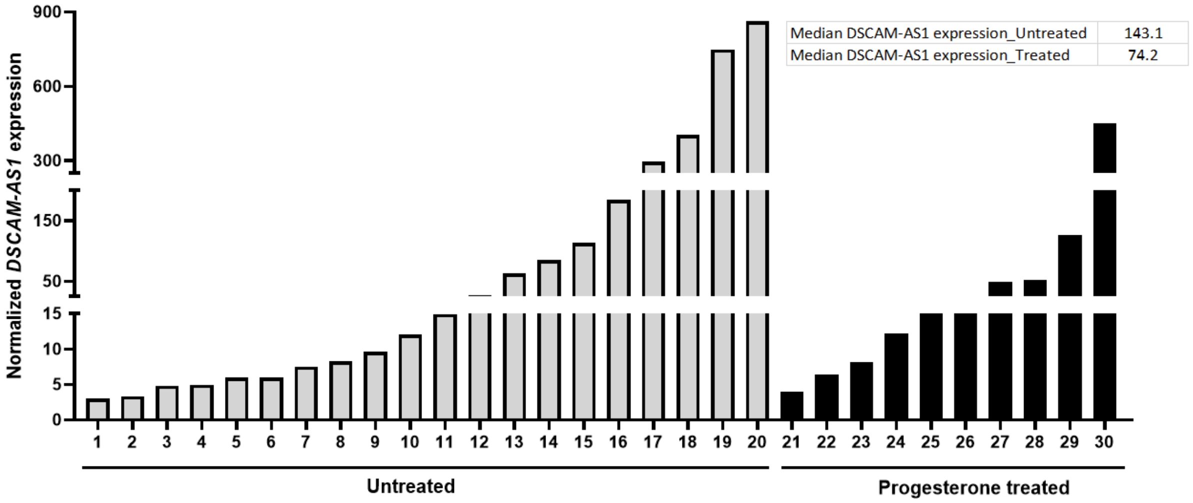

Figure S2: *DSCAM-AS1* expression in progesterone-treated and -untreated primary breast tumor samples. Gene expression normalization was performed using median of ratios method (DESeq2). The normalized values are plot on Y-axis. X-axis indicates breast cancer patient samples (samples #1 to #30). Median *DSCAM-AS1* expression in each group is indicated.

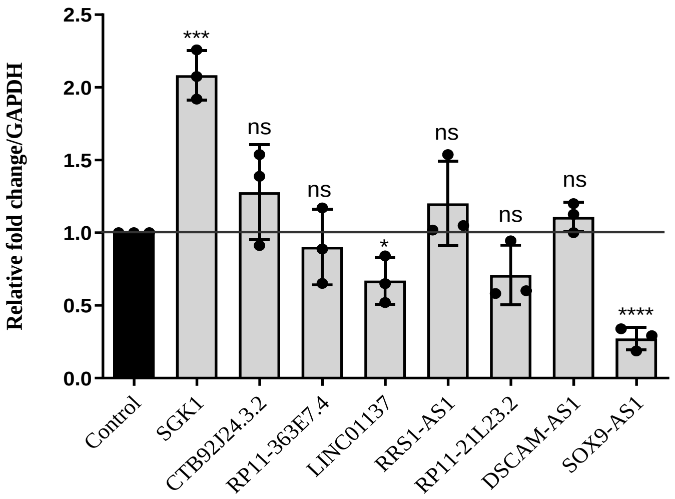

Figure S3: Real time PCR analysis of differentially expressed lncRNAs in MCF7 cells treated with progesterone. Data are normalized with expression of GAPDH and relative fold changes with respect to vehicle control are plotted on Y-axis. Changes in the normalized expression of lncRNAs upon treatment are plotted as relative fold change ($2^{-\Delta\Delta CT}$) with respect to expression in vehicle control for the same cell line. This consist of data from three biological replicates. The horizontal black line represents normalized expression of lncRNAs in vehicle-treated cells. SGK1, a progesterone-responsive gene, is used as a positive control. p-value calculated using Student's t-test. *, p<0.05; **, p<0.01; ***, p<0.001; ****, p<0.0001; ns, non-significant.

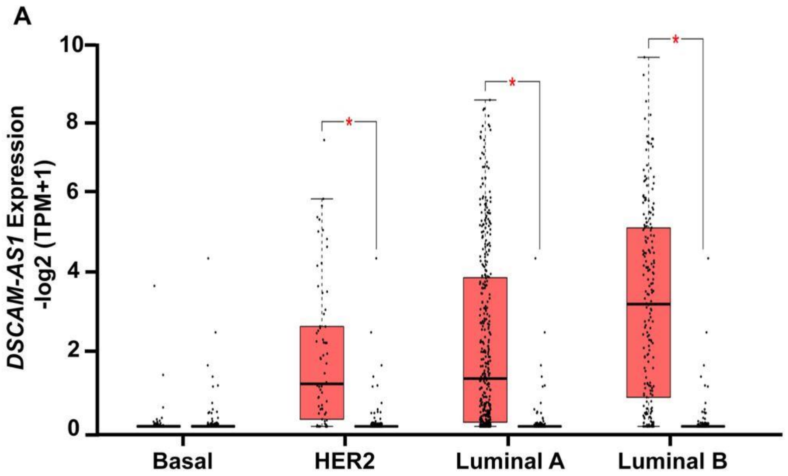

Figure S4: *DSCAM-AS1* is up-regulated in breast cancer patient samples: (A) Box-plot indicating *DSCAM-AS1* expression in breast cancer patients and normal tissue sample data obtained from the TCGA. Red boxes denote the expression of *DSCAM-AS1* in cancer samples, whereas black boxes represent expression in normal tissue samples.

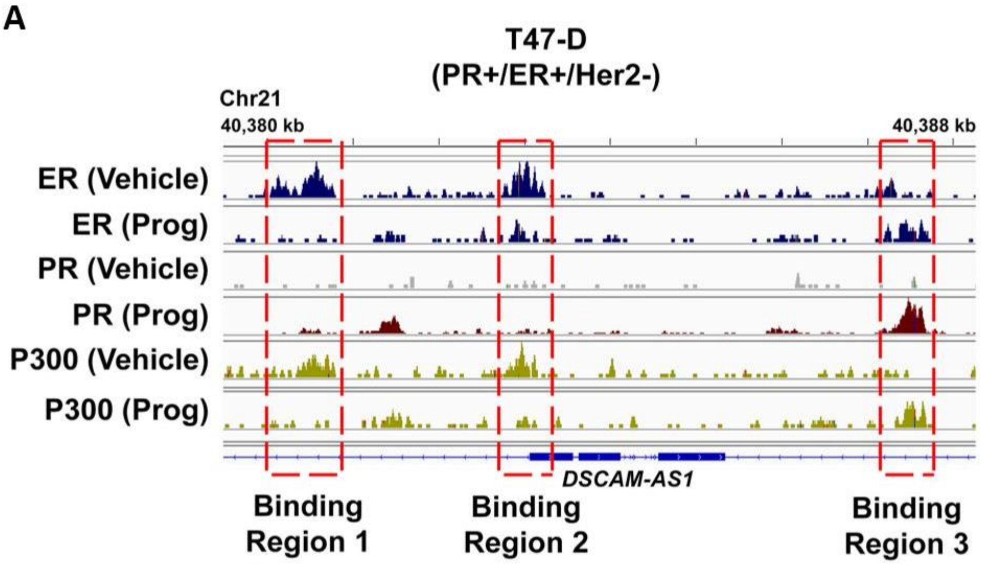

Figure S5: Differential binding of PR, ER, and p300 near *DSCAM-AS1* genomic region upon progesterone treatment: (A) Differential binding of ER, PR, and p300 (histone acetyltransferase) near the *DSCAM-AS1* regulatory regions (within 5 kb upstream and downstream) in T47-D cells upon progesterone treatment. Differential peak calling at each binding location upon progesterone treatment is calculated in three biological replicates. FDR<0.05 is considered the significance value for each peak.

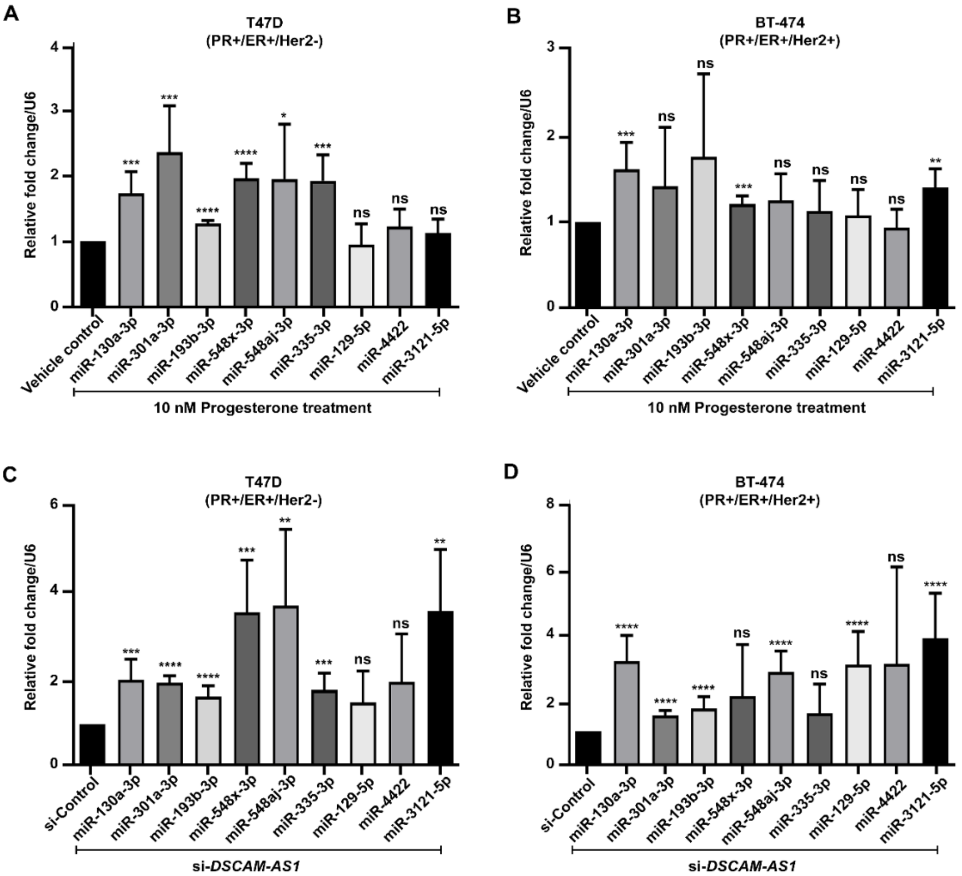

Figure S6: Expression of miRNAs in breast cancer cells upon progesterone treatment or *DSCAM-AS1* knockdown. Real time PCR analyses of nine miRNAs upon (A, B) progesterone treatment and (C, D) *DSCAM-AS1* knockdown in T47D and BT474 cells. Relative fold change of each miRNA with respect to U6 is plotted on Y-axis. Data are representative of three biological replicates. p is calculated using student's t-test. $p<0.05$ is considered to be statistically significant. ns, $p>0.05$.

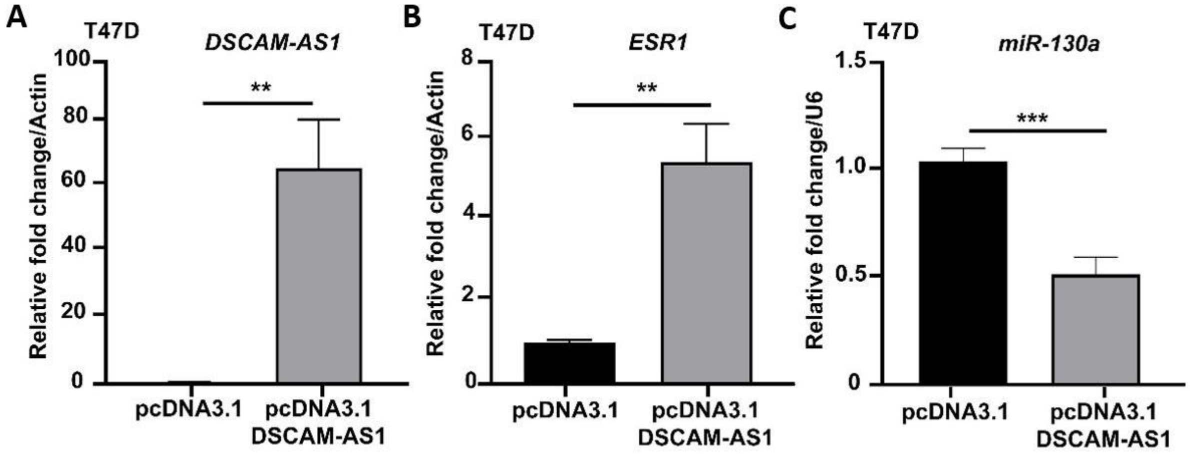

Figure S7: Transient overexpression of *DSCAM-AS1* reduces *miR-130a* and increases *ESR1* levels in PR-positive breast cancer cells: (A-C) Real-time PCR analysis indicating expression of (A) *DSCAM-AS1*, (B) *ESR1*, and (C) *miR-130a* in T47-D cells upon transient overexpression of *DSCAM-AS1*. Relative fold change of expression of gene/lncRNA and *miR130a* with respect to that of *ACTB* and *U6*, respectively, is plotted. *p*-value calculated using Student's *t*-test. *, $p<0.05$; **, $p<0.01$; ***, $p<0.001$; ****, $p<0.0001$; ns, non-significant.

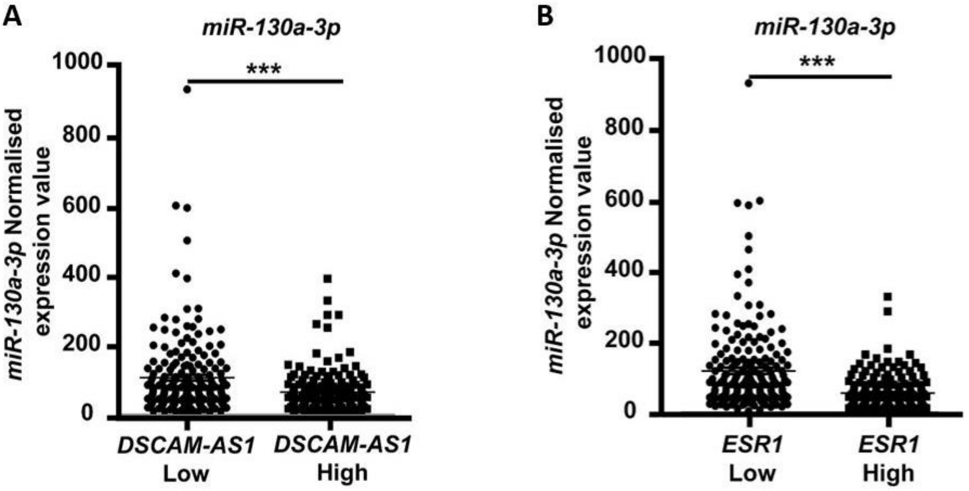

Figure S8: Expression of *miR-130a* is inversely correlated with *DSCAM-AS1* and *ESR1* expression in the TCGA breast cancer RNA-seq dataset: (A-B) Expression plot for *miR-130a* in the TCGA breast cancer samples expressing high and low levels of (A) *DSCAM-AS1* and (B) *ESR1* high and low TCGA breast cancer samples. Upper and lower quartile patient groups in terms of *DSCAM-AS1* or *ESR1* expression are included in the analysis. Normalized expression of *miR-130a* is plotted on Y-axis.
