# Supplementary material for: Progesterone modulates the DSCAM-AS1/miR-130a/ESR1 axis to suppress cell invasion and migration in breast cancer
Source: Breast Cancer Res. 2022 Dec 28;24:97. doi: 10.1186/s13058-022-01597-x (PMC9798554; doi:10.1186/s13058-022-01597-x)
Supplement: Supplementary file 2 — Additional file 2.Table S1. List of primer sequences. Table S2. Differentially expressed genes upon progesterone treatment to breast primary tumors. Table S3. Differentially expressed genes upon progesterone treatment to T47D (PR+/ER+/Her2-) cell line. Table S4. Differentially expressed genes upon progesterone treatment to MDA-MB-231 (PR-/ER-/Her2-) cell line. Table S5. List of miRNAs binding to DSCAM-AS1. Table S6. List of miRNAs targeting 3'-UTR-ESR1. [file 13058_2022_1597_MOESM2_ESM.pdf]

Table S1: List of primer sequences

S.No.	Primer	Primer sequence (5'-3')	Comments
1	OAD2439_*miR-130a-3p* _F	CAGTGCAATGTTAAAAGGGCAT	**primers for screening expression of genes, lncRNAs and miRNAs by real time PCR**
2	OAD2440_*miR-301a-3p* _F	CAGTGCAATAGTATTGTCAAAGC	
3	OAD2441_*miR-193b-3p* _F	AACTGGCCCTCAAAGTCCCGCT	
4	OAD2461_*hsa-miR-548x-3p* _F	TAAAAACTGCAATTACTTTC	
5	OAD2462_*hsa-miR-548aj-3p* _F	TAAAAACTGCAATTACTTTTA	
6	OAD2463_*hsa-miR-335-3p* _F	TTTTTCATTATTGCTCCTGACC	
7	OAD2464_*hsa-miR-129-5p* _F	CTTTTTGCGGTCTGGGCTTGC	
8	OAD2465_*hsa-miR-4422* _F	AAAAGCATCAGGAAGTACCCA	
9	OAD2466_*hsa-miR-3121-5p* _F	TCCTTTGCCTATTCTATTTAAG	
10	OAD1593_*LINC01137* _F	ACAGGTAAAGCCGCGAACACTC	
11	OAD1594_*LINC01137* _R	AAGAGCTTGAGATCAGCCTGGG	
12	OAD1597_*RRS1-AS1* _F	GAGACCAGCGTGAATAAGATGGG	
13	OAD1598_*RRS1-AS1* _R	GTCTCAAACTCCTGACCTCAGGTG	
14	OAD1589_*SOX9-AS1* _F	GGTTTCCATGTCCTTTGGCTTCTG	
15	OAD1590_*SOX9-AS1* _R	ACTGCTCTCATCCAAGGTGAACAG	
16	OAD1575_*CTB-92J24.3.2* _F	ATCTGGCTTGCATCTATGGGAGC	
17	OAD1577_*CTB-92J24.3.2* _R	CATAGTTGGTTGAGTGGCAGCAC	
18	OAD1601_*RP11-363E7.4* _F	GGGAACTCTATGCCTGTCCAGTG	
19	OAD1602_*RP11-363E7.4* _R	CCAGTAAGTGGAATAGGTGGTCG	
20	OAD1595_*RP11-21L23.2* _F	GGTCTTATTCTGTCACCCAGGCT	
21	OAD1596_*RP11-21L23.2* _R	TAGCTGAGCATGGTGACGTATGC	
22	OAD1579_*DSCAM-AS1* _R	CGTCCATCTCTGTTGTCCTGTTG	
23	OAD1580_*DSCAM-AS1* _F	CCACTCACTGACTTAGGTGCCT	
24	OAD 233 5` *SGK1*_ F	GCAGAAGAAGTGTTCTATGCAGT	
25	OAD 234 3` *SGK1*_ R	CCGCTCCGACATAATATGCTT	
26	OAD 131 5` *ESR1*_ F	CCCACTCAACAGCGTGTCTC	
27	OAD 132 3` *ESR1*_ R	CGTCGATTATCTGAATTTGGCCT	
28	OAD 121 5` *PR-A/B*_ F	AGCCCACAATACAGCTTCGAG	
29	OAD 122 3` *PR-A/B*_ R	TTTCGACCTCCAAGGACCAT	
30	OAD 621_*GR* _F	GGACTCTGAACTTCCCTGGTCG	
31	OAD 622_*GR* _R	GCTGTTGAGGAGCTGGATGG	
32	OAD2360_*DSCAMAS1* _si1_Sense	AAGTTCTGGTCTCATCATGATCTATAGTGAGTCGTATTA	**DNA oligos for siRNA synthesis using *in vitro* transcription method**
33	OAD2361_*DSCAMAS1* _si1_Anti-Sense	GGATCATGATGAGACCAGAACCTATAGTGAGTCGTATTA	
34	OAD2362_*DSCAMAS1* _si2_Sense	TCCCTCCTCCAACTGCCATTTCTATAGTGAGTCGTATTA	
35	OAD2363_*DSCAMAS1* _si2_Anti-Sense	ATAAATGGCAGTTGGAGGAGGCTATAGTGAGTCGTATTA	
36	OAD2364_*DSCAMAS1* _si3_Sense	CCCACATAGGCATGACATACTCTATAGTGAGTCGTATTA	
37	OAD2365_*DSCAMAS1* _si3_Anti-Sense	TGAGTATGTCATGCCTATGTGCTATAGTGAGTCGTATTA	
38	OAD2782_*PGR*_ si1_Sense	TACCTCAGAAGATTTGTTTAACTATAGTGAGTCGTATTA	
39	OAD2783_*PGR* _si1_Anti-Sense	GATTAAACAAATCTTCTGAGGCTATAGTGAGTCGTATTA	
40	OAD2784_*PGR* _si2_Sense	TTACATATTGATGACCAGATACTATAGTGAGTCGTATTA	
41	OAD2785_*PGR* _si2_Anti-Sense	GTTATCTGGTCATCAATATGTCTATAGTGAGTCGTATTA	
42	OAD2786_*PGR* _si3_Sense	ATGAACAGCGGATGAAAGAATCTATAGTGAGTCGTATTA	
43	OAD2787_*PGR* _si3_Anti-Sense	TGATTCTTTCATCCGCTGTTCCTATAGTGAGTCGTATTA	
44	OAD2778_Control_siRNA_Sense	ATGAACTTCAGGGTCAGCTTGCTATAGTGAGTCGTATTA	
45	OAD2779_Control_siRNA_Anti-Sense	CGGCAAGCTGACCCTGAAGTTCTATAGTGAGTCGTATTA	
46	OAD2537_*miR-130a* _XbaI_F	TGCTCTAGAAAGGTGGTCTCTGTGCTGGG	**Primers for cloning in pcDNA3.1 vector.**
47	OAD2538_*miR-130a* _HindIII_R	CCCAAGCTTACAGTAACGGAGGCAGTGTC	
48	OAD1624_*DSCAM-AS1* _BamhI_F	CGCGGATCCCTTTGGGAGGCTGAGGCAGGC	
49	OAD2134_*DSCAM-AS1* _XbaI_R	GCTCTAGAACTAAATGCATGCTTGATGGAGC	
50	OAD1624_*DSCAM-AS1* _BamhI_F	CGCGGATCCCTTTGGGAGGCTGAGGCAGGC	**Primers for cloning in p-Babepuro vector.**
51	OAD1625_*DSCAM-AS1* _SalI_R	GCCGTCGACACTAAATGCATGCTTGATGGAGC	
52	OAD1996_*DSCAM-AS1* _F	GCTCTAGACTTTGGGAGGCTGAGGCAGGC	**primers to generate construct for luciferase assay**
53	OAD2134_*DSCAM-AS1* _R	GCTCTAGAACTAAATGCATGCTTGATGGAGC	
54	OAD2763_*DSCAM-AS1* _Mutant_F	GTGAGCTGAGATCATGCCACTACAGTGCACCAGCCTGGGTGACACAGC	
55	OAD2764_*DSCAM-AS1* _Mutant_R	GCTGTGTCACCCAGGCTGGTGCACTGTAGTGGCATGATCTCAGCTCAC	
56	OAD2712_3'-UTR-*ESR1* _F	GCTCTAGAGCACCATTCCCAAGTTAATCCCC	
57	OAD2714_3'-UTR-*ESR1* _R	GCTCTAGAGCTGCGACAAAACCGAGTCAC	

Table S2: Differentially expressed genes upon progesterone treatment to breast primary tun

S.No.	Gene	Log2FoldChange	P-value
1	A1BG	-1.17	0.00
2	AAAS	-0.75	0.00
3	AAMP	-0.70	0.00
4	ABCC13	1.59	0.00
5	ABHD14A	-1.07	0.00
6	ABHD14A-ACY1	-1.29	0.00
7	ABHD14B	-1.68	0.00
8	ABHD16A	-1.21	0.00
9	ABHD17A	-1.23	0.00
10	ACACA	0.83	0.00
11	ACAP2	0.59	0.00
12	ACBD4	-0.83	0.01
13	ACOT7	-1.00	0.01
14	ACOX2	-3.10	0.00
15	ACSM6	0.73	0.01
16	ACTR8	-0.47	0.00
17	ACVR2A	0.75	0.00
18	ACY1	-0.96	0.01
19	ADAM29	0.86	0.01
20	ADAM32	0.63	0.01
21	ADAM32	0.75	0.00
22	ADAMTSL5	-0.87	0.00
23	ADARB1	0.83	0.00
24	ADGRA2	-0.89	0.01
25	ADIRF	-2.92	0.00
26	ADO	-0.58	0.00
27	ADORA1	-1.60	0.00
28	ADPRHL2	-0.86	0.00
29	ADRA2A	-1.45	0.00
30	ADRB3	0.90	0.00
31	AEBP2	0.55	0.00
32	AFAP1L2	-0.98	0.00
33	AGAP2-AS1	-1.65	0.00
34	AGAP5	0.53	0.00
35	AGBL1	1.17	0.01
36	AGBL5	-0.45	0.00
37	AGFG2	-0.74	0.00
38	AGPAT1	-3.89	0.00
39	AGPAT1	-3.89	0.00
40	AGTRAP	-0.94	0.01
41	AHCYL2	0.44	0.01
42	AIP	-0.82	0.00
43	AK1	-0.89	0.00
44	AKAP17A	-0.53	0.01
45	AKIP1	-0.50	0.00
46	ALKBH6	-0.92	0.00
47	AMOTL2	-1.24	0.00

48	ANAPC11	-1.05	0.00
49	ANAPC15	-0.57	0.00
50	ANAPC4	0.45	0.00
51	ANG	-1.07	0.00
52	ANGPTL3	1.65	0.00
53	ANKRD18B	1.10	0.01
54	ANKRD20A1	1.31	0.00
55	ANKRD36	0.75	0.00
56	ANKRD40	-0.52	0.00
57	ANKUB1	0.72	0.01
58	ANXA11	-0.88	0.00
59	ANXA2R	-1.00	0.00
60	AP2S1	-0.87	0.01
61	APBB1	-1.16	0.00
62	APEH	-0.66	0.00
63	APLNR	-0.76	0.00
64	APOBEC3H	-1.05	0.00
65	APOM	-1.07	0.00
66	APRT	-1.54	0.00
67	AQP1	-2.11	0.00
68	ARAF	-0.70	0.00
69	ARHGAP29-AS1	-2.09	0.00
70	ARID1B	0.46	0.00
71	ARIH2OS	-0.63	0.01
72	ARL2	-0.98	0.00
73	ARL6IP4	-0.77	0.00
74	ARMC5	-0.63	0.01
75	ARMC7	-1.28	0.00
76	ARRB2	-0.46	0.00
77	ARRDC1-AS1	-0.51	0.01
78	ASB18	0.88	0.00
79	ASXL1	0.66	0.00
80	ATAD2B	0.54	0.01
81	ATF4	-1.05	0.00
82	ATF5	-0.88	0.00
83	ATL2	0.59	0.01
84	ATP13A3	0.39	0.01
85	ATP1B3	0.51	0.01
86	ATP2C1	0.50	0.00
87	ATP5F1D	-1.04	0.00
88	ATP5IF1	-0.86	0.00
89	ATP5MC1	-1.22	0.00
90	ATP5MC2	-1.47	0.00
91	ATP5MF	-0.74	0.00
92	ATP5MG	-0.82	0.00
93	ATP6V1F	-0.75	0.01
94	ATP9B	0.58	0.00
95	ATR	0.53	0.00
96	ATRX	0.55	0.00
97	ATXN10	0.60	0.01

98	B3GALT6	-1.10	0.00
99	B3GAT2	0.75	0.00
100	B4GALNT2	0.77	0.00
101	B4GALT2	-0.95	0.00
102	B9D1	-0.91	0.01
103	BABAM1	-0.51	0.01
104	BAIAP2-DT	-1.01	0.00
105	BANF1	-1.15	0.00
106	BAP1	-0.56	0.00
107	BARX2	-1.57	0.00
108	BATF	-1.31	0.00
109	BAX	-0.79	0.01
110	BBS9	0.59	0.00
111	BCAR3	-0.82	0.01
112	BCKDK	-0.63	0.01
113	BCL2L12	-1.04	0.00
114	BCL6B	-0.92	0.00
115	BCL7B	-0.53	0.00
116	BCL7C	-0.61	0.00
117	BEX4	-1.04	0.00
118	BIN1	-0.76	0.00
119	BIRC6	0.41	0.01
120	BISPR	-0.67	0.00
121	BLOC1S1	-1.04	0.00
122	BMS1P1	0.57	0.01
123	BOLA1	-0.83	0.00
124	BOLA3-AS1	-0.82	0.00
125	BORCS8	-0.62	0.00
126	BRAT1	-0.78	0.00
127	BRD2	-0.90	0.01
128	BRD3OS	-0.80	0.00
129	BRK1	-0.61	0.01
130	BRMS1	-0.75	0.00
131	BRPF1	-0.74	0.00
132	BSND	0.82	0.01
133	BTBD1	0.37	0.01
134	BUD13	-0.69	0.00
135	C11orf49	0.52	0.00
136	C11orf52	-1.26	0.00
137	C11orf68	-0.75	0.01
138	C11orf71	-0.84	0.00
139	C11orf98	-1.11	0.00
140	C12orf10	-0.78	0.00
141	C12orf40	0.67	0.01
142	C12orf43	-0.49	0.00
143	C12orf57	-1.09	0.00
144	C14orf93	-0.52	0.00
145	C16orf86	-1.02	0.00
146	C17orf100	0.43	0.01
147	C17orf49	-1.06	0.00

148	C17orf97	-1.70	0.00
149	C17orf97	-1.70	0.00
150	C18orf54	0.88	0.00
151	C19orf38	-1.16	0.00
152	C19orf53	-1.81	0.00
153	C19orf67	-0.95	0.00
154	C19orf73	-1.53	0.00
155	C1orf122	-1.76	0.00
156	C1orf162	-0.86	0.01
157	C1orf174	-0.51	0.01
158	C1orf216	-0.47	0.00
159	C1orf35	-0.86	0.00
160	C1QTNF5	-1.25	0.00
161	C22orf46	-0.52	0.00
162	C2CD2L	-0.61	0.00
163	C3orf18	-1.34	0.00
164	C4B	-1.84	0.01
165	C4orf3	-0.60	0.00
166	C5orf38	-1.54	0.00
167	C5orf66-AS1	-2.42	0.01
168	C6orf226	-1.23	0.00
169	C6orf47	-1.32	0.00
170	C6orf47	-1.32	0.00
171	C6orf47	-1.32	0.00
172	C7orf50	-1.03	0.00
173	C8orf34-AS1	0.60	0.00
174	C8orf58	-0.79	0.01
175	C9orf16	-2.14	0.00
176	C9orf78	-0.70	0.00
177	CA11	-1.93	0.00
178	CACNA1B	2.00	0.00
179	CACNA2D2	-1.51	0.00
180	CACNA2D4	-0.95	0.00
181	CADM3	-1.86	0.00
182	CALHM2	-0.86	0.00
183	CALM3	-0.67	0.00
184	CAMK1	-0.99	0.00
185	CAMKMT	0.52	0.00
186	CAPN12	-1.20	0.00
187	CAPNS1	-0.94	0.00
188	CARD16	-0.84	0.00
189	CARD19	-0.89	0.00
190	CAVIN1	-0.91	0.00
191	CBS	-1.46	0.01
192	CBWD6	0.73	0.01
193	CCDC103	-1.35	0.01
194	CCDC103	-1.19	0.01
195	CCDC107	-0.97	0.00
196	CCDC12	-0.76	0.00
197	CCDC124	-0.90	0.00

198	CCDC142	-0.50	0.01
199	CCDC159	-0.66	0.01
200	CCDC189	-1.27	0.00
201	CCDC24	-1.27	0.00
202	CCDC28B	-0.81	0.00
203	CCDC51	-0.97	0.00
204	CCDC71	-0.83	0.00
205	CCDC85A	-1.92	0.00
206	CCDC85B	-1.13	0.00
207	CCDC86	-0.77	0.00
208	CCDC89	-1.07	0.00
209	CCN2	1.09	0.01
210	CCN5	-1.95	0.00
211	CCN6	-2.45	0.00
212	CCNYL1	0.49	0.00
213	CCR10	-1.14	0.00
214	CCZ1B	0.73	0.00
215	CD2BP2-DT	-1.33	0.00
216	CD33	-0.81	0.01
217	CD3EAP	-0.81	0.00
218	CD68	-0.88	0.00
219	CD74	-1.92	0.00
220	CDC37	-0.90	0.00
221	CDH19	1.73	0.00
222	CDK11B	-0.62	0.00
223	CDK12	1.45	0.00
224	CDK2	-0.94	0.01
225	CDKN2AIP	-0.65	0.00
226	CDKN2AIPNL	-0.78	0.00
227	CDR1	-1.70	0.01
228	CDRT1	1.32	0.00
229	CDSN	-4.20	0.00
230	CERT1	0.42	0.00
231	CES1P1	-2.16	0.00
232	CES2	-0.69	0.00
233	CES3	1.84	0.00
234	CETP	-1.27	0.00
235	CFAP298	-0.96	0.00
236	CFB	-2.25	0.00
237	CH17-340M24.3	-1.03	0.01
238	CHCHD10	-1.04	0.00
239	CHCHD10	-1.04	0.00
240	CHCHD5	-1.42	0.00
241	CHMP2A	-0.71	0.00
242	CHRM5	0.71	0.00
243	CHRNB1	-0.62	0.00
244	CHRNB1	-0.62	0.00
245	CHRNB2	-0.90	0.01
246	CHST1	-1.57	0.01
247	CHST14	-0.76	0.00

248	CHST7	-0.73	0.01
249	CIART	-1.13	0.00
250	CIDEB	-1.23	0.00
251	CIDECP1	-0.84	0.00
252	CLDN10	2.61	0.00
253	CLEC11A	-1.51	0.00
254	CLPP	-1.11	0.00
255	CLTB	-0.81	0.00
256	CNOT2-DT	-0.75	0.01
257	CNOT6	0.58	0.01
258	CNPPD1	-1.01	0.00
259	CNPY3	-0.63	0.01
260	CNPY4	-0.56	0.01
261	COA3	-0.73	0.00
262	COA4	-1.14	0.00
263	COA6-AS1	-1.26	0.00
264	COL2A1	-2.27	0.00
265	COMMD4	-0.92	0.00
266	COMMD9	-0.76	0.01
267	COPS6	-0.57	0.00
268	COPS9	-1.03	0.00
269	CORO2B	-0.91	0.01
270	COX1	-1.39	0.00
271	COX14	-0.81	0.01
272	COX2	-1.65	0.00
273	COX4I2	-1.27	0.01
274	COX6A1	-0.95	0.00
275	COX6B1	-1.21	0.00
276	COX7A1	-1.48	0.00
277	CPLANE2	-0.96	0.00
278	CPTP	-1.18	0.00
279	CREB3L2-AS1	1.28	0.00
280	CRTAP	-0.62	0.00
281	CRTC2	-0.51	0.01
282	CSF1R	-0.75	0.00
283	CSF3R	-1.15	0.00
284	CST3	-1.59	0.00
285	CST7	-1.03	0.01
286	CTDSP1	-0.90	0.00
287	CTDSP2	-0.72	0.00
288	CTF1	-0.96	0.01
289	CTSF	-1.24	0.00
290	CUEDC2	-0.69	0.01
291	CUL3	0.38	0.00
292	CUTA	-1.13	0.00
293	CUTA	-1.10	0.00
294	CX3CR1	-1.53	0.00
295	CYB561D2	-0.87	0.00
296	CYB5D1	-0.77	0.00
297	CYP2B7P	-4.14	0.00

298	CYP2T1P	-1.42	0.00
299	CYP46A1	0.54	0.00
300	CYTB	-1.36	0.00
301	CYYR1-AS1	0.94	0.00
302	CZIB	-0.65	0.00
303	DBET	1.49	0.00
304	DBNDD2	-1.54	0.00
305	DCAF4L1	0.63	0.01
306	DCAKD	-0.73	0.00
307	DCDC2	-2.67	0.00
308	DCLRE1B	-0.75	0.00
309	DCTN3	-0.93	0.00
310	DCUN1D2	0.74	0.00
311	DDAH2	-1.31	0.00
312	DDAH2	-1.08	0.00
313	DDAH2	-1.32	0.00
314	DDAH2	-1.32	0.00
315	DDN-AS1	-0.93	0.00
316	DDRGK1	-0.84	0.00
317	DDT	-1.28	0.00
318	DDT	-1.48	0.00
319	DDX24	-0.65	0.00
320	DDX27	-0.87	0.00
321	DDX41	-0.64	0.00
322	DDX56	-0.54	0.01
323	DENND1C	-0.82	0.01
324	DENND2D	-0.72	0.00
325	DET1	-0.76	0.00
326	DFFA	-0.42	0.00
327	DGCR6	-0.88	0.00
328	DGCR6L	-1.06	0.00
329	DHPS	-0.60	0.00
330	DHRS4-AS1	-0.62	0.00
331	DHX36	0.74	0.00
332	DIAPH1-AS1	0.82	0.00
333	DLG1	0.60	0.00
334	DLGAP1-AS5	1.96	0.00
335	DMAC2	-0.57	0.00
336	DMAP1	-0.57	0.00
337	DMKN	-2.04	0.00
338	DMXL1	0.53	0.00
339	DNA2	0.64	0.01
340	DNAJB1	-0.62	0.00
341	DNAJB2	-0.61	0.00
342	DNAJC14	-0.49	0.01
343	DNAJC27-AS1	-0.82	0.01
344	DNAJC4	-1.00	0.00
345	DNAJC9-AS1	-0.89	0.00
346	DNLZ	-1.01	0.00
347	DNPH1	-1.01	0.01

348	DNTTIP1	-0.60	0.01
349	DOCK5	0.52	0.00
350	DOK1	-1.28	0.00
351	DPM2	-1.46	0.00
352	DPM3	-1.28	0.00
353	DPT	-1.20	0.01
354	DPY19L1	0.80	0.01
355	DRAM1	0.41	0.00
356	DRAP1	-0.94	0.00
357	DSCAM-AS1	-2.13	0.01
358	DSCR9	-0.90	0.00
359	DTX3	-1.11	0.00
360	DUSP28	-0.54	0.01
361	DUSP7	-0.90	0.01
362	DVL3	-0.65	0.00
363	DXO	-1.46	0.00
364	DXO	-1.46	0.00
365	DXO	-1.46	0.00
366	DYNLRB1	-0.73	0.00
367	DYRK4	-0.61	0.00
368	ECE2	-1.23	0.00
369	ECSCR	-1.19	0.00
370	EDF1	-1.02	0.00
371	EED	0.37	0.00
372	EFCAB3	0.45	0.00
373	EFEMP2	-1.14	0.00
374	EFHB	-0.90	0.01
375	EFHC1	0.51	0.01
376	EGFL8	-2.14	0.00
377	EGFL8	-2.14	0.00
378	EGFL8	-2.05	0.00
379	EGLN2	-1.18	0.00
380	EHMT1	0.65	0.01
381	EID1	-0.53	0.01
382	EIF1AD	-0.68	0.00
383	EIF2B2	-0.54	0.01
384	EIF2B4	-0.52	0.00
385	EIF3F	-0.65	0.00
386	EIF3F	-0.65	0.00
387	EIF3G	-0.74	0.00
388	EIF3I	-0.76	0.00
389	EIF4EBP1	-1.90	0.00
390	EIF4EBP3	-1.39	0.00
391	EIF5AL1	0.63	0.00
392	ELMOD1	1.47	0.00
393	ELOA	-0.62	0.00
394	ELOF1	-0.89	0.00
395	ELOVL1	-1.62	0.00
396	ELOVL2	-1.92	0.01
397	ELP5	-0.49	0.00

398	EMC10	-0.94	0.00
399	EMC6	-1.41	0.01
400	EML6	0.79	0.00
401	ENHO	-1.98	0.00
402	ENKD1	-0.68	0.01
403	ENTPD3-AS1	-0.74	0.00
404	EP300	0.73	0.00
405	EPHA5	2.90	0.00
406	ERCC1	-0.76	0.00
407	ERP29	-0.75	0.00
408	ESAM	-1.21	0.00
409	ESS2	-0.73	0.00
410	ETNK1	0.60	0.00
411	EWSR1	-0.61	0.00
412	FAAP20	-0.78	0.01
413	FAM117A	-0.87	0.00
414	FAM120AOS	-0.61	0.01
415	FAM122B	0.60	0.00
416	FAM167B	-1.17	0.01
417	FAM174C	-1.18	0.00
418	FAM184B	-1.54	0.00
419	FAM200B	-0.60	0.00
420	FAM32A	-0.66	0.00
421	FAM3D	2.28	0.00
422	FAM41C	1.26	0.00
423	FAM50A	-0.62	0.01
424	FAM71E1	-1.60	0.00
425	FAM83A	2.06	0.00
426	FAM86C1	-0.76	0.00
427	FAM89B	-0.98	0.00
428	FAM98B	0.62	0.00
429	FANCL	0.52	0.01
430	FARSA	-0.77	0.00
431	FASTKD1	0.53	0.00
432	FAU	-1.31	0.00
433	FBXL13	1.38	0.00
434	FBXL22	-0.92	0.00
435	FBXO11	0.51	0.00
436	FBXO15	0.66	0.00
437	FBXO2	-1.42	0.01
438	FCER1G	-1.21	0.00
439	FCHSD1	-0.48	0.00
440	FCN1	-0.94	0.00
441	FDCSP	3.40	0.00
442	FENDRR	0.99	0.00
443	FEZ1	-0.82	0.00
444	FGFBP3	-1.05	0.00
445	FHL2	-1.91	0.00
446	FHL3	-0.93	0.00
447	FIZ1	-0.74	0.00

448	FJX1	-1.15	0.00
449	FKBP2	-1.00	0.00
450	FKBP8	-0.66	0.01
451	FLII	-0.59	0.00
452	FLII	-0.59	0.00
453	FLJ20021	-1.06	0.00
454	FLJ36000	0.95	0.01
455	FLJ37453	-0.66	0.00
456	FLOT1	-0.63	0.00
457	FLOT1	-0.63	0.00
458	FLOT1	-0.63	0.00
459	FLOT1	-1.14	0.01
460	FLOT1	-0.65	0.00
461	FLOT1	-0.64	0.00
462	FLT3LG	-0.73	0.00
463	FMNL2	0.66	0.00
464	FOCAD	0.57	0.01
465	FOLR2	-1.23	0.00
466	FOXB1	1.49	0.00
467	FOXH1	-2.14	0.00
468	FOXJ3	0.56	0.01
469	FOXN3-AS1	-1.21	0.00
470	FOXO4	-0.83	0.00
471	FOXS1	-1.07	0.00
472	FSIP1	2.23	0.00
473	FTL	-1.13	0.00
474	FUNDC2	-0.57	0.00
475	FUZ	-1.11	0.00
476	FXYD5	-1.13	0.00
477	G2E3	0.75	0.00
478	G6PC3	-1.59	0.00
479	GABARAP	-1.02	0.00
480	GADD45A	-1.01	0.01
481	GADD45G	-1.18	0.00
482	GADD45GIP1	-1.38	0.00
483	GALT	-0.79	0.01
484	GAMT	-1.06	0.00
485	GAREM1	0.87	0.00
486	GATAD2A	-0.57	0.00
487	GBP2	-0.87	0.00
488	GBP3	-0.63	0.00
489	GCSAML	0.65	0.00
490	GDI1	-0.77	0.00
491	GET3	-1.04	0.00
492	GFER	-0.69	0.01
493	GFM1	0.58	0.01
494	GGT5	-0.92	0.00
495	GGT6	-1.13	0.00
496	GGTA1P	-0.92	0.00
497	GIMAP1	-0.97	0.00

498	GJA4	-1.21	0.00
499	GLMP	-0.68	0.00
500	GLT8D1	-0.46	0.00
501	GLYATL2	2.65	0.00
502	GMCL1	0.58	0.00
503	GMDS	0.63	0.01
504	GMPR2	-0.61	0.00
505	GNPTAB	0.63	0.00
506	GNPTG	-0.44	0.01
507	GOLGA8S	1.19	0.01
508	GOLM1	-0.72	0.00
509	GON7	-0.67	0.00
510	GON7	-0.67	0.00
511	GORASP1	-0.76	0.00
512	GPBAR1	-1.17	0.01
513	GPIHBP1	-1.96	0.00
514	GPKOW	-0.67	0.00
515	GPN2	-1.13	0.00
516	GPR108	-0.54	0.01
517	GPR143	-1.62	0.00
518	GPR151	-1.22	0.00
519	GPR21	0.47	0.00
520	GPR22	1.04	0.00
521	GPR22	0.96	0.00
522	GPR55	-1.74	0.00
523	GPR61	-0.96	0.00
524	GPS2	-0.74	0.00
525	GPSM3	-2.51	0.00
526	GPSM3	-2.51	0.00
527	GPSM3	-2.51	0.00
528	GPSM3	-2.51	0.00
529	GPSM3	-2.51	0.00
530	GPSM3	-2.51	0.00
531	GPX4	-0.77	0.01
532	GRAMD1C	0.91	0.00
533	GRHL3	-1.57	0.01
534	GRIA2	-1.84	0.00
535	GRK5	-0.98	0.00
536	GRN	-0.77	0.01
537	GS1-124K5.4	-1.04	0.01
538	GSDMD	-0.79	0.00
539	GSDMD	-0.79	0.00
540	GSTM4	-1.08	0.00
541	GTF2F1	-0.56	0.01
542	GTF2I	0.59	0.00
543	GTF3C5	-0.76	0.01
544	GTPBP6	-0.84	0.00
545	H1-0	-0.98	0.01
546	H19	-1.16	0.00
547	HARS1	-0.42	0.00

548	HAUS5	-0.88	0.00
549	HBA1	-2.15	0.00
550	HBA2	-2.78	0.00
551	HCFC1R1	-1.73	0.00
552	HCST	-1.42	0.00
553	HDAC11	-1.27	0.00
554	HDAC11-AS1	-2.19	0.00
555	HDAC4-AS1	-1.49	0.00
556	HDAC6	-0.40	0.00
557	HDAC7	-0.51	0.00
558	HDDC3	-1.04	0.00
559	HEATR5B	0.44	0.00
560	HECTD1	0.64	0.01
561	HEMK1	-0.72	0.00
562	HEXA-AS1	-0.74	0.00
563	HEXIM1	-0.91	0.00
564	HEXIM2	-0.89	0.00
565	HHEX	-1.09	0.00
566	HIBCH	0.56	0.00
567	HIGD1B	-1.15	0.00
568	HIGD2A	-1.26	0.00
569	HINT2	-1.24	0.00
570	HIPK1-AS1	-0.99	0.00
571	HIRIP3	-0.58	0.00
572	HLA-A	-1.91	0.01
573	HLA-A	-2.23	0.01
574	HLA-A	-2.23	0.01
575	HLA-A	-3.41	0.00
576	HLA-C	-3.53	0.00
577	HLA-DMA	-1.28	0.00
578	HLA-DMA	-1.28	0.00
579	HLA-DMA	-1.27	0.00
580	HLA-DMA	-1.27	0.00
581	HLA-DMA	-1.28	0.00
582	HLA-DPA1	-1.83	0.01
583	HLA-DPA1	-1.82	0.00
584	HLA-DPA1	-1.73	0.00
585	HLA-DPA1	-1.83	0.01
586	HLA-DPB2	0.87	0.00
587	HLA-DPB2	0.87	0.00
588	HLA-DQA1	-10.62	0.00
589	HLA-DRA	-4.64	0.00
590	HLA-DRB1	-7.02	0.00
591	HLA-L	-1.69	0.01
592	HNF1B	1.36	0.00
593	HOXA10-AS	-1.87	0.00
594	HOXA7	-1.48	0.01
595	HOXA9	-1.35	0.00
596	HOXA-AS3	-2.19	0.00
597	HSD17B8	-1.02	0.01

598	HSD17B8	-1.02	0.01
599	HSD17B8	-1.02	0.01
600	HSD17B8	-1.02	0.01
601	HSD17B8	-1.02	0.01
602	HSD17B8	-1.02	0.01
603	HSPB8	-2.31	0.00
604	HSPBP1	-0.80	0.00
605	HTRA2	-0.62	0.00
606	HVCN1	-0.60	0.00
607	HYAL3	-1.01	0.00
608	HYLS1	-0.56	0.01
609	IBA57-DT	-1.25	0.00
610	IFI27L2	-1.54	0.00
611	IFI27L2	-1.54	0.00
612	IFITM1	-1.43	0.00
613	IFITM2	-2.21	0.00
614	IFITM3	-1.65	0.00
615	IGBP1	-0.47	0.01
616	IGFBP6	-1.44	0.00
617	IGFBP7	-0.88	0.00
618	IGSF21	-1.55	0.00
619	IL20	-2.75	0.00
620	ILF3-DT	-0.83	0.00
621	IMP4	-0.68	0.00
622	IMPDH2	-0.87	0.00
623	INAFM1	-1.76	0.00
624	INCA1	-1.03	0.00
625	INKA1	-0.90	0.00
626	INO80	0.44	0.00
627	INO80B	-0.90	0.00
628	INSM1	-2.62	0.01
629	INTS11	-0.73	0.00
630	IPO13	-0.74	0.00
631	IQCC	-0.73	0.01
632	IQCF3	0.67	0.00
633	IREB2	0.44	0.00
634	IRF2BP1	-0.96	0.00
635	IRF3	-0.68	0.00
636	IRS1	-1.10	0.01
637	IRX1	-2.05	0.00
638	ISCA2	-0.56	0.01
639	ISCU	-0.66	0.00
640	ISG20L2	-0.46	0.00
641	ISLR	-1.04	0.00
642	ISOC2	-0.86	0.01
643	ITGA3	-1.48	0.00
644	ITPK1	-0.67	0.00
645	ITPK1	-0.67	0.00
646	JADE2	-0.86	0.01
647	JMJD8	-0.90	0.00

648	JOSD2	-1.21	0.00
649	KAAG1	-3.32	0.00
650	KANK2	-0.57	0.01
651	KANSL1L	0.60	0.01
652	KAT5	-0.63	0.00
653	KCNF1	-4.32	0.00
654	KCNH2	-2.05	0.00
655	KCNIP4-IT1	0.61	0.00
656	KCNJ8	-0.73	0.00
657	KCNK15	-1.97	0.00
658	KCNT1	1.86	0.00
659	KCTD11	-0.89	0.00
660	KCTD2	-0.68	0.00
661	KCTD21	-0.52	0.00
662	KCTD4	0.88	0.00
663	KDF1	-0.82	0.00
664	KEAP1	-0.63	0.00
665	KIF12	-2.32	0.00
666	KIF1B	0.50	0.00
667	KL	-1.33	0.00
668	KLHDC4	0.54	0.00
669	KRI1	-0.75	0.00
670	KRTCAP2	-1.00	0.00
671	KXD1	-0.57	0.00
672	LACTB2-AS1	0.59	0.00
673	LAMB2	-1.31	0.00
674	LAMTOR1	-0.69	0.00
675	LAMTOR2	-0.90	0.00
676	LAMTOR4	-1.06	0.00
677	LAMTOR5	-0.74	0.00
678	LAS1L	-0.59	0.00
679	LCMT2	-0.97	0.00
680	LDB1	-0.85	0.00
681	LDLRAP1	-0.84	0.00
682	LEAP2	-0.78	0.00
683	LENG1	-1.57	0.00
684	LHFPL3-AS2	0.74	0.01
685	LHFPL6	-0.74	0.00
686	LIMD2	-1.20	0.00
687	LIMS1-AS1	0.66	0.01
688	LINC00216	0.73	0.00
689	LINC00271	0.68	0.00
690	LINC00276	0.47	0.01
691	LINC00298	0.99	0.00
692	LINC00324	-1.59	0.00
693	LINC00461	1.36	0.00
694	LINC00536	-1.43	0.01
695	LINC00566	0.87	0.00
696	LINC00635	0.45	0.00
697	LINC00667	-0.52	0.00

698	LINC00836	0.73	0.00
699	LINC00907	0.77	0.00
700	LINC01016	-1.57	0.00
701	LINC01023	-1.74	0.00
702	LINC01124	-1.79	0.01
703	LINC01137	-0.65	0.00
704	LINC01144	-1.26	0.00
705	LINC01180	0.69	0.01
706	LINC01209	1.49	0.01
707	LINC01427	0.62	0.01
708	LINC01586	0.68	0.01
709	LINC01609	0.65	0.00
710	LINC01684	0.53	0.01
711	LINC01697	0.67	0.00
712	LINC01722	0.60	0.00
713	LINC01748	0.46	0.01
714	LINC01776	0.59	0.00
715	LINC01793	0.98	0.00
716	LINC01852	-0.80	0.00
717	LINC01864	0.60	0.01
718	LINC02055	2.05	0.00
719	LINC02225	0.42	0.00
720	LINC02261	0.85	0.00
721	LINC02274	0.61	0.00
722	LINC02275	0.52	0.00
723	LINC02361	-1.14	0.00
724	LINC02728	0.68	0.00
725	LINC02731	-0.90	0.00
726	LIX1L-AS1	-0.55	0.00
727	LMBR1	0.87	0.00
728	LMO2	-0.63	0.00
729	LOC100129484	-0.78	0.01
730	LOC100131315	1.75	0.00
731	LOC100287042	-0.96	0.00
732	LOC100287837	-1.63	0.00
733	LOC100289361	-1.63	0.00
734	LOC100505915	0.73	0.00
735	LOC100506289	-2.27	0.00
736	LOC100506688	0.69	0.00
737	LOC100507564	-1.31	0.00
738	LOC100507642	-1.98	0.00
739	LOC100996419	-0.93	0.01
740	LOC100996842	-1.19	0.00
741	LOC101927051	-1.14	0.00
742	LOC101927124	0.88	0.00
743	LOC101927136	0.60	0.00
744	LOC101927151	-0.58	0.00
745	LOC101927179	0.93	0.00
746	LOC101927420	-1.33	0.00
747	LOC101927752	-0.68	0.00

748	LOC101927974	-0.84	0.00
749	LOC101928069	-0.81	0.00
750	LOC101928336	0.49	0.01
751	LOC101928696	1.42	0.00
752	LOC101928728	-1.67	0.00
753	LOC101928731	0.88	0.00
754	LOC101928896	0.72	0.00
755	LOC101929054	-1.54	0.00
756	LOC101929243	-1.95	0.00
757	LOC101929331	-1.50	0.00
758	LOC101929709	0.80	0.00
759	LOC102546299	0.61	0.00
760	LOC102723407	-3.14	0.00
761	LOC102724889	-0.85	0.00
762	LOC105274304	-1.04	0.00
763	LOC105369306	0.59	0.00
764	LOC105369779	-1.07	0.00
765	LOC105370473	0.58	0.00
766	LOC105370792	-1.05	0.01
767	LOC105371414	0.65	0.00
768	LOC105371795	-1.72	0.00
769	LOC105372069	0.93	0.00
770	LOC105373347	0.65	0.01
771	LOC105373383	-1.45	0.00
772	LOC107984660	-1.00	0.01
773	LOC107985433	1.21	0.01
774	LOC108783654	-1.13	0.00
775	LOC112267983	-0.73	0.01
776	LOC153684	-0.64	0.00
777	LOC642361	-0.88	0.00
778	LOC644656	-0.87	0.00
779	LOC648987	-0.53	0.00
780	LOC651337	0.68	0.00
781	LOC730098	-1.71	0.00
782	LOC731157	-1.48	0.00
783	LOXL3	-0.71	0.01
784	LRCH3	0.67	0.00
785	LRRC32	-0.79	0.01
786	LRRC37A3	0.84	0.00
787	LRRC47	-0.62	0.00
788	LRRC69	0.77	0.00
789	LRRN2	-1.42	0.00
790	LRRTM2	1.01	0.00
791	LSM4	-0.85	0.00
792	LSM6	-0.46	0.00
793	LSM7	-1.12	0.00
794	LSMEM1	1.23	0.00
795	LSP1	-0.79	0.00
796	LSS	1.05	0.00
797	LTB	-1.13	0.00

798	LTN1	0.43	0.00
799	LUC7L	0.59	0.00
800	LYPD6	-1.41	0.01
801	LZTS2	-0.76	0.00
802	MAEL	-2.15	0.00
803	MAFG-DT	-1.60	0.00
804	MAGEA1	4.13	0.00
805	MAP1A	-0.63	0.00
806	MAP1LC3A	-0.93	0.00
807	MAP3K12	-0.99	0.00
808	MAPK3	-0.94	0.00
809	MAPKAPK5-AS1	-0.52	0.00
810	MAX	-0.62	0.00
811	MBD3	-0.93	0.00
812	MBOAT7	-1.37	0.01
813	MCM3	-0.75	0.01
814	MCRIP1	-0.88	0.01
815	MEA1	-1.08	0.00
816	MED11	-0.90	0.00
817	MED14	0.31	0.00
818	MED18	-0.66	0.00
819	MED19	-1.08	0.00
820	MED22	-0.45	0.01
821	MED22	-0.45	0.01
822	MED29	-0.62	0.01
823	MEIS3	-0.90	0.00
824	MEOX1	-1.67	0.00
825	MEPCE	-0.81	0.00
826	MESD	-0.42	0.01
827	METTL1	-0.89	0.00
828	MFAP4	-1.44	0.00
829	MFN1	0.58	0.00
830	MGC16275	-0.81	0.00
831	MICB	-1.45	0.01
832	MICB	-1.45	0.01
833	MICOS13	-1.41	0.00
834	MIF4GD	-0.95	0.00
835	MINCR	-1.70	0.00
836	MIR1299	1.14	0.00
837	MIR133A1HG	0.90	0.00
838	MIR199A2	-1.79	0.00
839	MIR200CHG	-1.46	0.00
840	MIR27B	-1.36	0.00
841	MIR3150BHG	1.88	0.00
842	MIR3648-1	0.78	0.00
843	MIR3648-2	0.78	0.00
844	MIR4458HG	-0.80	0.01
845	MIR4742	1.09	0.00
846	MIR4755	1.17	0.00
847	MIR497HG	-1.48	0.00

848	MIR548W	0.74	0.01
849	MIR644A	1.28	0.00
850	MIR663B	0.92	0.00
851	MIR941-1	1.31	0.00
852	MLLT11	-0.79	0.00
853	MLST8	-0.66	0.00
854	MMACHC	-0.91	0.00
855	MMADHC-DT	-0.85	0.00
856	MMP28	-1.13	0.00
857	MOAP1	-0.79	0.00
858	MOAP1	-0.79	0.00
859	MOB1B	0.44	0.01
860	MOB3C	-0.94	0.00
861	MON1A	-0.77	0.00
862	MON2	0.70	0.01
863	MORC3	0.43	0.00
864	MORN3	-1.03	0.00
865	MPI	-0.95	0.00
866	MPRIP	-0.52	0.01
867	MPV17	-0.61	0.00
868	MRM2	-0.97	0.00
869	MRPL10	-1.29	0.00
870	MRPL16	-0.59	0.00
871	MRPL17	-0.95	0.00
872	MRPL20	-0.69	0.00
873	MRPL20-AS1	-0.87	0.00
874	MRPL24	-0.83	0.00
875	MRPL27	-0.97	0.00
876	MRPL34	-0.83	0.00
877	MRPL43	-0.68	0.01
878	MRPL49	-0.73	0.00
879	MRPL53	-1.12	0.00
880	MRPL55	-0.99	0.00
881	MRPL57	-0.80	0.00
882	MRPS18B	-0.52	0.01
883	MRPS18B	-0.52	0.01
884	MRPS18B	-0.52	0.01
885	MRPS18B	-0.52	0.01
886	MRPS18B	-0.52	0.01
887	MRPS18B	-0.52	0.01
888	MRPS2	-0.98	0.00
889	MRPS21	-0.62	0.00
890	MRPS26	-0.92	0.00
891	MRPS34	-0.74	0.01
892	MRTO4	-0.63	0.00
893	MSH5	-0.86	0.00
894	MSL1	-0.99	0.01
895	MST1	-1.18	0.00
896	MT1L	-1.99	0.00
897	MT2A	-1.39	0.00

898	MTCP1	-0.98	0.00
899	MTFMT	-0.50	0.01
900	MTFP1	-1.14	0.01
901	MUC6	-1.70	0.00
902	MUSTN1	-2.53	0.00
903	MUTYH	-0.50	0.01
904	MVB12A	-1.12	0.00
905	MXD4	-0.58	0.01
906	MYL5	-1.26	0.00
907	MYL6B	-1.18	0.00
908	MYL9	-1.08	0.00
909	MYLK3	0.39	0.01
910	MYO1D	0.63	0.00
911	MYSM1	0.63	0.01
912	MZF1-AS1	-0.90	0.00
913	ENSG00000116652	0.84	0.01
914	ENSG00000139239	-0.95	0.00
915	ENSG00000168852	0.82	0.01
916	ENSG00000174403	0.91	0.00
917	ENSG00000176134	1.30	0.00
918	ENSG00000176320	0.66	0.01
919	ENSG00000176933	-1.43	0.00
920	ENSG00000178636	0.53	0.01
921	ENSG00000179899	-0.92	0.00
922	ENSG00000179979	0.57	0.00
923	ENSG00000182021	1.03	0.00
924	ENSG00000189149	0.51	0.01
925	ENSG00000197332	0.64	0.00
926	ENSG00000197376	1.11	0.00
927	ENSG00000200314	1.25	0.00
928	ENSG00000200525	1.50	0.00
929	ENSG00000200882	0.93	0.00
930	ENSG00000201435	1.03	0.00
931	ENSG00000202374	0.99	0.00
932	ENSG00000202444	1.98	0.01
933	ENSG00000204850	-1.28	0.01
934	ENSG00000205176	2.00	0.00
935	ENSG00000205583	0.64	0.00
936	ENSG00000205622	0.76	0.00
937	ENSG00000206739	0.94	0.01
938	ENSG00000209082	1.57	0.00
939	ENSG00000210117	2.31	0.00
940	ENSG00000210184	1.70	0.00
941	ENSG00000210194	1.50	0.00
942	ENSG00000210195	1.51	0.00
943	ENSG00000211640	-2.41	0.00
944	ENSG00000211645	-2.47	0.00
945	ENSG00000211941	-2.04	0.00
946	ENSG00000211955	-3.19	0.00
947	ENSG00000212342	0.99	0.01

948	ENSG00000213846	-0.82	0.00
949	ENSG00000214182	-1.23	0.00
950	ENSG00000214283	0.88	0.00
951	ENSG00000214389	0.88	0.00
952	ENSG00000214559	0.64	0.01
953	ENSG00000216613	1.29	0.00
954	ENSG00000217624	0.94	0.01
955	ENSG00000217767	1.20	0.00
956	ENSG00000217783	0.84	0.00
957	ENSG00000218283	0.75	0.00
958	ENSG00000218996	1.37	0.00
959	ENSG00000219085	0.76	0.00
960	ENSG00000219409	0.96	0.00
961	ENSG00000220392	0.94	0.00
962	ENSG00000221439	1.10	0.01
963	ENSG00000223505	0.87	0.01
964	ENSG00000223525	0.72	0.00
965	ENSG00000223653	0.67	0.01
966	ENSG00000223704	0.51	0.01
967	ENSG00000223911	0.75	0.01
968	ENSG00000224007	0.90	0.00
969	ENSG00000224078	0.82	0.00
970	ENSG00000224114	0.70	0.01
971	ENSG00000224117	1.79	0.00
972	ENSG00000224152	-0.84	0.00
973	ENSG00000224358	-0.50	0.01
974	ENSG00000224418	-1.10	0.00
975	ENSG00000224885	0.78	0.00
976	ENSG00000224905	0.75	0.00
977	ENSG00000224992	-1.03	0.01
978	ENSG00000225031	-3.97	0.00
979	ENSG00000225078	1.51	0.00
980	ENSG00000225224	0.93	0.00
981	ENSG00000225226	0.59	0.01
982	ENSG00000225243	1.13	0.01
983	ENSG00000225300	0.55	0.01
984	ENSG00000225401	-0.92	0.00
985	ENSG00000225761	1.05	0.01
986	ENSG00000225767	0.74	0.00
987	ENSG00000225806	-1.22	0.00
988	ENSG00000225840	-4.70	0.00
989	ENSG00000225945	-1.16	0.00
990	ENSG00000226010	1.05	0.01
991	ENSG00000226200	-0.93	0.00
992	ENSG00000226318	0.80	0.01
993	ENSG00000226647	0.65	0.01
994	ENSG00000226801	1.45	0.00
995	ENSG00000226899	0.67	0.00
996	ENSG00000226982	0.98	0.00
997	ENSG00000227197	1.17	0.00

998	ENSG00000227227	0.79	0.00
999	ENSG00000227267	1.24	0.01
1000	ENSG00000227583	1.23	0.00
1001	ENSG00000227606	1.24	0.00
1002	ENSG00000227716	0.68	0.01
1003	ENSG00000227896	-0.67	0.00
1004	ENSG00000227945	1.05	0.00
1005	ENSG00000227946	-0.86	0.00
1006	ENSG00000228172	-0.89	0.00
1007	ENSG00000228343	-0.74	0.00
1008	ENSG00000228513	1.23	0.00
1009	ENSG00000228540	1.10	0.00
1010	ENSG00000228573	0.86	0.00
1011	ENSG00000228728	0.89	0.00
1012	ENSG00000229025	0.67	0.01
1013	ENSG00000229048	0.96	0.00
1014	ENSG00000229097	0.72	0.00
1015	ENSG00000229273	-1.51	0.00
1016	ENSG00000229320	-0.79	0.00
1017	ENSG00000229325	0.93	0.00
1018	ENSG00000229431	-0.67	0.01
1019	ENSG00000229502	0.72	0.00
1020	ENSG00000229728	0.89	0.00
1021	ENSG00000229800	0.83	0.00
1022	ENSG00000229816	0.67	0.00
1023	ENSG00000229839	1.06	0.00
1024	ENSG00000230022	0.95	0.00
1025	ENSG00000230046	0.86	0.00
1026	ENSG00000230131	1.27	0.01
1027	ENSG00000230433	1.00	0.01
1028	ENSG00000230439	-1.64	0.00
1029	ENSG00000230516	0.52	0.00
1030	ENSG00000230551	0.66	0.00
1031	ENSG00000230572	1.09	0.00
1032	ENSG00000230585	0.60	0.00
1033	ENSG00000230612	0.43	0.01
1034	ENSG00000230696	0.85	0.00
1035	ENSG00000230715	-1.35	0.01
1036	ENSG00000230735	0.84	0.00
1037	ENSG00000230894	0.64	0.00
1038	ENSG00000231049	1.56	0.00
1039	ENSG00000231050	-1.40	0.00
1040	ENSG00000231310	0.75	0.00
1041	ENSG00000231588	-2.39	0.00
1042	ENSG00000231765	1.10	0.00
1043	ENSG00000231770	-0.63	0.01
1044	ENSG00000231842	1.29	0.00
1045	ENSG00000231952	0.85	0.00
1046	ENSG00000232006	0.51	0.00
1047	ENSG00000232216	-3.07	0.00

1048	ENSG00000232504	-1.14	0.00
1049	ENSG00000232542	0.78	0.00
1050	ENSG00000232554	1.07	0.00
1051	ENSG00000232573	-7.11	0.00
1052	ENSG00000232754	0.90	0.01
1053	ENSG00000232934	0.59	0.00
1054	ENSG00000233044	0.74	0.00
1055	ENSG00000233061	0.60	0.01
1056	ENSG00000233072	1.05	0.00
1057	ENSG00000233461	-1.04	0.00
1058	ENSG00000233646	1.76	0.00
1059	ENSG00000233885	0.69	0.00
1060	ENSG00000233998	0.90	0.00
1061	ENSG00000234361	0.72	0.01
1062	ENSG00000234378	0.69	0.01
1063	ENSG00000234686	1.16	0.00
1064	ENSG00000234694	-1.19	0.00
1065	ENSG00000234814	1.25	0.00
1066	ENSG00000234818	0.98	0.00
1067	ENSG00000234864	0.61	0.01
1068	ENSG00000234917	-1.04	0.00
1069	ENSG00000234982	1.78	0.00
1070	ENSG00000234995	0.99	0.01
1071	ENSG00000235008	0.92	0.01
1072	ENSG00000235298	-0.81	0.00
1073	ENSG00000235725	0.79	0.00
1074	ENSG00000235833	0.96	0.00
1075	ENSG00000235869	1.48	0.00
1076	ENSG00000235978	-1.43	0.01
1077	ENSG00000236018	0.85	0.00
1078	ENSG00000236184	0.59	0.01
1079	ENSG00000236255	0.62	0.00
1080	ENSG00000236397	-1.89	0.00
1081	ENSG00000236452	-2.79	0.00
1082	ENSG00000236526	0.86	0.00
1083	ENSG00000236559	0.69	0.00
1084	ENSG00000236673	0.54	0.00
1085	ENSG00000236963	0.65	0.01
1086	ENSG00000237181	-1.16	0.00
1087	ENSG00000237276	-1.29	0.00
1088	ENSG00000237317	1.25	0.00
1089	ENSG00000237380	-0.86	0.01
1090	ENSG00000237406	0.81	0.00
1091	ENSG00000237414	0.67	0.00
1092	ENSG00000237442	-3.09	0.00
1093	ENSG00000237483	1.17	0.00
1094	ENSG00000237529	0.78	0.01
1095	ENSG00000237672	0.79	0.00
1096	ENSG00000237689	1.10	0.00
1097	ENSG00000237827	0.87	0.00

1098	ENSG00000237840	-0.71	0.00
1099	ENSG00000237950	-1.25	0.00
1100	ENSG00000237954	0.82	0.00
1101	ENSG00000238045	-0.58	0.00
1102	ENSG00000238058	-0.69	0.00
1103	ENSG00000238164	-1.19	0.00
1104	ENSG00000238279	-1.63	0.00
1105	ENSG00000238290	0.56	0.00
1106	ENSG00000239280	0.65	0.00
1107	ENSG00000239300	0.79	0.00
1108	ENSG00000239351	0.92	0.00
1109	ENSG00000239835	0.80	0.00
1110	ENSG00000240005	-1.40	0.00
1111	ENSG00000240074	1.11	0.00
1112	ENSG00000240132	0.66	0.01
1113	ENSG00000240342	-1.02	0.00
1114	ENSG00000241003	0.83	0.00
1115	ENSG00000241007	1.13	0.00
1116	ENSG00000241043	0.47	0.00
1117	ENSG00000241112	0.70	0.01
1118	ENSG00000241244	-2.22	0.01
1119	ENSG00000241280	2.20	0.01
1120	ENSG00000241294	-2.23	0.00
1121	ENSG00000241438	0.96	0.00
1122	ENSG00000241475	0.72	0.00
1123	ENSG00000241593	1.13	0.00
1124	ENSG00000241599	0.83	0.00
1125	ENSG00000242154	1.13	0.00
1126	ENSG00000243053	0.80	0.00
1127	ENSG00000243368	1.17	0.00
1128	ENSG00000243650	-0.62	0.01
1129	ENSG00000243797	0.68	0.01
1130	ENSG00000244124	0.81	0.00
1131	ENSG00000244239	-2.00	0.00
1132	ENSG00000244301	1.07	0.00
1133	ENSG00000244378	0.64	0.01
1134	ENSG00000244490	0.89	0.00
1135	ENSG00000244575	-2.07	0.00
1136	ENSG00000245025	-0.67	0.01
1137	ENSG00000246851	0.80	0.00
1138	ENSG00000247092	-0.74	0.00
1139	ENSG00000248240	-0.86	0.00
1140	ENSG00000248367	-1.25	0.00
1141	ENSG00000248458	0.55	0.01
1142	ENSG00000248489	-0.76	0.00
1143	ENSG00000248553	1.39	0.01
1144	ENSG00000249006	1.43	0.00
1145	ENSG00000249028	0.71	0.00
1146	ENSG00000249140	0.77	0.00
1147	ENSG00000249316	0.70	0.00

1148	ENSG00000249439	0.95	0.00
1149	ENSG00000249673	-0.61	0.00
1150	ENSG00000250017	0.44	0.01
1151	ENSG00000250039	0.75	0.00
1152	ENSG00000250222	-1.77	0.00
1153	ENSG00000250318	-1.02	0.00
1154	ENSG00000250371	1.04	0.01
1155	ENSG00000250448	0.98	0.00
1156	ENSG00000250509	0.67	0.00
1157	ENSG00000250848	0.85	0.00
1158	ENSG00000250853	1.02	0.00
1159	ENSG00000250869	0.62	0.00
1160	ENSG00000250979	1.31	0.00
1161	ENSG00000251054	1.02	0.01
1162	ENSG00000251079	0.71	0.00
1163	ENSG00000251379	0.62	0.00
1164	ENSG00000251388	0.96	0.01
1165	ENSG00000252172	1.32	0.01
1166	ENSG00000252269	1.28	0.00
1167	ENSG00000253153	1.14	0.00
1168	ENSG00000253167	0.68	0.00
1169	ENSG00000253376	0.50	0.00
1170	ENSG00000253503	0.76	0.00
1171	ENSG00000253669	-0.86	0.00
1172	ENSG00000253720	0.79	0.00
1173	ENSG00000253775	0.81	0.00
1174	ENSG00000253859	0.55	0.01
1175	ENSG00000253908	1.04	0.00
1176	ENSG00000254840	2.53	0.00
1177	ENSG00000254962	0.99	0.01
1178	ENSG00000255135	-0.69	0.00
1179	ENSG00000255197	-1.25	0.00
1180	ENSG00000255224	-0.98	0.00
1181	ENSG00000255321	0.74	0.00
1182	ENSG00000255328	0.73	0.00
1183	ENSG00000255968	0.79	0.00
1184	ENSG00000256056	0.81	0.01
1185	ENSG00000256092	-0.98	0.00
1186	ENSG00000256274	0.76	0.00
1187	ENSG00000256646	-0.53	0.01
1188	ENSG00000256667	0.56	0.00
1189	ENSG00000256955	0.62	0.00
1190	ENSG00000257151	1.13	0.00
1191	ENSG00000257277	0.51	0.01
1192	ENSG00000257298	0.60	0.01
1193	ENSG00000257398	0.78	0.00
1194	ENSG00000257557	-0.59	0.00
1195	ENSG00000257781	0.79	0.00
1196	ENSG00000257954	1.11	0.00
1197	ENSG00000258044	0.57	0.00

1198	ENSG00000258082	-1.08	0.00
1199	ENSG00000258178	1.09	0.00
1200	ENSG00000258504	-4.93	0.00
1201	ENSG00000258526	0.66	0.01
1202	ENSG00000258636	0.65	0.00
1203	ENSG00000258670	0.54	0.00
1204	ENSG00000258765	0.72	0.00
1205	ENSG00000258813	-1.08	0.00
1206	ENSG00000258858	-1.21	0.00
1207	ENSG00000258957	-0.87	0.00
1208	ENSG00000259090	0.95	0.00
1209	ENSG00000259094	1.80	0.00
1210	ENSG00000259113	0.97	0.01
1211	ENSG00000259165	0.76	0.00
1212	ENSG00000259209	1.12	0.00
1213	ENSG00000259271	-2.02	0.01
1214	ENSG00000259276	0.81	0.00
1215	ENSG00000259294	1.07	0.00
1216	ENSG00000259298	0.67	0.00
1217	ENSG00000259319	-1.38	0.00
1218	ENSG00000259408	0.90	0.00
1219	ENSG00000259505	0.79	0.01
1220	ENSG00000259521	0.99	0.01
1221	ENSG00000259616	0.62	0.01
1222	ENSG00000259627	-5.68	0.00
1223	ENSG00000259642	-0.74	0.00
1224	ENSG00000259828	0.80	0.00
1225	ENSG00000259866	0.77	0.00
1226	ENSG00000259877	-0.57	0.00
1227	ENSG00000259915	0.82	0.00
1228	ENSG00000259959	0.66	0.00
1229	ENSG00000260018	-1.60	0.01
1230	ENSG00000260022	1.36	0.01
1231	ENSG00000260025	-2.69	0.00
1232	ENSG00000260035	-2.41	0.00
1233	ENSG00000260060	-0.78	0.00
1234	ENSG00000260062	0.55	0.00
1235	ENSG00000260141	1.12	0.00
1236	ENSG00000260274	-1.33	0.00
1237	ENSG00000260350	1.23	0.00
1238	ENSG00000260455	1.34	0.01
1239	ENSG00000260505	0.94	0.00
1240	ENSG00000260526	-1.08	0.00
1241	ENSG00000260572	-1.07	0.00
1242	ENSG00000260669	-0.62	0.01
1243	ENSG00000260708	-1.36	0.00
1244	ENSG00000260772	-7.08	0.00
1245	ENSG00000260796	-0.72	0.00
1246	ENSG00000260923	0.80	0.00
1247	ENSG00000260924	-1.28	0.00

1248	ENSG00000260975	0.83	0.00
1249	ENSG00000260992	-1.68	0.00
1250	ENSG00000261019	0.82	0.01
1251	ENSG00000261186	-1.38	0.00
1252	ENSG00000261188	-1.24	0.00
1253	ENSG00000261200	0.94	0.00
1254	ENSG00000261220	-1.47	0.00
1255	ENSG00000261242	-1.37	0.00
1256	ENSG00000261293	0.85	0.01
1257	ENSG00000261296	1.10	0.00
1258	ENSG00000261334	-1.24	0.01
1259	ENSG00000261351	-0.64	0.00
1260	ENSG00000261360	-1.05	0.00
1261	ENSG00000261369	0.69	0.01
1262	ENSG00000261441	-1.94	0.00
1263	ENSG00000261462	1.03	0.01
1264	ENSG00000261490	-0.75	0.00
1265	ENSG00000261512	-1.19	0.00
1266	ENSG00000261528	0.60	0.01
1267	ENSG00000261529	0.80	0.00
1268	ENSG00000261574	0.99	0.00
1269	ENSG00000261582	-1.04	0.00
1270	ENSG00000261642	0.73	0.00
1271	ENSG00000261654	0.72	0.00
1272	ENSG00000261763	0.56	0.00
1273	ENSG00000261826	0.54	0.00
1274	ENSG00000261868	0.94	0.01
1275	ENSG00000262049	-1.22	0.00
1276	ENSG00000262529	0.95	0.01
1277	ENSG00000262888	0.81	0.01
1278	ENSG00000263083	0.76	0.00
1279	ENSG00000263105	-1.41	0.00
1280	ENSG00000263412	-0.97	0.00
1281	ENSG00000263477	0.85	0.00
1282	ENSG00000263826	-0.88	0.01
1283	ENSG00000263884	-0.65	0.01
1284	ENSG00000264151	0.59	0.00
1285	ENSG00000264270	0.70	0.00
1286	ENSG00000264707	0.59	0.01
1287	ENSG00000264727	0.86	0.00
1288	ENSG00000264772	-0.87	0.00
1289	ENSG00000265128	1.09	0.01
1290	ENSG00000265136	1.60	0.00
1291	ENSG00000265204	1.01	0.00
1292	ENSG00000265205	0.59	0.01
1293	ENSG00000265206	-1.42	0.00
1294	ENSG00000265451	0.93	0.01
1295	ENSG00000265713	0.91	0.00
1296	ENSG00000265908	0.69	0.00
1297	ENSG00000266208	-1.45	0.00

1298	ENSG00000266274	-1.04	0.00
1299	ENSG00000266579	1.03	0.00
1300	ENSG00000266696	1.05	0.00
1301	ENSG00000266985	0.54	0.01
1302	ENSG00000267006	0.89	0.00
1303	ENSG00000267009	0.57	0.00
1304	ENSG00000267239	0.77	0.00
1305	ENSG00000267285	0.85	0.01
1306	ENSG00000267457	0.92	0.00
1307	ENSG00000267462	0.53	0.00
1308	ENSG00000267469	-5.58	0.00
1309	ENSG00000267493	-1.15	0.00
1310	ENSG00000267560	0.69	0.01
1311	ENSG00000268058	0.99	0.00
1312	ENSG00000268163	1.17	0.00
1313	ENSG00000268218	0.61	0.00
1314	ENSG00000268366	-1.16	0.00
1315	ENSG00000268628	-0.85	0.00
1316	ENSG00000268736	1.32	0.01
1317	ENSG00000269176	-1.11	0.00
1318	ENSG00000269189	1.06	0.01
1319	ENSG00000269242	-2.20	0.00
1320	ENSG00000269243	-2.54	0.00
1321	ENSG00000269486	0.53	0.00
1322	ENSG00000269560	0.61	0.00
1323	ENSG00000269570	0.68	0.00
1324	ENSG00000269842	0.86	0.00
1325	ENSG00000269929	0.65	0.01
1326	ENSG00000269952	1.19	0.01
1327	ENSG00000269974	1.23	0.00
1328	ENSG00000270012	-0.65	0.00
1329	ENSG00000270099	1.10	0.00
1330	ENSG00000270112	1.36	0.00
1331	ENSG00000270116	0.74	0.00
1332	ENSG00000270175	-1.33	0.00
1333	ENSG00000270177	-1.53	0.00
1334	ENSG00000270269	1.11	0.00
1335	ENSG00000270554	1.08	0.00
1336	ENSG00000270585	0.69	0.01
1337	ENSG00000270640	1.19	0.01
1338	ENSG00000270832	1.14	0.00
1339	ENSG00000270906	1.23	0.00
1340	ENSG00000270964	-1.02	0.00
1341	ENSG00000271172	0.84	0.01
1342	ENSG00000271324	-1.51	0.00
1343	ENSG00000271533	0.76	0.00
1344	ENSG00000271643	-1.04	0.00
1345	ENSG00000271737	-1.09	0.00
1346	ENSG00000271780	-0.88	0.00
1347	ENSG00000271797	-1.22	0.00

1348	ENSG00000271806	0.64	0.00
1349	ENSG00000271918	-1.09	0.00
1350	ENSG00000271991	-1.57	0.00
1351	ENSG00000272054	0.47	0.00
1352	ENSG00000272092	-1.23	0.00
1353	ENSG00000272183	-0.97	0.01
1354	ENSG00000272277	-0.98	0.01
1355	ENSG00000272417	1.20	0.00
1356	ENSG00000272449	-1.47	0.00
1357	ENSG00000272472	1.00	0.01
1358	ENSG00000272486	0.81	0.00
1359	ENSG00000272525	-1.46	0.00
1360	ENSG00000272572	-0.97	0.00
1361	ENSG00000272604	0.62	0.00
1362	ENSG00000272654	-0.85	0.00
1363	ENSG00000272663	-1.57	0.00
1364	ENSG00000272667	-1.22	0.00
1365	ENSG00000272800	0.72	0.01
1366	ENSG00000272829	-2.10	0.00
1367	ENSG00000272831	-1.06	0.00
1368	ENSG00000272941	-0.70	0.00
1369	ENSG00000272953	-1.60	0.00
1370	ENSG00000272983	0.73	0.00
1371	ENSG00000273026	-1.03	0.00
1372	ENSG00000273080	-1.26	0.00
1373	ENSG00000273139	-1.11	0.00
1374	ENSG00000273148	-0.72	0.00
1375	ENSG00000273175	-1.54	0.00
1376	ENSG00000273275	1.04	0.00
1377	ENSG00000273365	0.82	0.01
1378	ENSG00000273399	0.92	0.01
1379	ENSG00000273420	-1.76	0.00
1380	ENSG00000273456	-1.79	0.00
1381	ENSG00000273702	-1.09	0.00
1382	ENSG00000274064	0.83	0.00
1383	ENSG00000274093	1.17	0.00
1384	ENSG00000274104	-1.06	0.00
1385	ENSG00000274162	0.91	0.00
1386	ENSG00000274281	0.57	0.00
1387	ENSG00000274364	0.86	0.01
1388	ENSG00000274497	-1.89	0.01
1389	ENSG00000274536	-0.97	0.00
1390	ENSG00000274615	1.32	0.00
1391	ENSG00000274627	0.48	0.01
1392	ENSG00000274776	0.84	0.00
1393	ENSG00000274827	0.99	0.01
1394	ENSG00000275029	1.52	0.00
1395	ENSG00000275197	0.85	0.01
1396	ENSG00000275413	0.68	0.00
1397	ENSG00000275426	1.16	0.00

1398	ENSG00000275441	-1.72	0.00
1399	ENSG00000275454	-1.23	0.00
1400	ENSG00000275488	0.77	0.00
1401	ENSG00000275494	-1.13	0.01
1402	ENSG00000275512	0.51	0.00
1403	ENSG00000276032	2.05	0.00
1404	ENSG00000276107	1.27	0.00
1405	ENSG00000276116	-1.29	0.00
1406	ENSG00000276223	-2.22	0.00
1407	ENSG00000276272	0.46	0.00
1408	ENSG00000276282	0.90	0.00
1409	ENSG00000276434	1.18	0.00
1410	ENSG00000276517	0.62	0.00
1411	ENSG00000276564	0.86	0.00
1412	ENSG00000276724	0.68	0.01
1413	ENSG00000276728	-1.25	0.00
1414	ENSG00000276744	-1.13	0.00
1415	ENSG00000276791	-0.97	0.00
1416	ENSG00000276809	0.74	0.00
1417	ENSG00000276849	-1.52	0.00
1418	ENSG00000277130	0.68	0.00
1419	ENSG00000277151	0.65	0.01
1420	ENSG00000277283	-0.88	0.00
1421	ENSG00000277297	0.75	0.00
1422	ENSG00000277369	-0.94	0.00
1423	ENSG00000277493	0.73	0.01
1424	ENSG00000277619	-2.86	0.00
1425	ENSG00000277687	0.70	0.01
1426	ENSG00000277767	0.39	0.01
1427	ENSG00000277782	-0.74	0.00
1428	ENSG00000277795	1.10	0.00
1429	ENSG00000277840	0.86	0.00
1430	ENSG00000278376	-1.18	0.00
1431	ENSG00000278716	1.26	0.01
1432	ENSG00000278834	-0.82	0.00
1433	ENSG00000278961	-3.22	0.00
1434	ENSG00000278996	1.67	0.00
1435	ENSG00000279217	0.47	0.00
1436	ENSG00000279342	0.72	0.00
1437	ENSG00000279375	1.41	0.01
1438	ENSG00000279623	1.06	0.00
1439	ENSG00000280114	1.84	0.00
1440	ENSG00000280169	0.70	0.00
1441	ENSG00000280243	1.32	0.01
1442	ENSG00000280341	0.59	0.01
1443	ENSG00000280441	1.67	0.00
1444	ENSG00000280612	1.05	0.00
1445	ENSG00000280614	-8.51	0.00
1446	ENSG00000280708	-0.97	0.01
1447	ENSG00000280770	0.90	0.00

1448	ENSG00000280800	-8.51	0.00
1449	ENSG00000281019	0.69	0.00
1450	ENSG00000281181	-8.51	0.00
1451	ENSG00000281197	-1.19	0.00
1452	ENSG00000281344	0.53	0.00
1453	ENSG00000281383	-8.65	0.00
1454	ENSG00000281501	0.42	0.01
1455	ENSG00000281617	1.20	0.00
1456	ENSG00000281734	-1.47	0.00
1457	ENSG00000281740	-1.50	0.00
1458	ENSG00000282024	0.93	0.01
1459	ENSG00000282075	-2.17	0.00
1460	ENSG00000282084	0.67	0.01
1461	ENSG00000282091	0.58	0.00
1462	ENSG00000282322	-2.04	0.00
1463	ENSG00000282442	1.23	0.00
1464	ENSG00000282461	-1.98	0.00
1465	ENSG00000282506	-1.77	0.00
1466	ENSG00000282555	1.75	0.00
1467	ENSG00000282666	-2.07	0.00
1468	ENSG00000282671	-2.23	0.00
1469	ENSG00000282695	1.52	0.00
1470	ENSG00000282827	-1.51	0.00
1471	ENSG00000282930	0.53	0.00
1472	ENSG00000283001	0.75	0.00
1473	ENSG00000283103	-0.77	0.00
1474	ENSG00000283106	-0.81	0.00
1475	ENSG00000283162	-2.62	0.00
1476	ENSG00000283192	-0.86	0.00
1477	ENSG00000283213	0.60	0.00
1478	ENSG00000283247	-2.34	0.00
1479	ENSG00000283735	0.48	0.00
1480	ENSG00000283861	0.53	0.01
1481	ENSG00000283905	0.53	0.00
1482	ENSG00000283907	-7.60	0.00
1483	ENSG00000284056	0.54	0.00
1484	ENSG00000284072	1.97	0.01
1485	ENSG00000284274	-0.93	0.01
1486	ENSG00000284461	-1.53	0.00
1487	ENSG00000284556	0.77	0.00
1488	ENSG00000284578	0.70	0.00
1489	NAA16	0.93	0.00
1490	NAA80	-1.05	0.00
1491	NAB2	-1.03	0.00
1492	NABP2	-0.99	0.00
1493	NAGS	-1.28	0.01
1494	NAPA	-0.65	0.01
1495	NAT14	-1.02	0.00
1496	NBDY	-0.69	0.00
1497	NBEAL1	0.52	0.01

1498	NBL1	-1.28	0.00
1499	NCKAP1	0.49	0.01
1500	NCKIPSD	-0.80	0.00
1501	NCOA5	-0.44	0.00
1502	ND4	-1.83	0.00
1503	ND4L	-1.69	0.00
1504	NDUFA3	-1.73	0.00
1505	NDUFA3	-1.73	0.00
1506	NDUFA3	-1.73	0.00
1507	NDUFA3	-1.73	0.00
1508	NDUFA3	-1.73	0.00
1509	NDUFA3	-1.73	0.00
1510	NDUFA3	-1.73	0.00
1511	NDUFA3	-1.73	0.00
1512	NDUFA7	-1.35	0.00
1513	NDUFAF3	-1.65	0.00
1514	NDUFAF8	-1.31	0.00
1515	NDUFB1	-0.71	0.01
1516	NDUFB10	-0.75	0.00
1517	NDUFB4	-0.66	0.01
1518	NDUFB8	-0.67	0.01
1519	NEAT1	1.13	0.00
1520	NECAP2	-0.49	0.00
1521	NECTIN2	-1.15	0.00
1522	NEDD8	-0.80	0.00
1523	NEK9	-0.49	0.00
1524	NELFE	-0.69	0.00
1525	NENF	-0.91	0.00
1526	NF1	0.69	0.00
1527	NFAT5	0.79	0.00
1528	NFKB2	-0.60	0.00
1529	NFXL1	0.71	0.00
1530	NINJ1	-0.89	0.00
1531	NINJ2	-0.94	0.00
1532	NMB	-1.87	0.00
1533	NME1	-0.99	0.01
1534	NME2	-0.92	0.00
1535	NOLC1	-0.68	0.00
1536	NOM1	0.57	0.00
1537	NOP16	-0.80	0.00
1538	NOP53	-0.85	0.00
1539	NPHP3	0.48	0.00
1540	NPIPB12	0.97	0.00
1541	NPIPB4	0.56	0.00
1542	NPIPB5	0.70	0.00
1543	NPRL2	-0.87	0.00
1544	NR1H2	-0.80	0.00
1545	NR2C2AP	-1.18	0.00
1546	NRGN	-1.14	0.00
1547	NRM	-1.22	0.00

1548	NRM	-1.21	0.00
1549	NRM	-1.21	0.00
1550	NRM	-1.21	0.00
1551	NRM	-1.20	0.00
1552	NRM	-1.22	0.00
1553	NRM	-1.22	0.00
1554	NRXN2	-1.18	0.00
1555	NSUN5	-0.80	0.00
1556	NT5C	-0.83	0.01
1557	NTMT1	-0.92	0.00
1558	NUBP1	-0.79	0.00
1559	NUDC	-0.66	0.00
1560	NUDT1	-0.70	0.00
1561	NUDT22	-0.97	0.00
1562	NUP160	0.45	0.01
1563	NUP62	-0.53	0.01
1564	NUPR1	-0.95	0.00
1565	NUTM2B-AS1	0.58	0.00
1566	NXPE4	1.11	0.01
1567	NXPH3	-1.36	0.00
1568	OAF	-0.91	0.00
1569	OBI1-AS1	1.15	0.00
1570	OCEL1	-0.98	0.00
1571	OCLM	0.74	0.00
1572	OGG1	-0.69	0.00
1573	OLFM1	-1.21	0.01
1574	OLFML3	-0.97	0.00
1575	OMG	0.73	0.00
1576	OR51B5	0.88	0.00
1577	ORAI3	-0.78	0.00
1578	ORC4	0.61	0.00
1579	OTOG	2.08	0.01
1580	OXA1L	-1.32	0.00
1581	PACSIN1	-0.98	0.01
1582	PACSIN3	-0.92	0.01
1583	PAF1	-0.82	0.00
1584	PAQR7	-0.92	0.00
1585	PARD6A	-1.16	0.00
1586	PARK7	-0.55	0.01
1587	PARP3	-0.94	0.00
1588	PCAT2	1.52	0.00
1589	PCBP1-AS1	0.79	0.00
1590	PCDHB14	0.98	0.01
1591	PCED1A	-0.72	0.00
1592	PCMTD1	0.71	0.01
1593	PCSK2	0.99	0.00
1594	PDAP1	-0.57	0.00
1595	PDE10A	0.86	0.00
1596	PDE6B	-1.17	0.00
1597	PDE6G	-2.29	0.00

1598	PDK2	-1.82	0.00
1599	PDRG1	-0.95	0.00
1600	PDS5A	0.41	0.01
1601	PDXDC2P-NPIPB14P	0.59	0.00
1602	PDZK1	-2.09	0.00
1603	PEBP1	-0.94	0.00
1604	PEF1	-0.65	0.00
1605	PEMT	-0.88	0.00
1606	PET100	-0.96	0.00
1607	PEX10	-0.76	0.01
1608	PEX11G	-1.16	0.00
1609	PEX12	-0.73	0.00
1610	PEX19	-0.43	0.01
1611	PFDN5	-0.72	0.00
1612	PFDN6	-1.12	0.00
1613	PFDN6	-1.11	0.00
1614	PGF	-1.35	0.00
1615	PGLS	-0.80	0.00
1616	PGLYRP2	-3.20	0.00
1617	PHF1	-0.75	0.01
1618	PHF14	0.69	0.00
1619	PHF23	-0.74	0.00
1620	PHLPP1	0.68	0.00
1621	PHPT1	-1.18	0.00
1622	PIBF1	0.66	0.01
1623	PICALM	0.44	0.00
1624	PIGP	-0.87	0.00
1625	PIGT	-0.92	0.00
1626	PIGV	-1.23	0.00
1627	PIH1D1	-0.69	0.00
1628	PIK3CB	0.63	0.00
1629	PILRA	-0.95	0.00
1630	PIN1	-0.91	0.00
1631	PINK1	-0.70	0.00
1632	PINK1-AS	-0.68	0.01
1633	PITHD1	-0.54	0.00
1634	PITPNA-AS1	-1.38	0.00
1635	PKIG	-0.96	0.00
1636	PLAAT4	-1.60	0.00
1637	PLCD1	-0.80	0.01
1638	PLEKHJ1	-0.74	0.00
1639	PLG	0.64	0.00
1640	PLP2	-1.84	0.00
1641	PLPPR5	1.88	0.00
1642	PLTP	-1.18	0.00
1643	PM20D1	-1.54	0.01
1644	PMEL	-0.94	0.00
1645	PMVK	-0.96	0.00
1646	PNMA8B	-1.50	0.00
1647	POLA1	0.54	0.00

1648	POLD4	-1.30	0.00
1649	POLR2E	-1.08	0.00
1650	POLR2G	-0.94	0.00
1651	POLR2I	-1.02	0.00
1652	POLR2J	-0.70	0.01
1653	POLR2L	-1.43	0.00
1654	POLR3GL	-0.77	0.00
1655	POP5	-0.65	0.00
1656	PORCN	-1.24	0.00
1657	POU3F1	-2.22	0.00
1658	POU5F2	0.59	0.01
1659	PPBP	-1.39	0.00
1660	PPIAP46	0.72	0.00
1661	PPM1M	-0.77	0.00
1662	PPP1CA	-0.69	0.01
1663	PPP1R10	-0.47	0.00
1664	PPP1R14B-AS1	-1.62	0.00
1665	PPP1R15A	-0.76	0.00
1666	PPP1R18	-0.90	0.00
1667	PPP1R18	-0.86	0.01
1668	PPP1R18	-0.85	0.01
1669	PPP1R9B	-1.18	0.00
1670	PPP2R5E	0.38	0.00
1671	PPP3R1	0.66	0.00
1672	PQBP1	-0.75	0.00
1673	PRAF2	-1.21	0.00
1674	PRDX5	-0.82	0.00
1675	PRDX6	-0.63	0.01
1676	PREB	-0.64	0.00
1677	PRG4	-1.03	0.01
1678	PRH1	0.85	0.01
1679	PRICKLE4	-1.56	0.00
1680	PRKAR2A-AS1	-0.54	0.00
1681	PRLHR	1.06	0.00
1682	PRNCR1	0.67	0.00
1683	PROSER1	0.82	0.00
1684	PRPF31	-0.83	0.00
1685	PRPF31	-0.81	0.00
1686	PRR13	-0.76	0.00
1687	PRR29	-1.34	0.01
1688	PRRT3	-1.35	0.00
1689	PRRX2	-1.41	0.00
1690	PRSS36	-1.02	0.00
1691	PRXL2B	-0.96	0.01
1692	PRXL2B	-0.96	0.01
1693	PSD	-1.04	0.00
1694	PSENEN	-2.42	0.00
1695	PSMB8-AS1	-1.20	0.00
1696	PSMB8-AS1	-1.20	0.00
1697	PSMB8-AS1	-1.44	0.00

1698	PSMB8-AS1	-1.20	0.00
1699	PSMB9	-3.03	0.00
1700	PSMB9	-3.30	0.00
1701	PSMB9	-3.03	0.00
1702	PSMC3IP	-0.87	0.00
1703	PSMC5	-0.88	0.00
1704	PSMD4	-0.60	0.01
1705	PSME4	0.88	0.00
1706	PSMG3	-1.15	0.00
1707	PSORS1C1	-1.81	0.01
1708	PSORS1C1	-1.77	0.00
1709	PTBP1	-0.60	0.00
1710	PTBP2	0.64	0.01
1711	PTMS	-1.32	0.00
1712	PTPMT1	-0.81	0.00
1713	PTPN1	-0.90	0.00
1714	PTPN12	0.75	0.00
1715	PTPN5	0.64	0.01
1716	PTRH1	-1.13	0.00
1717	PTX3	1.52	0.01
1718	PURPL	1.33	0.01
1719	PYCARD-AS1	-1.66	0.00
1720	PYDC1	-4.37	0.00
1721	PYGO2	-0.73	0.00
1722	QARS1	-0.47	0.01
1723	R3HDM4	-0.76	0.00
1724	RAB1B	-1.02	0.00
1725	RAB24	-0.84	0.00
1726	RAB25	-0.84	0.01
1727	RAB34	-0.91	0.01
1728	RAB35	-0.47	0.01
1729	RAB4A	-0.51	0.01
1730	RAB5C	-0.56	0.00
1731	RAB8A	-0.55	0.00
1732	RAC2	-1.25	0.00
1733	RACK1	-0.70	0.00
1734	RAD21-AS1	0.53	0.01
1735	RAD51C	-0.61	0.01
1736	RAI2	-1.73	0.00
1737	RAMP1	-1.22	0.01
1738	RAMP2	-2.83	0.00
1739	RANBP17	0.79	0.00
1740	RANGRF	-1.19	0.00
1741	RAPGEF3	-1.05	0.00
1742	RAPGEFL1	-1.40	0.01
1743	RASSF1	-0.61	0.01
1744	RASSF1-AS1	-1.41	0.00
1745	RBM10	-0.59	0.00
1746	RBM15B	-0.62	0.00
1747	RBM8A	-0.57	0.00

1748	RBMX2	-0.50	0.00
1749	RBP7	-1.27	0.01
1750	REPS1	0.63	0.00
1751	RETREG2	-0.78	0.00
1752	REV1	0.39	0.01
1753	REX1BD	-0.84	0.01
1754	RFX5	-0.59	0.00
1755	RFXANK	-0.94	0.00
1756	RGP1	-0.61	0.00
1757	RGS1	1.75	0.00
1758	RGSL1	1.08	0.00
1759	RHBDD2	-0.92	0.00
1760	RHOC	-0.91	0.00
1761	RHOD	-1.18	0.00
1762	RIC8A	-0.72	0.00
1763	RICTOR	0.46	0.01
1764	RIOX1	-0.53	0.01
1765	RIPK3	-0.91	0.00
1766	RIPOR3	-1.07	0.00
1767	RITA1	-0.77	0.00
1768	RNASE1	-1.44	0.00
1769	RNASEK	-1.30	0.00
1770	RNASEK-C17orf49	-1.48	0.01
1771	RNF10	0.40	0.00
1772	RNF113A	-0.79	0.00
1773	RNF149	0.39	0.00
1774	RNF167	-1.04	0.00
1775	RNF185	-0.61	0.01
1776	RNF220	-0.61	0.00
1777	RNF25	-0.61	0.00
1778	RNH1	-0.72	0.00
1779	RNH1	-0.69	0.00
1780	RNPC3	0.63	0.00
1781	RNU12	1.06	0.00
1782	RNU5A-1	1.32	0.01
1783	RNU5B-1	1.41	0.01
1784	ROBO3	-1.33	0.00
1785	ROCK1	0.57	0.00
1786	ROM1	-1.12	0.00
1787	RPA2	-0.46	0.00
1788	RPL11	-0.68	0.00
1789	RPL12	-0.83	0.00
1790	RPL13A	-0.96	0.00
1791	RPL14	-0.91	0.00
1792	RPL18	-0.82	0.00
1793	RPL22	-0.68	0.00
1794	RPL28	-0.93	0.00
1795	RPL29	-0.84	0.01
1796	RPL3	-0.74	0.00
1797	RPL37A	-0.75	0.01

1798	RPL41	-0.94	0.00
1799	RPL8	-1.02	0.00
1800	RPLP0	-0.89	0.00
1801	RPLP1	-0.94	0.00
1802	RPP25	-1.31	0.00
1803	RPS11	-0.63	0.01
1804	RPS15	-1.73	0.00
1805	RPS17	-0.91	0.00
1806	RPS17	-0.91	0.00
1807	RPS2	-0.82	0.00
1808	RPS26	-1.00	0.00
1809	RPSA	-1.02	0.00
1810	RPUSD3	-0.72	0.00
1811	RRAGC	0.37	0.00
1812	RRAS	-1.18	0.00
1813	RRP9	-1.06	0.00
1814	RSAD1	-0.71	0.00
1815	RSPO1	-2.64	0.00
1816	RTN2	-1.30	0.00
1817	RTN4	0.54	0.00
1818	RTP4	-1.03	0.00
1819	RTTN	0.52	0.01
1820	RUNDC3A	-1.29	0.00
1821	RUVBL2	-0.59	0.00
1822	RYBP	-1.00	0.00
1823	S100A11	-0.97	0.00
1824	S100A13	-1.64	0.00
1825	S100A2	-2.22	0.00
1826	S100A4	-1.18	0.01
1827	SAFB2	0.50	0.01
1828	SARS1	-0.62	0.00
1829	SART1	-0.51	0.01
1830	SASH3	-0.96	0.00
1831	SAT2	-0.91	0.00
1832	SCAMP1-AS1	-1.01	0.00
1833	SCARNA10	0.96	0.01
1834	SCFD1	0.66	0.00
1835	SCN1A	0.62	0.00
1836	SCN1B	-0.89	0.00
1837	SCN4B	-1.37	0.00
1838	SCNM1	-0.74	0.00
1839	SCRN2	-1.27	0.00
1840	SDCBP2-AS1	0.55	0.00
1841	SDR39U1	-1.03	0.00
1842	SDSL	-0.99	0.00
1843	SEC63	0.65	0.00
1844	SECISBP2L	0.48	0.01
1845	SELPLG	-1.16	0.00
1846	SENP3	-0.37	0.00
1847	SENP6	0.52	0.00

1848	SEPTIN4	-0.83	0.00
1849	SERPINA1	-2.13	0.00
1850	SERPINF1	-0.79	0.00
1851	SERPING1	-1.40	0.00
1852	SH2D3C	-0.98	0.00
1853	SH3BGRL3	-2.01	0.00
1854	SHARPIN	-0.94	0.00
1855	SHFL	-0.98	0.00
1856	SHISA5	-1.10	0.00
1857	SHPRH	0.54	0.01
1858	SIAE	-0.89	0.01
1859	SIRT2	-0.46	0.01
1860	SIRT2	-0.46	0.01
1861	SIX5	-0.98	0.00
1862	SLC10A1	-1.01	0.00
1863	SLC10A2	0.99	0.00
1864	SLC12A5	-1.69	0.00
1865	SLC16A13	-0.85	0.00
1866	SLC16A5	-1.38	0.01
1867	SLC1A2	-1.64	0.01
1868	SLC22A8	2.68	0.00
1869	SLC25A11	-0.92	0.00
1870	SLC25A21-AS1	-1.04	0.00
1871	SLC25A28	-0.77	0.00
1872	SLC25A36	0.52	0.00
1873	SLC25A38	-0.85	0.00
1874	SLC25A39	-1.09	0.00
1875	SLC25A6	-0.95	0.00
1876	SLC26A3	2.97	0.00
1877	SLC35B1	-0.97	0.00
1878	SLC38A6	0.38	0.01
1879	SLC39A3	-0.83	0.00
1880	SLC39A4	-1.19	0.00
1881	SLC46A1	-0.63	0.01
1882	SLC48A1	-0.90	0.00
1883	SLC4A11	1.77	0.00
1884	SLC6A9	-1.31	0.01
1885	SLC9A7P1	-0.85	0.01
1886	SMARCA5-AS1	-1.05	0.00
1887	SMARCB1	-0.72	0.00
1888	SMG1	0.60	0.00
1889	SMG1P1	0.60	0.00
1890	SMG1P7	0.49	0.01
1891	SMIM10	-0.89	0.00
1892	SMIM11A	-1.80	0.00
1893	SMIM17	1.18	0.00
1894	SMIM27	-0.70	0.00
1895	SMIM29	-0.93	0.00
1896	SMIM34A	-3.22	0.00
1897	SMIM4	-1.07	0.00

1898	SMPD2	-0.82	0.00
1899	SNAPC2	-0.97	0.00
1900	SNAPC5	-0.67	0.00
1901	SNF8	-0.81	0.00
1902	SNHG19	-1.09	0.01
1903	SNHG31	0.59	0.00
1904	SNHG32	-0.67	0.01
1905	SNHG32	-0.77	0.00
1906	SNHG32	-0.82	0.00
1907	SNHG32	-0.77	0.00
1908	SNHG9	-2.32	0.00
1909	SNORA11D	1.77	0.00
1910	SNORA11E	1.77	0.00
1911	SNORA18	1.89	0.00
1912	SNORA23	1.13	0.00
1913	SNORA2A	1.25	0.00
1914	SNORA33	1.02	0.00
1915	SNORA35B	1.72	0.00
1916	SNORA3A	1.43	0.01
1917	SNORA44	1.36	0.01
1918	SNORA53	1.16	0.00
1919	SNORA61	1.78	0.00
1920	SNORA74B	0.85	0.00
1921	SNORA79B	1.46	0.01
1922	SNORA9	1.88	0.01
1923	SNORD105B	1.80	0.00
1924	SNORD116-24	0.91	0.01
1925	SNORD17	0.86	0.00
1926	SNORD28	2.76	0.01
1927	SNORD3C	1.40	0.00
1928	SNORD46	1.91	0.00
1929	SNORD53	0.94	0.00
1930	SNORD71	0.89	0.00
1931	SNRNP25	-0.84	0.00
1932	SNRNP35	-0.48	0.01
1933	SNRPC	-0.86	0.00
1934	SNTG1	0.99	0.00
1935	SNUPN	-0.50	0.00
1936	SNX12	-0.42	0.00
1937	SNX14	0.47	0.00
1938	SNX15	-0.65	0.00
1939	SNX32	-0.79	0.01
1940	SNX33	-0.81	0.00
1941	SORCS1	-2.92	0.00
1942	SOX2-OT	0.50	0.01
1943	SOX30	-1.83	0.00
1944	SPACA9	-0.80	0.00
1945	SPAG7	-0.71	0.00
1946	SPATA20	-0.81	0.00
1947	SPDYE5	0.95	0.00

1948	SPI1	-0.88	0.00
1949	SPNS1	-0.62	0.01
1950	SRARP	-1.79	0.01
1951	SRP14	-0.64	0.01
1952	SSBP3-AS1	0.72	0.00
1953	SSC4D	-1.12	0.01
1954	ST8SIA5	1.78	0.00
1955	STAG1	0.43	0.01
1956	STAM-AS1	-0.78	0.00
1957	STARD10	-1.53	0.00
1958	STC1	-1.43	0.01
1959	STING1	-1.29	0.00
1960	STK16	-0.70	0.01
1961	STN1	-0.48	0.00
1962	STRN3	0.61	0.01
1963	STUB1	-0.61	0.01
1964	STX10	-0.83	0.00
1965	STX4	-0.54	0.01
1966	STXBP5L	1.74	0.00
1967	SUGP1	-0.56	0.00
1968	SULT1B1	0.67	0.01
1969	SUMO4	0.78	0.00
1970	SUPT7L	-0.46	0.00
1971	SURF1	-1.01	0.00
1972	SURF1	-1.01	0.00
1973	SURF2	-0.81	0.00
1974	SURF2	-0.81	0.00
1975	SURF6	-0.71	0.00
1976	SURF6	-0.71	0.00
1977	SUSD3	-2.55	0.00
1978	SUSD4	-1.57	0.00
1979	SUZ12	0.66	0.00
1980	SVBP	-0.62	0.01
1981	SVOP	1.38	0.00
1982	SWI5	-0.95	0.00
1983	SWSAP1	-0.69	0.00
1984	SYCE1L	-1.11	0.01
1985	SYF2	-0.49	0.01
1986	SYT13	-2.87	0.00
1987	SYT9	-1.56	0.00
1988	SZRD1	-0.51	0.01
1989	TADA2A	1.07	0.00
1990	TADA3	-0.60	0.01
1991	TAF10	-1.02	0.00
1992	TAF9	-0.56	0.01
1993	TAPBPL	-0.96	0.00
1994	TBC1D15	0.47	0.00
1995	TBC1D32	0.70	0.00
1996	TBC1D8B	0.41	0.01
1997	TBCB	-0.82	0.01

1998	TCEAL2	-1.38	0.00
1999	TCEAL3	-1.14	0.00
2000	TCERG1	0.54	0.00
2001	TCTA	-1.22	0.00
2002	TDRD12	1.83	0.00
2003	TECR	-1.02	0.00
2004	TEX101	-5.07	0.00
2005	TEX12	1.53	0.00
2006	TEX264	-0.80	0.01
2007	TFE3	-0.75	0.00
2008	TFIP11	-0.51	0.00
2009	TGFBR3	-1.94	0.00
2010	TH2LCRR	0.85	0.01
2011	THADA	0.49	0.00
2012	THAP3	-0.55	0.00
2013	THAP4	-0.66	0.01
2014	THAP7	-1.17	0.00
2015	THBS4	-1.14	0.00
2016	THY1	-1.46	0.00
2017	TIA1	0.53	0.01
2018	TIAL1	0.59	0.00
2019	TIE1	-0.88	0.01
2020	TIMM13	-1.59	0.00
2021	TIMM22	-0.69	0.00
2022	TIMM22	-0.69	0.00
2023	TIMM22	-0.69	0.00
2024	TIMP1	-1.48	0.00
2025	TINF2	-0.47	0.01
2026	TIRAP	-0.36	0.01
2027	TLCD2	-0.78	0.00
2028	TLCD2	-0.78	0.00
2029	TLE5	-1.08	0.00
2030	TLR5	-0.76	0.00
2031	TMA7	-0.89	0.00
2032	TMED1	-0.72	0.01
2033	TMED3	-0.87	0.01
2034	TMEM102	-0.76	0.00
2035	TMEM102	-0.76	0.00
2036	TMEM11	-0.65	0.00
2037	TMEM115	-1.01	0.00
2038	TMEM125	-1.29	0.00
2039	TMEM129	-0.70	0.00
2040	TMEM131	0.54	0.00
2041	TMEM131L	0.81	0.01
2042	TMEM150A	-0.75	0.00
2043	TMEM160	-1.15	0.00
2044	TMEM175	-0.75	0.00
2045	TMEM179B	-0.84	0.00
2046	TMEM189	-0.90	0.01
2047	TMEM204	-0.72	0.00

2048	TMEM205	-1.38	0.00
2049	TMEM214	-0.65	0.00
2050	TMEM216	-0.71	0.00
2051	TMEM219	-0.94	0.00
2052	TMEM223	-0.69	0.00
2053	TMEM229B	-0.76	0.00
2054	TMEM259	-0.74	0.01
2055	TMEM30B	-0.93	0.00
2056	TMEM42	-0.92	0.00
2057	TMEM42	-0.92	0.00
2058	TMEM69	-1.02	0.00
2059	TMPRSS11A	1.25	0.01
2060	TMPRSS11BNL	0.95	0.01
2061	TMPRSS12	0.62	0.01
2062	TMPRSS3	-2.43	0.00
2063	TMUB1	-0.64	0.01
2064	TMUB2	-0.81	0.00
2065	TNFAIP8L2	-0.83	0.00
2066	TNFRSF14	-0.89	0.00
2067	TNFRSF14	-0.91	0.00
2068	TNFRSF4	-1.29	0.01
2069	TNFSF12	-0.75	0.01
2070	TNIP2	-0.59	0.00
2071	TOE1	-0.88	0.00
2072	TOLLIP-AS1	-1.54	0.00
2073	TRAPPC2L	-0.83	0.00
2074	TRAPPC3L	0.56	0.01
2075	TREX1	-1.29	0.00
2076	TRIM52-AS1	-1.13	0.00
2077	TRIM55	-2.51	0.00
2078	TRIM68	-0.71	0.01
2079	TRIO	0.58	0.00
2080	TRIR	-1.17	0.00
2081	TRMT11	0.77	0.00
2082	TRPT1	-1.09	0.00
2083	TSC22D2	0.70	0.00
2084	TSR2	-0.96	0.00
2085	TSR3	-0.77	0.00
2086	TSSK6	-0.87	0.01
2087	TTC31	-0.43	0.00
2088	TTC33	0.48	0.00
2089	TTC36	-1.43	0.01
2090	TTC3-AS1	0.73	0.00
2091	TUBB1	-0.88	0.00
2092	TUBG1	-0.72	0.00
2093	TUBG2	-0.82	0.01
2094	TUT1	-0.88	0.00
2095	TWIST2	-1.29	0.00
2096	TWNK	-1.06	0.00
2097	TXLNA	-0.51	0.00

2098	TYROBP	-1.16	0.00
2099	U2AF1L4	-0.72	0.00
2100	U2AF2	-0.64	0.00
2101	UBA52	-1.05	0.00
2102	UBA7	-0.80	0.00
2103	UBALD2	-1.54	0.00
2104	UBE2L3	-0.74	0.00
2105	UBE2M	-0.70	0.00
2106	UBE2Q1	-0.63	0.00
2107	UBE3C	0.58	0.00
2108	UBE4B	0.32	0.01
2109	UBL5	-0.98	0.01
2110	UBL7	-0.89	0.00
2111	UBQLN3	1.40	0.00
2112	UBR3	0.58	0.00
2113	UBTF	-0.38	0.00
2114	UBXN1	-1.09	0.00
2115	UGGT2	0.74	0.00
2116	UNC119	-0.82	0.01
2117	UQCR10	-0.88	0.01
2118	UQCR11	-1.64	0.00
2119	URB1-AS1	-1.11	0.00
2120	UROD	-0.55	0.01
2121	USE1	-0.67	0.00
2122	USF2	-0.73	0.00
2123	USP27X	-0.49	0.00
2124	USP27X-AS1	-1.17	0.00
2125	USP34	0.57	0.00
2126	USP50	0.92	0.00
2127	USP7	0.38	0.00
2128	USP9X	0.53	0.00
2129	UTP3	-0.49	0.00
2130	UXT	-0.75	0.00
2131	VAMP5	-1.35	0.00
2132	VILL	-0.83	0.01
2133	VMO1	-1.99	0.00
2134	VMP1	0.64	0.01
2135	VPS11	-0.65	0.00
2136	VPS11	-0.65	0.00
2137	VPS28	-1.01	0.00
2138	VPS36	0.59	0.00
2139	VPS8	0.65	0.00
2140	VSIG2	-2.12	0.00
2141	VTI1B	-0.59	0.00
2142	WDFY2	0.71	0.00
2143	WDR34	-0.95	0.01
2144	WDR45	-0.79	0.00
2145	WDR54	-0.68	0.01
2146	WDR59	0.52	0.00
2147	WDR6	-0.62	0.01

2148	WDR72	0.89	0.00
2149	WDR77	-0.75	0.00
2150	WDR83	-0.61	0.00
2151	WDR83OS	-0.86	0.01
2152	WDTC1	-0.45	0.00
2153	WEE1	0.57	0.00
2154	WFDC2	-2.06	0.00
2155	WRAP53	-1.04	0.00
2156	WT1	-2.32	0.00
2157	WTIP	-0.99	0.00
2158	XCL1	-1.57	0.00
2159	XYLT2	-0.91	0.00
2160	YAF2	0.60	0.00
2161	YDJC	-1.16	0.00
2162	YIF1B	-0.94	0.00
2163	YIPF3	-0.88	0.00
2164	YLPM1	0.57	0.00
2165	YPEL4	-1.25	0.01
2166	YTHDF3-AS1	-1.45	0.00
2167	ZBED5-AS1	-0.76	0.01
2168	ZBTB2	-1.00	0.00
2169	ZBTB4	-0.64	0.00
2170	ZBTB4	-0.64	0.00
2171	ZBTB47	-1.13	0.00
2172	ZC3H10	-0.82	0.00
2173	ZC3H4	-0.37	0.00
2174	ZC3H7A	0.52	0.00
2175	ZCCHC7	0.55	0.00
2176	ZCCHC8	0.31	0.01
2177	ZDHHC15	0.49	0.01
2178	ZDHHC21	0.93	0.00
2179	ZFAND2A	-0.62	0.01
2180	ZFAND2B	-0.77	0.00
2181	ZFC3H1	0.60	0.00
2182	ZFP3	-0.44	0.00
2183	ZFPL1	-0.77	0.00
2184	ZFYVE1	-0.37	0.00
2185	ZIM2	-2.21	0.00
2186	ZMAT5	-0.81	0.01
2187	ZMYM2	0.50	0.01
2188	ZNF142	-0.37	0.00
2189	ZNF18	-0.36	0.00
2190	ZNF292	0.60	0.00
2191	ZNF324	-0.64	0.00
2192	ZNF324B	-0.59	0.00
2193	ZNF335	-0.49	0.00
2194	ZNF384	-0.62	0.00
2195	ZNF385B	-2.05	0.00
2196	ZNF394	-0.37	0.00
2197	ZNF404	0.63	0.00

2198	ZNF430	0.62	0.00
2199	ZNF436-AS1	-0.81	0.00
2200	ZNF513	-0.67	0.00
2201	ZNF524	-1.21	0.00
2202	ZNF526	-0.44	0.01
2203	ZNF574	-0.66	0.00
2204	ZNF576	-0.76	0.00
2205	ZNF580	-0.91	0.00
2206	ZNF619	-0.47	0.00
2207	ZNF629	-0.68	0.00
2208	ZNF638	0.53	0.00
2209	ZNF646	-0.69	0.00
2210	ZNF672	-0.60	0.00
2211	ZNF687	-0.65	0.00
2212	ZNF688	-0.56	0.00
2213	ZNF705E	0.85	0.00
2214	ZNF747	-0.72	0.00
2215	ZNF768	-0.82	0.00
2216	ZNF830	-0.60	0.00
2217	ZNF853	-1.34	0.00
2218	ZNRD2	-0.95	0.00
2219	ZPBP2	0.72	0.00
2220	ZPLD1	3.35	0.00
2221	ZPR1	-0.61	0.00
2222	ZSCAN22	-0.53	0.00

ıors

Table S3: Differentially expressed genes upon progesterone treatment to T47D (PR+/ER+/Her2-) cell line

S.No.	Gene	Log2FoldChange	P-value
1	*AC012358.8*	1.34	0.02
2	*AC093323.3*	1.23	0.00
3	*ACACB*	-1.43	0.04
4	*ACER2*	1.55	0.00
5	*ACHE*	-1.64	0.04
6	*ACSL1*	0.94	0.00
7	*ACSM2B*	-2.09	0.04
8	*ACSS1*	0.81	0.01
9	*ADD3*	0.99	0.03
10	*ADGB*	-3.80	0.05
11	*ADRA1A*	-2.81	0.00
12	*AGR3*	0.84	0.03
13	*AMIGO2*	-1.68	0.00
14	*ANKRD22*	1.15	0.00
15	*ANKRD35*	3.31	0.00
16	*ANKS6*	-2.57	0.03
17	*ANO6*	0.79	0.02
18	*ANPEP*	-2.29	0.03
19	*APOL3*	-2.79	0.00
20	*APOL4*	-2.62	0.03
21	*AQP3*	0.94	0.03
22	*ARHGEF26*	1.62	0.00
23	*ARL4C*	1.59	0.01
24	*ARL4D*	2.10	0.00
25	*ARRB1*	0.97	0.00
26	*ATAD2*	1.68	0.00
27	*ATP10A*	1.41	0.00
28	*ATP1A1*	1.22	0.00
29	*ATP1B1*	1.46	0.00
30	*AZGP1*	1.16	0.00
31	*B4GALT1*	0.73	0.02
32	*BAMBI*	-0.91	0.01
33	*BCL11A*	-2.74	0.01
34	*BCL6*	1.22	0.00
35	*BNIP3*	0.83	0.03
36	*C10orf12*	0.85	0.01
37	*C6orf141*	-0.95	0.01
38	*C9orf152*	1.58	0.00
39	*CADPS*	-3.15	0.01
40	*CAMKK2*	0.69	0.03
41	*CASC10*	1.34	0.04
42	*CASD1*	0.93	0.03
43	*CASP7*	1.13	0.00
44	*CBX4*	0.66	0.04
45	*CD163*	-2.41	0.03
46	*CD209*	-3.02	0.01
47	*CDC42EP3*	1.36	0.00
48	*CDH13*	-1.45	0.04
49	*CDHR1*	-2.72	0.04

50	*CEBPD*	2.07	0.00
51	*CHPT1*	0.99	0.00
52	*CHRNA4*	-4.67	0.01
53	*CLDN8*	1.96	0.02
54	*CLEC4M*	-4.07	0.00
55	*CLIC6*	0.98	0.00
56	*CLMN*	1.01	0.01
57	*CNN1*	-2.53	0.05
58	*CNTNAP3*	-3.03	0.03
59	*COPZ2*	1.32	0.04
60	*CORO2A*	1.15	0.00
61	*CRB1*	-3.36	0.04
62	*CREB3L2*	0.96	0.01
63	*CSH1*	-3.76	0.04
64	*CSH2*	-3.88	0.00
65	*CSRNP1*	1.24	0.00
66	*CTB-92J24.3*	1.96	0.03
67	*CTNNA2*	-2.34	0.03
68	*CTTNBP2NL*	1.03	0.02
69	*CXCL12*	-0.81	0.02
70	*CXCR4*	-1.68	0.00
71	*CYP4A11*	-3.95	0.02
72	*CYP4F3*	-2.32	0.01
73	*DBI*	0.76	0.01
74	*DCC*	-3.62	0.01
75	*DCLK2*	-1.60	0.01
76	*DENND1B*	0.81	0.04
77	*DESI2*	0.99	0.02
78	*DFNB31*	1.19	0.00
79	*DGKH*	1.12	0.03
80	*DIXDC1*	1.05	0.00
81	*DLGAP1*	-3.02	0.01
82	*DLGAP2*	-3.30	0.05
83	*DLX3*	0.83	0.03
84	*DMBT1*	-2.61	0.01
85	*DNAJB4*	1.04	0.02
86	*DPCR1*	-1.90	0.04
87	*DPF3*	-2.14	0.05
88	*DPP10*	-4.62	0.00
89	*DSCAM-AS1*	-0.85	0.02
90	*DSPP*	-2.64	0.01
91	*DUSP1*	1.17	0.00
92	*DUSP19*	-1.47	0.04
93	*EAF2*	2.66	0.01
94	*EFHD1*	0.81	0.04
95	*EGF*	1.21	0.01
96	*ELMO1*	-2.91	0.01
97	*EMP2*	0.87	0.01
98	*ENPP1*	1.32	0.00
99	*EPGN*	2.36	0.02
100	*EPHB1*	-4.13	0.02
101	*ERC2*	-4.56	0.00

102	*ERRFI1*	1.68	0.00
103	*EXOC3L4*	-2.96	0.04
104	*EZR*	0.64	0.05
105	*F3*	2.76	0.00
106	*FADS6*	-2.93	0.05
107	*FAM102B*	0.93	0.01
108	*FAM107B*	1.80	0.00
109	*FAM46C*	0.97	0.01
110	*FAM81A*	-2.58	0.02
111	*FAM86B1*	-2.07	0.02
112	*FEM1C*	0.76	0.02
113	*FGD4*	1.21	0.00
114	*FGFBP2*	-1.82	0.05
115	*FKBP5*	2.24	0.00
116	*FLG*	-3.24	0.00
117	*FLRT3*	1.45	0.02
118	*FRK*	1.55	0.00
119	*FZD5*	0.98	0.01
120	*GADD45B*	1.21	0.00
121	*GCH1*	0.75	0.03
122	*GFAP*	-2.12	0.03
123	*GJA1*	2.52	0.00
124	*GJB2*	1.73	0.04
125	*GLUL*	1.29	0.01
126	*GNA12*	0.99	0.03
127	*GNAO1*	-2.76	0.03
128	*GNPTAB*	1.10	0.00
129	*GOLGA6L4*	-1.99	0.01
130	*GOLGA8B*	2.20	0.01
131	*GPR153*	0.92	0.05
132	*GPSM2*	1.19	0.03
133	*GRASP*	-3.28	0.04
134	*GRHL2*	1.02	0.00
135	*GRIA3*	-5.04	0.02
136	*GRIK4*	2.48	0.03
137	*GRPR*	-1.09	0.00
138	*GTF2A1L*	-1.00	0.04
139	*HAL*	-4.80	0.02
140	*HDAC9*	-1.85	0.02
141	*HERC5*	0.91	0.01
142	*HIF3A*	-2.66	0.01
143	*HILPDA*	1.49	0.00
144	*HK2*	0.80	0.02
145	*HMGCS2*	0.94	0.02
146	*HNRNPCL1*	-3.85	0.03
147	*HP*	-3.09	0.01
148	*HPGD*	1.98	0.02
149	*HR*	-0.95	0.01
150	*HRNR*	-2.87	0.00
151	*IER5L*	0.82	0.04
152	*IFI44L*	-2.66	0.03
153	*IFRD1*	1.11	0.00

154	*IGFBP5*	-0.82	0.02
155	*IL20RA*	1.52	0.00
156	*IL6R*	0.89	0.03
157	*IL6ST*	1.49	0.02
158	*IQCA1*	-3.09	0.04
159	*IQCF3*	-2.69	0.03
160	*IRF2BPL*	-0.69	0.04
161	*ITGA10*	0.93	0.04
162	*ITPKB*	1.24	0.00
163	*KANK1*	1.41	0.00
164	*KCNC4*	2.16	0.01
165	*KCNH1*	1.60	0.02
166	*KCNJ11*	1.26	0.00
167	*KCNK5*	-1.81	0.01
168	*KCNMA1*	1.14	0.04
169	*KDM4B*	0.99	0.00
170	*KIAA0040*	0.83	0.02
171	*KIAA0232*	0.94	0.01
172	*KLF15*	1.18	0.02
173	*KLF4*	1.40	0.00
174	*KLF9*	1.52	0.00
175	*KLK2*	-2.73	0.00
176	*LAIR1*	-2.72	0.00
177	*LAMA3*	1.27	0.02
178	*LAMB3*	-0.81	0.04
179	*LCN8*	-3.39	0.05
180	*LEF1*	1.72	0.01
181	*LGALS9C*	-5.27	0.00
182	*LGI4*	-1.86	0.04
183	*LILRA1*	-4.05	0.00
184	*LILRA4*	-5.78	0.02
185	*LILRB1*	-3.29	0.01
186	*LILRB4*	-3.01	0.03
187	*LIMA1*	0.66	0.04
188	*LINC01137*	0.95	0.05
189	*LRIG3*	1.28	0.00
190	*LRRC31*	2.51	0.00
191	*LRRTM4*	-4.96	0.04
192	*LSP1*	-3.73	0.00
193	*MAFB*	1.58	0.00
194	*MAN1A1*	1.82	0.00
195	*MAN2A1*	0.70	0.03
196	*MAT2A*	0.82	0.03
197	*MBNL1*	0.83	0.05
198	*MCL1*	0.91	0.01
199	*MEFV*	-2.45	0.01
200	*MKX*	-1.12	0.04
201	*MMP15*	0.81	0.04
202	*MPHOSPH10*	1.14	0.00
203	*MRFAP1*	0.79	0.01
204	*MTCL1*	1.34	0.01
205	*MTMR9*	1.40	0.00

206	*MUC12*	-2.40	0.03
207	*MUC17*	-3.37	0.05
208	*MUC2*	-2.69	0.02
209	*MUC22*	-2.22	0.04
210	*MYB*	-0.97	0.02
211	*MYBL1*	1.10	0.00
212	*MYBPC1*	2.49	0.00
213	*MYO1A*	-2.87	0.00
214	*MYOM1*	-4.83	0.04
215	*NADK2*	0.83	0.03
216	*NAMPT*	1.10	0.01
217	*NAP1L4*	0.83	0.03
218	*NBEA*	1.29	0.01
219	*NBPF14*	1.20	0.02
220	*NBPF19*	1.63	0.00
221	*NBPF9*	1.16	0.00
222	*NEDD9*	1.52	0.00
223	*NET1*	1.54	0.00
224	*NETO2*	2.05	0.01
225	*NFIL3*	0.95	0.03
226	*NFKBIA*	1.08	0.00
227	*NKX2-1*	-3.44	0.00
228	*NLRP3*	-3.46	0.01
229	*NOL4*	-2.56	0.02
230	*NOTCH2*	1.05	0.01
231	*NPC1*	0.77	0.05
232	*NPIPB11*	-1.89	0.02
233	*NRG1*	-2.33	0.01
234	*NRXN3*	-3.06	0.01
235	*NTM*	-2.83	0.01
236	*NUDT18*	-0.91	0.02
237	*OPRD1*	-1.39	0.05
238	*PACSIN1*	1.00	0.01
239	*PAG1*	1.35	0.05
240	*PARP14*	1.02	0.00
241	*PARP9*	1.01	0.02
242	*PCDH18*	-2.11	0.01
243	*PCED1B-AS1*	-2.75	0.03
244	*PDK4*	1.79	0.01
245	*PEG3*	-3.26	0.00
246	*PFKFB3*	0.82	0.02
247	*PGA3*	-3.60	0.02
248	*PHF8*	0.70	0.05
249	*PHYHIP*	-3.91	0.03
250	*PIAS3*	0.79	0.03
251	*PIK3R1*	0.78	0.03
252	*PISD*	1.35	0.01
253	*PKDCC*	-2.21	0.04
254	*PLA2G3*	1.24	0.01
255	*PLA2G4C*	1.60	0.04
256	*PLEKHD1*	1.60	0.00
257	*PLEKHF1*	1.04	0.01

258	*PLEKHF2*	0.83	0.02
259	*PLEKHG2*	1.27	0.04
260	*PNN*	0.69	0.04
261	*POU2AF1*	-3.61	0.01
262	*PPL*	1.24	0.02
263	*PPP1R14C*	2.91	0.04
264	*PPP1R36*	-2.34	0.04
265	*PRDM1*	1.91	0.03
266	*PRR15*	0.76	0.02
267	*PRR15L*	1.25	0.02
268	*PSG1*	-3.96	0.01
269	*PSG11*	-2.92	0.02
270	*PSG2*	-3.23	0.00
271	*PSG4*	-3.68	0.00
272	*PSG5*	-5.10	0.01
273	*PSG8*	-2.26	0.04
274	*PTHLH*	-1.68	0.04
275	*PTPRJ*	0.79	0.01
276	*RAB3B*	2.79	0.01
277	*RANBP3L*	3.89	0.00
278	*RASA2*	0.99	0.04
279	*RBBP7*	0.74	0.04
280	*RBBP8NL*	-0.94	0.02
281	*RBFOX1*	-2.96	0.01
282	*RBM24*	0.96	0.01
283	*RCAN1*	1.48	0.00
284	*RGSL1*	-3.21	0.00
285	*RIMS1*	-1.38	0.02
286	*RP11-175O19.4*	1.03	0.01
287	*RP11-21L23.2*	0.98	0.02
288	*RP11-34P13.8*	1.83	0.01
289	*RP11-363E7.4*	1.18	0.01
290	*RP11-367J11.2*	1.05	0.04
291	*RP11-73M7.6*	-2.88	0.00
292	*RPF2*	0.77	0.04
293	*RPH3AL*	1.57	0.03
294	*RPS6KA1*	0.73	0.02
295	*RPS6KA5*	1.53	0.02
296	*RRS1-AS1*	1.16	0.03
297	*RUNX1*	1.25	0.00
298	*S100P*	1.42	0.01
299	*SAMD3*	1.93	0.03
300	*SASH1*	1.29	0.00
301	*SAT1*	1.24	0.00
302	*SCNN1G*	2.25	0.01
303	*SCUBE2*	1.57	0.00
304	*SEC62*	0.92	0.01
305	*SEMA6A*	-1.09	0.01
306	*SERINC5*	1.09	0.01
307	*SETD7*	0.77	0.02
308	*SGK1*	3.02	0.00
309	*SHISA2*	-0.95	0.01

310	*SHROOM2*	-0.85	0.03
311	*SIM2*	1.17	0.01
312	*SLAIN1*	-2.58	0.03
313	*SLC22A15*	1.71	0.02
314	*SLC22A23*	1.59	0.00
315	*SLC25A17*	0.85	0.02
316	*SLC25A18*	2.01	0.00
317	*SLC34A1*	-3.54	0.05
318	*SLC6A13*	-2.73	0.02
319	*SLC7A7*	-2.02	0.02
320	*SLX1A*	-1.89	0.01
321	*SOCS3*	-1.45	0.05
322	*SOWAHA*	0.74	0.04
323	*SOX4*	0.88	0.01
324	*SPATA31A6*	-3.55	0.02
325	*SPATA31C1*	-3.86	0.01
326	*SPRED2*	1.43	0.00
327	*SRGAP2*	1.00	0.00
328	*SRM*	0.79	0.04
329	*ST3GAL4*	1.38	0.00
330	*ST8SIA5*	-7.24	0.01
331	*STEAP2*	0.93	0.03
332	*STOM*	1.13	0.00
333	*STRA6*	-3.10	0.00
334	*SYBU*	3.02	0.00
335	*TBC1D4*	1.08	0.00
336	*TCAF2*	1.12	0.01
337	*TCEB3*	0.81	0.02
338	*TCF7L2*	-1.66	0.00
339	*TCHH*	-1.52	0.03
340	*TECTA*	-3.29	0.01
341	*TES*	-2.62	0.02
342	*TEX35*	-1.51	0.05
343	*TGFA*	0.76	0.04
344	*TGFB1I1*	-2.07	0.04
345	*THBS1*	1.27	0.00
346	*TIPARP*	0.96	0.01
347	*TMEM191C*	-1.51	0.04
348	*TMTC2*	0.87	0.02
349	*TNFRSF10A*	0.99	0.04
350	*TNFSF15*	-1.19	0.05
351	*TNS3*	1.23	0.00
352	*TPPP2*	-1.90	0.04
353	*TRIML2*	-3.84	0.02
354	*TSC22D1*	0.87	0.02
355	*TSC22D3*	1.54	0.00
356	*TSKU*	0.68	0.03
357	*TTC39B*	1.05	0.01
358	*UGT1A1*	-2.90	0.00
359	*ULK2*	0.74	0.05
360	*VCX*	-3.42	0.03
361	*VDR*	0.99	0.00

362	*WWC1*	0.96	0.01
363	*XYLT1*	-2.31	0.03
364	*ZBTB10*	0.93	0.01
365	*ZBTB7C*	2.06	0.00
366	*ZC3H12A*	0.98	0.03
367	*ZCCHC6*	0.82	0.02
368	*ZEB2*	-1.48	0.03
369	*ZIC4*	-3.42	0.02
370	*ZNF423*	-3.28	0.02
371	*ZNF474*	-3.39	0.02
372	*ZNF521*	-3.68	0.01
373	*ZNF652*	1.30	0.00
374	*ZNF812*	1.90	0.00

Table S4: Differentially expressed genes upon progesterone treatment to MDA-MB-231 (PR-/ER-/Her2-) cell line

S.No.	Gene	Log2FoldChange	P-value
1	*ABCB1*	-3.97	0.02
2	*ABCC8*	-5.47	0.00
3	*ACSL6*	-1.77	0.03
4	*ADAMTS2*	-4.49	0.03
5	*ADORA3*	-3.84	0.01
6	*ADRA1A*	-2.10	0.02
7	*ALDH3A1*	-3.17	0.00
8	*ANKRD20A2*	-2.36	0.01
9	*ARHGAP15*	-2.76	0.05
10	*ARHGAP8*	-1.53	0.03
11	*ASB2*	0.60	0.02
12	*B3GNT3*	-1.48	0.03
13	*BCAS3*	0.71	0.03
14	*BORCS5*	-1.11	0.04
15	*C10orf11*	-3.54	0.01
16	*C15orf57*	-0.99	0.01
17	*C1QTNF5*	-3.60	0.02
18	*CACNA1A*	-3.76	0.00
19	*CADPS*	-2.36	0.05
20	*CAPN8*	-2.48	0.04
21	*CCNB3*	-2.05	0.03
22	*CD4*	-3.72	0.00
23	*CD6*	-3.11	0.03
24	*CDH11*	-2.01	0.03
25	*CDKN2A*	-1.77	0.05
26	*CELF4*	-1.85	0.02
27	*CERKL*	-1.63	0.02
28	*CFAP61*	-2.36	0.01
29	*CHIT1*	-5.93	0.02
30	*CHRD*	-4.18	0.04
31	*CHRNA2*	-3.50	0.01
32	*CHRNA3*	-2.62	0.04
33	*CHRNA4*	-7.57	0.01
34	*CKMT1B*	-3.39	0.00
35	*COL1A2*	-2.23	0.00
36	*CPAMD8*	-3.38	0.00
37	*CPZ*	-3.35	0.00
38	*CSHL1,GH1*	-3.42	0.01
39	*CTNND2*	-4.12	0.00
40	*CYP21A2*	-2.83	0.02
41	*CYP4A11*	-3.99	0.02
42	*CYP4F12*	-4.83	0.00
43	*DAPK1*	-3.01	0.02
44	*DCC*	-4.72	0.00
45	*DCHS1*	-1.13	0.02
46	*DCHS2*	-2.75	0.03
47	*DCN*	-2.61	0.01
48	*DMD*	2.70	0.00
49	*DNAH9*	-5.22	0.00
50	*DOCK10*	1.17	0.04
51	*DOK7*	-2.27	0.03
52	*DPCR1*	-2.38	0.05
53	*DPEP2*	-3.54	0.02
54	*DPP6*	-3.87	0.01

55	*DRC7*	-5.38	0.01
56	*DSPP*	-2.78	0.02
57	*DUSP15*	-3.48	0.03
58	*EHHADH*	-1.08	0.01
59	*ELN*	-2.28	0.00
60	*EMCN*	6.05	0.01
61	*ENKD1*	-2.95	0.03
62	*EPB41L3*	-2.02	0.01
63	*ESPNL*	-4.23	0.05
64	*FAM53A*	-1.14	0.03
65	*FAM86B1*	-1.00	0.03
66	*FBN3*	-5.34	0.04
67	*FGD2*	-3.12	0.02
68	*FILIP1*	-1.39	0.02
69	*FLI1*	-2.16	0.03
70	*GAS7*	-5.22	0.00
71	*GCA*	-0.93	0.01
72	*GOLGA6L10*	-1.27	0.05
73	*GRM2*	-2.60	0.05
74	*GRM4*	-3.58	0.00
75	*GTF2A1L*	-1.44	0.04
76	*HOXA10*	-3.41	0.02
77	*HOXA3*	-3.73	0.00
78	*HP*	-5.44	0.00
79	*IRX4*	-2.75	0.05
80	*ITPR1*	-0.85	0.04
81	*KCNG1*	-3.20	0.03
82	*KCNMA1*	-3.85	0.00
83	*KIF1A*	-5.49	0.01
84	*KIF26B*	-1.36	0.03
85	*KLK2*	-2.17	0.01
86	*KLK8*	-2.60	0.02
87	*KRTAP10-1*	-2.57	0.03
88	*LCN10*	-3.12	0.01
89	*LGALS9C*	-5.01	0.00
90	*LGI1*	-3.49	0.01
91	*LILRA1*	-2.44	0.01
92	*LILRB1*	-2.49	0.04
93	*LRRC36*	-3.66	0.00
94	*LY9*	-3.25	0.01
95	*MARK1*	-2.12	0.04
96	*MASP1*	-5.31	0.00
97	*MCF2L*	-2.05	0.01
98	*MEFV*	-2.20	0.02
99	*MEI1*	-1.88	0.05
100	*Metazoa_SRP*	-3.57	0.00
101	*MOG*	-5.10	0.00
102	*MPP2*	-1.24	0.02
103	*MT-ND6*	-0.89	0.00
104	*MUC17*	-3.20	0.02
105	*NAALADL1*	-3.07	0.00
106	*NCALD*	-3.68	0.00
107	*NEU4*	-2.52	0.02
108	*NFATC4*	-2.58	0.00
109	*NKX2-1*	-2.26	0.01
110	*NOS3*	-2.33	0.05
111	*NPNT*	-2.50	0.04

112	*NRCAM*	-1.19	0.04
113	*NRXN2*	-2.21	0.05
114	*OBSCN*	-1.25	0.03
115	*OLFM1*	-3.36	0.01
116	*OPRM1*	-4.88	0.00
117	*PARVB*	-2.98	0.00
118	*PBX1*	-1.32	0.04
119	*PCDHA1*	-1.42	0.00
120	*PDE9A*	-2.24	0.02
121	*PDZRN3*	-5.13	0.00
122	*PGA3*	-3.36	0.04
123	*PHOSPHO1*	-4.22	0.02
124	*PIK3R5*	-5.34	0.00
125	*PLA1A*	-4.59	0.03
126	*PNCK*	-2.24	0.00
127	*POTEH*	-2.76	0.03
128	*POU2AF1*	-2.79	0.04
129	*PPIL3*	0.67	0.02
130	*PRDM16*	-5.46	0.00
131	*PRKCI*	0.70	0.04
132	*PTGIR*	-3.81	0.05
133	*PTK7*	-1.48	0.04
134	*PTPN7*	-1.97	0.03
135	*RBFOX3*	-3.09	0.01
136	*RHOJ*	-1.68	0.04
137	*RIMBP3*	-1.51	0.01
138	*RIMS2*	-1.25	0.05
139	*RNF212*	-3.73	0.00
140	*RP11-92C4.3*	-3.34	0.00
141	*RP3-395M20.8*	2.20	0.04
142	*RP5-1101C3.1*	-2.18	0.01
143	*SAG*	-4.62	0.01
144	*SALL1*	-3.44	0.05
145	*SAMD3*	-1.77	0.01
146	*SELPLG*	-1.30	0.04
147	*SEMA3A*	-3.86	0.01
148	*SERPINA3*	-1.82	0.01
149	*SH2D3C*	-2.02	0.03
150	*SHANK1*	-2.59	0.05
151	*SIGLEC14*	-4.60	0.04
152	*SLC12A5*	-2.59	0.01
153	*SLC13A5*	-4.45	0.01
154	*SLC2A14*	-1.48	0.03
155	*SLC38A5*	-2.92	0.00
156	*SLC4A10*	-2.93	0.00
157	*SOX9-AS1*	1.95	0.01
158	*SPATA22*	-3.37	0.00
159	*SPATA31C1*	-3.13	0.05
160	*ST8SIA4*	-1.19	0.02
161	*STAB1*	-7.26	0.01
162	*STAT4*	-2.85	0.03
163	*STAT5A*	-1.16	0.01
164	*STRC*	-3.73	0.02
165	*STYK1*	1.10	0.03
166	*SYNPO2*	-1.22	0.04
167	*SYT1*	-1.67	0.05
168	*TARSL2*	-1.46	0.01

169	*TASP1*	0.83	0.02
170	*TBC1D3B*	-1.06	0.02
171	*TCEB3C*	-2.82	0.00
172	*TCF4*	-1.24	0.01
173	*TCHH*	-1.83	0.01
174	*TCP10*	-4.07	0.03
175	*TCP11*	-2.35	0.00
176	*TEX41*	1.89	0.00
177	*TG*	-3.85	0.00
178	*THBS4*	-2.90	0.04
179	*THNSL2*	-3.23	0.03
180	*THSD7B*	-2.26	0.02
181	*TMEM108*	-4.36	0.00
182	*TMEM136*	0.66	0.04
183	*TMEM221*	-2.23	0.04
184	*TMPRSS13*	-3.09	0.04
185	*TNIP3*	0.99	0.04
186	*TP63*	-2.17	0.02
187	*TPPP2*	-3.20	0.01
188	*TSHR*	-2.62	0.02
189	*TTC23*	-0.81	0.04
190	*TUBB4A*	-2.93	0.01
191	*UGT1A1*	-2.38	0.01
192	*UMOD*	-4.45	0.00
193	*VIT*	-4.33	0.04
194	*WNT5A*	-5.97	0.00
195	*ZBTB49*	-1.03	0.01
196	*ZBTB7C*	-1.43	0.02
197	*ZIC4*	-4.75	0.00
198	*ZNF195*	0.77	0.01
199	*ZNF256*	-1.20	0.05
200	*ZNF536*	-3.27	0.03
201	*ZNF541*	-3.04	0.05
202	*ZNF774*	-1.88	0.04

Table S5: List of miRNAs binding to *DSCAM-AS1*

S.No.	miRNA	Target Gene
1	*hsa-miR-627-3p*	*DSCAM-AS1*
2	*hsa-miR-6867-5p*	*DSCAM-AS1*
3	*hsa-miR-8082*	*DSCAM-AS1*
4	*hsa-miR-149-3p*	*DSCAM-AS1*
5	*hsa-miR-6129*	*DSCAM-AS1*
6	*hsa-miR-7154-5p*	*DSCAM-AS1*
7	*hsa-miR-4510*	*DSCAM-AS1*
8	*hsa-miR-6127*	*DSCAM-AS1*
9	*hsa-miR-3658*	*DSCAM-AS1*
10	*hsa-miR-539-5p*	*DSCAM-AS1*
11	*hsa-miR-6130*	*DSCAM-AS1*
12	*hsa-miR-5003-3p*	*DSCAM-AS1*
13	*hsa-miR-4659b-5p*	*DSCAM-AS1*
14	*hsa-miR-6748-5p*	*DSCAM-AS1*
15	*hsa-miR-6787-3p*	*DSCAM-AS1*
16	*hsa-miR-2681-3p*	*DSCAM-AS1*
17	*hsa-miR-4758-5p*	*DSCAM-AS1*
18	*hsa-miR-3163*	*DSCAM-AS1*
19	*hsa-miR-186-5p*	*DSCAM-AS1*
20	*hsa-miR-4667-3p*	*DSCAM-AS1*
21	*hsa-miR-6874-5p*	*DSCAM-AS1*
22	*hsa-miR-5590-3p*	*DSCAM-AS1*
23	*hsa-miR-4297*	*DSCAM-AS1*
24	*hsa-miR-3922-3p*	*DSCAM-AS1*
25	*hsa-miR-4694-3p*	*DSCAM-AS1*
26	*hsa-miR-147a*	*DSCAM-AS1*
27	*hsa-miR-548x-3p*	*DSCAM-AS1*
28	*hsa-miR-548aj-3p*	*DSCAM-AS1*
29	*hsa-miR-7974*	*DSCAM-AS1*
30	*hsa-miR-3174*	*DSCAM-AS1*
31	*hsa-miR-122-5p*	*DSCAM-AS1*
32	*hsa-miR-582-3p*	*DSCAM-AS1*
33	*hsa-miR-335-3p*	*DSCAM-AS1*
34	*hsa-miR-4646-5p*	*DSCAM-AS1*
35	*hsa-miR-181b-5p*	*DSCAM-AS1*
36	*hsa-miR-4294*	*DSCAM-AS1*
37	*hsa-miR-6797-5p*	*DSCAM-AS1*
38	*hsa-miR-6825-5p*	*DSCAM-AS1*
39	*hsa-miR-4726-3p*	*DSCAM-AS1*
40	*hsa-miR-1238-5p*	*DSCAM-AS1*
41	*hsa-miR-4534*	*DSCAM-AS1*
42	*hsa-miR-490-5p*	*DSCAM-AS1*
43	*hsa-miR-504-3p*	*DSCAM-AS1*

44	*hsa-miR-4311*	*DSCAM-AS1*
45	*hsa-miR-5680*	*DSCAM-AS1*
46	*hsa-miR-181d-5p*	*DSCAM-AS1*
47	*hsa-miR-6124*	*DSCAM-AS1*
48	*hsa-miR-574-5p*	*DSCAM-AS1*
49	*hsa-miR-23b-5p*	*DSCAM-AS1*
50	*hsa-miR-4669*	*DSCAM-AS1*
51	*hsa-miR-1250-3p*	*DSCAM-AS1*
52	*hsa-miR-2392*	*DSCAM-AS1*
53	*hsa-miR-7157-3p*	*DSCAM-AS1*
54	*hsa-miR-4728-5p*	*DSCAM-AS1*
55	*hsa-miR-583*	*DSCAM-AS1*
56	*hsa-miR-4786-5p*	*DSCAM-AS1*
57	*hsa-miR-2355-3p*	*DSCAM-AS1*
58	*hsa-miR-5700*	*DSCAM-AS1*
59	*hsa-miR-509-3p*	*DSCAM-AS1*
60	*hsa-miR-6849-5p*	*DSCAM-AS1*
61	*hsa-miR-5581-5p*	*DSCAM-AS1*
62	*hsa-miR-4701-3p*	*DSCAM-AS1*
63	*hsa-miR-6793-5p*	*DSCAM-AS1*
64	*hsa-miR-3134*	*DSCAM-AS1*
65	*hsa-miR-6133*	*DSCAM-AS1*
66	*hsa-miR-3976*	*DSCAM-AS1*
67	*hsa-miR-5692a*	*DSCAM-AS1*
68	*hsa-miR-208a-5p*	*DSCAM-AS1*
69	*hsa-miR-1976*	*DSCAM-AS1*
70	*hsa-miR-495-3p*	*DSCAM-AS1*
71	*hsa-miR-129-5p*	*DSCAM-AS1*
72	*hsa-miR-4695-3p*	*DSCAM-AS1*
73	*hsa-miR-4419a*	*DSCAM-AS1*
74	*hsa-miR-6506-5p*	*DSCAM-AS1*
75	*hsa-miR-212-5p*	*DSCAM-AS1*
76	*hsa-miR-6879-3p*	*DSCAM-AS1*
77	*hsa-miR-3126-5p*	*DSCAM-AS1*
78	*hsa-miR-380-3p*	*DSCAM-AS1*
79	*hsa-miR-4802-3p*	*DSCAM-AS1*
80	*hsa-miR-204-3p*	*DSCAM-AS1*
81	*hsa-miR-3925-5p*	*DSCAM-AS1*
82	*hsa-miR-5590-5p*	*DSCAM-AS1*
83	*hsa-miR-1343-3p*	*DSCAM-AS1*
84	*hsa-miR-6833-3p*	*DSCAM-AS1*
85	*hsa-miR-494-3p*	*DSCAM-AS1*
86	*hsa-miR-4755-3p*	*DSCAM-AS1*
87	*hsa-miR-30a-3p*	*DSCAM-AS1*
88	*hsa-miR-6083*	*DSCAM-AS1*
89	*hsa-miR-6873-5p*	*DSCAM-AS1*

90	*hsa-miR-619-5p*	*DSCAM-AS1*
91	*hsa-miR-4659a-5p*	*DSCAM-AS1*
92	*hsa-miR-3176*	*DSCAM-AS1*
93	*hsa-miR-5703*	*DSCAM-AS1*
94	*hsa-miR-4261*	*DSCAM-AS1*
95	*hsa-miR-6883-5p*	*DSCAM-AS1*
96	*hsa-miR-559*	*DSCAM-AS1*
97	*hsa-miR-6739-5p*	*DSCAM-AS1*
98	*hsa-miR-1263*	*DSCAM-AS1*
99	*hsa-miR-5692c*	*DSCAM-AS1*
100	*hsa-miR-6866-5p*	*DSCAM-AS1*
101	*hsa-miR-6737-3p*	*DSCAM-AS1*
102	*hsa-miR-4762-3p*	*DSCAM-AS1*
103	*hsa-miR-6733-5p*	*DSCAM-AS1*
104	*hsa-miR-19b-2-5p*	*DSCAM-AS1*
105	*hsa-miR-4668-5p*	*DSCAM-AS1*
106	*hsa-miR-19a-5p*	*DSCAM-AS1*
107	*hsa-miR-6832-5p*	*DSCAM-AS1*
108	*hsa-miR-4422*	*DSCAM-AS1*
109	*hsa-miR-30e-3p*	*DSCAM-AS1*
110	*hsa-miR-299-3p*	*DSCAM-AS1*
111	*hsa-miR-5692b*	*DSCAM-AS1*
112	*hsa-miR-6785-5p*	*DSCAM-AS1*
113	*hsa-miR-6750-5p*	*DSCAM-AS1*
114	*hsa-miR-6844*	*DSCAM-AS1*
115	*hsa-miR-548t-5p*	*DSCAM-AS1*
116	*hsa-miR-1303*	*DSCAM-AS1*
117	*hsa-miR-548az-5p*	*DSCAM-AS1*
118	*hsa-miR-211-5p*	*DSCAM-AS1*
119	*hsa-miR-379-3p*	*DSCAM-AS1*
120	*hsa-miR-19b-1-5p*	*DSCAM-AS1*
121	*hsa-miR-579-3p*	*DSCAM-AS1*
122	*hsa-miR-3686*	*DSCAM-AS1*
123	*hsa-miR-4687-3p*	*DSCAM-AS1*
124	*hsa-miR-30d-3p*	*DSCAM-AS1*
125	*hsa-miR-411-5p*	*DSCAM-AS1*
126	*hsa-miR-664b-3p*	*DSCAM-AS1*
127	*hsa-miR-548u*	*DSCAM-AS1*
128	*hsa-miR-6744-5p*	*DSCAM-AS1*
129	*hsa-miR-411-3p*	*DSCAM-AS1*
130	*hsa-miR-3911*	*DSCAM-AS1*
131	*hsa-miR-4743-3p*	*DSCAM-AS1*
132	*hsa-miR-1225-5p*	*DSCAM-AS1*
133	*hsa-miR-153-5p*	*DSCAM-AS1*
134	*hsa-miR-1229-3p*	*DSCAM-AS1*
135	*hsa-miR-204-5p*	*DSCAM-AS1*

136	*hsa-miR-3121-5p*	*DSCAM-AS1*
137	*hsa-miR-548at-5p*	*DSCAM-AS1*
138	*hsa-miR-4779*	*DSCAM-AS1*
139	*hsa-miR-4262*	*DSCAM-AS1*
140	*hsa-miR-1249-5p*	*DSCAM-AS1*
141	*hsa-miR-7856-5p*	*DSCAM-AS1*
142	*hsa-miR-483-5p*	*DSCAM-AS1*
143	*hsa-miR-610*	*DSCAM-AS1*
144	*hsa-miR-9-3p*	*DSCAM-AS1*
145	*hsa-miR-3149*	*DSCAM-AS1*
146	*hsa-miR-6758-5p*	*DSCAM-AS1*
147	*hsa-miR-3135b*	*DSCAM-AS1*
148	*hsa-miR-2054*	*DSCAM-AS1*
149	*hsa-miR-150-3p*	*DSCAM-AS1*
150	*hsa-miR-181a-5p*	*DSCAM-AS1*
151	*hsa-miR-130a-3p*	*DSCAM-AS1*
152	*hsa-miR-301a-3p*	*DSCAM-AS1*
153	*hsa-miR-193b-3p*	*DSCAM-AS1*
154	*hsa-miR-27a-3p*	*DSCAM-AS1*
155	*hsa-miR-204-5p*	*DSCAM-AS1*
156	*hsa-miR-137*	*DSCAM-AS1*
157	*hsa-miR188-5p*	*DSCAM-AS1*
158	*hsa-miR-186-5p*	*DSCAM-AS1*
159	*hsa-miR-101*	*DSCAM-AS1*
160	*hsa-miR-384*	*DSCAM-AS1*
161	*hsa-miR-150-5p*	*DSCAM-AS1*
162	*hsa-miR-2467-3p*	*DSCAM-AS1*
163	*hsa-miR-338-3p*	*DSCAM-AS1*
164	*hsa-miR-877-5p*	*DSCAM-AS1*
165	*hsa-miR-204*	*DSCAM-AS1*
166	*hsa-miR-101-3p*	*DSCAM-AS1*
167	*hsa-miR-122-5p*	*DSCAM-AS1*
168	*hsa-miR-216b*	*DSCAM-AS1*
169	*hsa-miR-136*	*DSCAM-AS1*
170	*hsa-mir-577*	*DSCAM-AS1*

Table S6: List of miRNAs targeting 3'-UTR-*ESR1*

S.No.	miRNA	Target Gene
1	*hsa-miR-302c-3p*	*ESR1*
2	*hsa-miR-206*	*ESR1*
3	*hsa-miR-193b-3p*	*ESR1*
4	*hsa-miR-18b-5p*	*ESR1*
5	*hsa-miR-18a-5p*	*ESR1*
6	*hsa-miR-22-3p*	*ESR1*
7	*hsa-miR-19a-3p*	*ESR1*
8	*hsa-miR-29b-3p*	*ESR1*
9	*hsa-miR-19b-3p*	*ESR1*
10	*hsa-miR-20b-5p*	*ESR1*
11	*hsa-miR-221-3p*	*ESR1*
12	*hsa-miR-222-3p*	*ESR1*
13	*hsa-miR-130a-3p*	*ESR1*
14	*hsa-miR-26a-5p*	*ESR1*
15	*hsa-miR-145-5p*	*ESR1*
16	*hsa-miR-3668*	*ESR1*
17	*hsa-miR-4671-3p*	*ESR1*
18	*hsa-miR-4709-3p*	*ESR1*
19	*hsa-miR-7852-3p*	*ESR1*
20	*hsa-miR-100-5p*	*ESR1*
21	*hsa-miR-142-3p*	*ESR1*
22	*hsa-miR-6073*	*ESR1*
23	*hsa-miR-6757-3p*	*ESR1*
24	*hsa-miR-4422*	*ESR1*
25	*hsa-miR-548e-5p*	*ESR1*
26	*hsa-miR-454-3p*	*ESR1*
27	*hsa-miR-130b-3p*	*ESR1*
28	*hsa-miR-301a-3p*	*ESR1*
29	*hsa-miR-301b-3p*	*ESR1*
30	*hsa-miR-4295*	*ESR1*
31	*hsa-miR-3666*	*ESR1*
32	*hsa-miR-129-5p*	*ESR1*
33	*hsa-miR-874-3p*	*ESR1*
34	*hsa-miR-3157-3p*	*ESR1*
35	*hsa-miR-4290*	*ESR1*
36	*hsa-miR-5582-3p*	*ESR1*
37	*hsa-miR-548x-3p*	*ESR1*
38	*hsa-miR-548j-3p*	*ESR1*
39	*hsa-miR-548aq-3p*	*ESR1*
40	*hsa-miR-548am-3p*	*ESR1*
41	*hsa-miR-548aj-3p*	*ESR1*
42	*hsa-miR-548ah-3p*	*ESR1*
43	*hsa-miR-548ae-3p*	*ESR1*

44	*hsa-miR-5589-3p*	*ESR1*
45	*hsa-miR-520e*	*ESR1*
46	*hsa-miR-520d-3p*	*ESR1*
47	*hsa-miR-520c-3p*	*ESR1*
48	*hsa-miR-520b*	*ESR1*
49	*hsa-miR-520a-3p*	*ESR1*
50	*hsa-miR-373-3p*	*ESR1*
51	*hsa-miR-372-3p*	*ESR1*
52	*hsa-miR-302e*	*ESR1*
53	*hsa-miR-302d-3p*	*ESR1*
54	*hsa-miR-302b-3p*	*ESR1*
55	*hsa-miR-302a-3p*	*ESR1*
56	*hsa-miR-519c-3p*	*ESR1*
57	*hsa-miR-519b-3p*	*ESR1*
58	*hsa-miR-519a-3p*	*ESR1*
59	*hsa-miR-548z*	*ESR1*
60	*hsa-miR-548h-3p*	*ESR1*
61	*hsa-miR-548d-3p*	*ESR1*
62	*hsa-miR-548bb-3p*	*ESR1*
63	*hsa-miR-548ac*	*ESR1*
64	*hsa-miR-7855-5p*	*ESR1*
65	*hsa-miR-9-5p*	*ESR1*
66	*hsa-miR-4264*	*ESR1*
67	*hsa-miR-4797-3p*	*ESR1*
68	*hsa-miR-3121-5p*	*ESR1*
69	*hsa-miR-1910-5p*	*ESR1*
70	*hsa-miR-26b-3p*	*ESR1*
71	*hsa-miR-192-5p*	*ESR1*
72	*hsa-miR-335-3p*	*ESR1*
